# *In Silico* Analysis of the Metabolic Potential and Niche Specialization of Candidate Phylum "*Latescibacteria*" (WS3)

**DOI:** 10.1371/journal.pone.0127499

**Published:** 2015-06-03

**Authors:** Noha H. Youssef, Ibrahim F. Farag, Christian Rinke, Steven J. Hallam, Tanja Woyke, Mostafa S. Elshahed

**Affiliations:** 1 Department of Microbiology and Molecular Genetics, Oklahoma State University Stillwater, Oklahoma, United States of America; 2 DOE Joint Genome Institute, Walnut Creek, California, United States of America; 3 University of British Columbia, Vancouver, BC, Canada; 4 Graduate Program in Bioinformatics, University of British Columbia, Vancouver, BC, Canada; Cairo University, EGYPT

## Abstract

The “*Latescibacteria*” (formerly WS3), member of the Fibrobacteres–Chlorobi–Bacteroidetes (FCB) superphylum, represents a ubiquitous *candidate phylum found in* terrestrial, aquatic, and marine ecosystems. Recently, single-cell amplified genomes (SAGs) representing the “*Latescibacteria*” were obtained from the anoxic monimolimnion layers of Sakinaw Lake (British Columbia, Canada), and anoxic sediments of a coastal lagoon (Etoliko lagoon, Western Greece). Here, we present a detailed *in-silico* analysis of the four SAGs to gain some insights on their metabolic potential and apparent ecological roles. Metabolic reconstruction suggests an anaerobic fermentative mode of metabolism, as well as the capability to degrade multiple polysaccharides and glycoproteins that represent integral components of green (Charophyta and Chlorophyta) and brown (Phaeophycaea) algae cell walls (pectin, alginate, ulvan, fucan, hydroxyproline-rich glycoproteins), storage molecules (starch and trehalose), and extracellular polymeric substances (EPSs). The analyzed SAGs also encode dedicated transporters for the uptake of produced sugars and amino acids/oligopeptides, as well as an extensive machinery for the catabolism of all transported sugars, including the production of a bacterial microcompartment (BMC) to sequester propionaldehyde, a toxic intermediate produced during fucose and rhamnose metabolism. Finally, genes for the formation of gas vesicles, flagella, type IV pili, and oxidative stress response were found, features that could aid in cellular association with algal detritus. Collectively, these results indicate that the analyzed *“Latescibacteria”* mediate the turnover of multiple complex organic polymers of algal origin that reach deeper anoxic/microoxic habitats in lakes and lagoons. The implications of such process on our understanding of niche specialization in microbial communities mediating organic carbon turnover in stratified water bodies are discussed.

## Introduction

Over the past few decades, small subunit ribosomal RNA (SSU or 16S rRNA) gene-based surveys have prompted a drastic reevaluation of the scope of phylum level diversity within the domain Bacteria. Current taxonomic outlines indicate that the majority of recognized bacterial phyla (54.1% using SILVA database [1], 65.48% using Greengenes database [2]) have no pure culture representatives (candidate phyla). Many of these candidate phyla, so-called microbial dark matter (MDM) are globally distributed and display significant levels of intra-phylum level diversity [3–7]. Recent advances in cell sorting and whole genome amplification and assembly have facilitated the acquisition of single-cell amplified genomes (SAGs) derived from numerous candidate phyla [8–16]. Metabolic reconstruction with these SAGs provides a unique opportunity to uncover the ecological and biogeochemical roles played by these enigmatic microbial groups.

One such candidate phylum is WS3 (Wurtsmith aquifer Sequences-3), whose members were first identified in a 16S rRNA gene-based survey of anoxic sediments obtained from a hydrocarbon- and chlorinated-solvents-contaminated aquifer in northern Michigan, USA in 1998 [17]. Since then, their presence has been documented across a wide range of habitats including marine hydrothermal vents, gas hydrate-bearing habitats, cold methane seeps, cave rock walls, marine sediments, soils, wastewater treatment bioreactors, deep sea hypersaline anoxic lakes, and oil-exposed microbial mats [18–28]. Recently, as part of an extensive single cell genomic study of 9 different habitats, Rinke et al. [29] reported on the recovery of four SAGs from WS3 single cells. Phylogenomic-based analysis using conserved marker genes indicated the monophyletic nature of WS3 as part of the Fibrobacteres–Chlorobi–Bacteroidetes (FCB) superphylum together with “Marinimicrobia” (SAR406), “Cloacimonetes” (WWE1), Gemmatimonadetes, and Caldithrix. The name “*Latescibacteria*” (hiding small rods) was suggested for the candidate phylum.

However, little is known about the biological capabilities of this phylum, and no systematic attempts have been made to reconstruct its metabolic potential. Thus, we here present a detailed analysis of the metabolic and physiological capabilities, and putative ecological roles of four “*Latescibacteria*” SAGs obtained from two different aquatic environments. Our analysis suggests that the *“Latescibacteria”* recovered from Sakinaw Lake and Etoliko lagoon transform algal detritus sinking from sunlit surface waters into fermentation products with the potential to contribute to microbial food webs in anaerobic waters below.

## Materials and Methods

### Origin of “*Latescibacteria*” SAGs

*“Latescibacteria”* SAGs analyzed in this study were obtained from two different locations [29]: Three SAGs originated from a single sample obtained from the anaerobic monimolimnion of Sakinaw lake (British Columbia, Canada) at 49°40'30"N, 124°2'2.4"W coordinates, and a depth of 120m (Gies et al 2014). A fourth SAG was obtained by sampling anaerobic sediments in Etoliko Lagoon, a coastal lagoon in the south of Aetolia-Acarnania, Greece, at the deepest point (~27.5 m) at 38°28'59.54"N, 21°19'17.44"E. Single cell sorting and lysis, whole genome amplification, identification via 16S rRNA gene sequencing of amplified genomes, as well as SAG sequencing, assemblies and estimates of genome completion were previously described [29]. The four *“Latescibacteria”* SAGs were deposited under Genbank assembly IDs: NZ_ASMB00000000.1, NZ_AQSL00000000.1, ASWY00000000.1, and AQRO00000000.1, and in Integrated Microbial Genomics (IMG) under SAG IDs: SCGC AAA252-D10, SCGC AAA252-B13 and SCGC AAA252-E07 for Sakinaw lake SAGs, and SCGC AAA257-K07 for the Etoliko lagoon SAG. These SAGs will henceforth be referred to as S-D10, S-B13, and S-E07 for Sakinaw Lake SAGs, and E-K07 for Etoliko Lagoon SAG. The type species for”*Latescibacteria*” is S-E07, for which the name *Candidatus* “Latescibacter anaerobius” has been proposed [29].

Detailed analysis was conducted on S-E07, which has the highest estimated genome completion (73.02%) among the *“Latescibacteria”* SAGs. The closely related S-B13 (94% 16S rRNA gene sequence similarity to SAG S-E07) with 57.1% estimated genome completion was used to confirm shared gene content and fill pathway holes when needed. Only general metabolic features for SAG S-D10 (94% 16S rRNA gene sequence similarity to S-E07, and 96% to S-B13) are discussed, given its low percentage of estimated genome completeness (38.2%). Due to the observed differences between the 3 Sakinaw Lake SAGs, and the Etoliko lagoon SAG E-K07 (85–86% 16S rRNA gene sequence similarity to Sakinaw Lake SAGs), as well as its low estimated genome completion (23.02%), analysis of SAG E-K07 was restricted to identifying variation in conserved genes or pathways between “*Latescibacteria*” SAGs from two distinct locations.

### Genome annotation, general genomic features, and metabolic reconstruction

The IMG platform (http://img.jgi.doe.gov) was used for genome functional annotation. Detailed metabolic reconstruction of relevant pathways was performed with both KEGG [30] and Metacyc [31] databases. As part of the IMG annotation pipeline, CRISPR elements are detected with CRT [32] and PILERCR [33]. Predictions from both methods are concatenated and in case of overlapping elements, the shorter one is removed. Overall annotation followed procedures outlined in [13]: In brief, proteases, peptidases, and protease inhibitors were identified with Blastp against the Merops database [34]. Transporters were identified with the transporter classification database (TCDB) [35]. dbCAN HMMs [36] were used to identify carbohydrate active enzymes (CAZymes) including glycoside hydrolases (GH), polysaccharide lyases (PL), and carboxyl esterases (CE) following the classification scheme of the Carbohydrate active enzyme (CAZy) database [37].

## Results

### Phylogenetic affiliation and general genomic features of “*Latescibacteria*” SAGs

**Fig 1:**
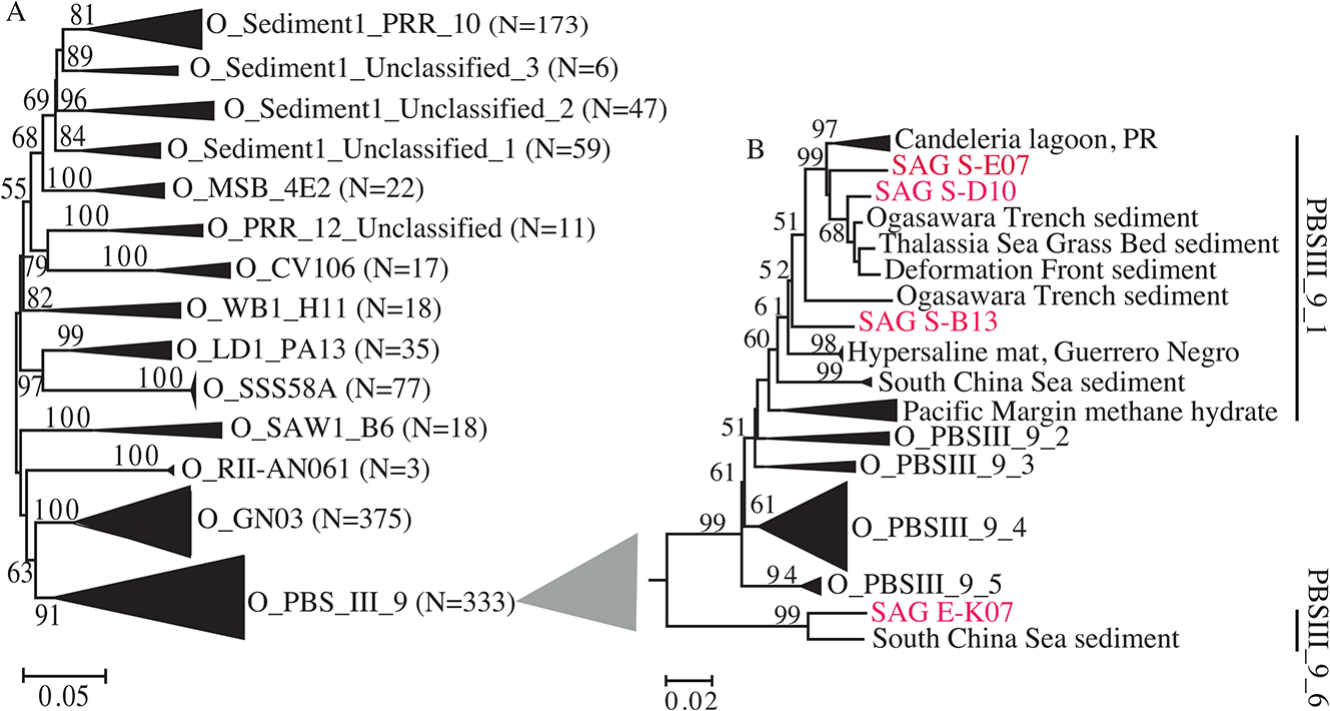
Updated taxonomic outline for candidate phylum “*Latescibacteria*” (A), and for the candidate order PBSIII_9 (B). Neighbor joining trees were constructed using Jukes-Cantor corrections in MEGA6-Beta2 [100]. Bootstrap values (in percent) are based on 1000 replicates and are shown for branches with more than 50% bootstrap support. Numbers in parentheses represent the number of sequences in each WS3 candidate order.

All four SAGS were affiliated with the candidate order PBS_III_9 based on phylogenetic analysis of the candidate phylum “*Latescibacteria*” using 1198 near-full length 16S rRNA gene sequences (Fig 1, Table A in S1 File, and Supplementary Text in S1 File). Sakinaw lake SAGs belonged to family I, while Etoliko lagoon SAG E-K07 belonged to family VI within this order (Fig 1B). General genomic features for each SAG are shown in Table 1.

**Table 1:** General genomic features of “*Latescibacteria”* SAGs.

	SAGs from Sakinaw Lake			SAG from Etoliko Lagoon
	SCGC AAA252-E07	SCGC AAA252-B13	SCGC AAA252-D10	SCGC AAA257-K07
Genome size, Mb	2.3	1.49	0.5	1.77
Estimated genome completeness, %	73.02	57.09	38.17	23.02
Estimated size, Mb	3.15	2.61	1.31	7.69
GC %	42.07	40.86	40.86	42.12
% Non coding DNA	14.6	15.1	16.6	13.8
Average gene length, bp	988	947	762	980
RNA genes				
5S rRNA Count	1	1	1	1
16S rRNA Count	1	0*	1	1
23S rRNA Count	1	1	1	1
tRNA Count	27	18	10	19
Number of CDS	1951	1558	647	1534
with function prediction	1451	1158	433	1073
without function prediction	500	400	214	461

* The S-B13 16S rRNA couldn’t be retrieved via the whole genome shotgun approach, however the affiliation of S-B13 to CP-“*Latescibateria”* was confirmed through analyzing the amplified and Sanger-sequenced full-length 16S rRNA gene

### Metabolic potential of Sakinaw Lake SAGs

Anabolic pathways identified in S-E07 and S-B13 include machinery for the production of amino acids, cofactors, fatty acids, purines and pyrimidines, terpenoid unit backbone, and glycerophospholipids. In addition, the SAGs encode near-complete replication, transcriptional, and translational machineries. The presence of genes for lipopolysaccharide (LPS) biosynthesis and pathway for LPS insertion in the outer membrane suggests a Gram-negative cell wall (Supplementary Text).

**Fig 2:**
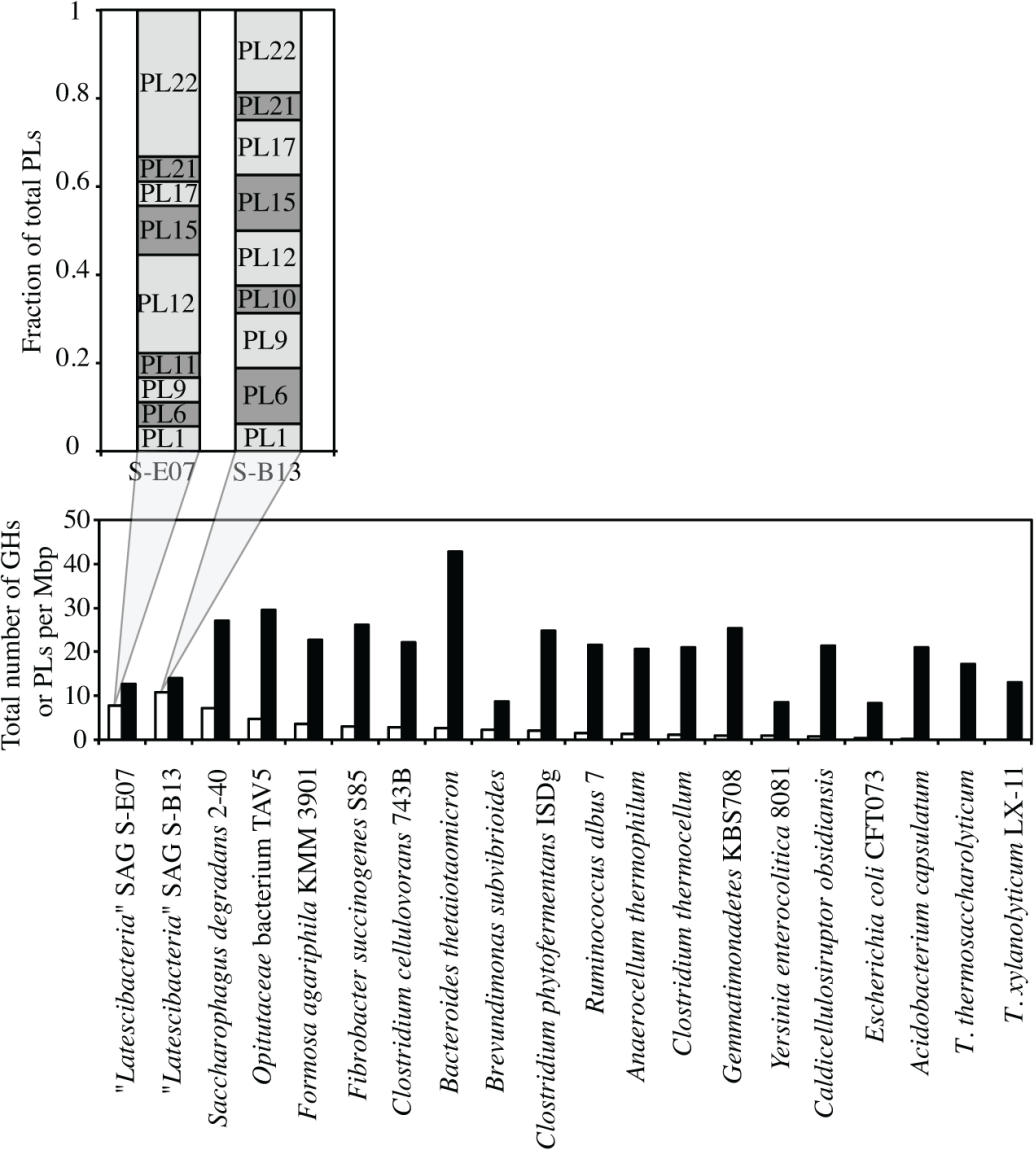
Total number of PLs (white columns) and GHs (black columns) per Mbp of various pectinolytic and lignocellulolytic microorganisms’ genomes. Note that, compared to other genomes, “*Latescibacteria*” SAGS are enriched in PLs as opposed to GHs. The inset shows SAGs S-E07 and S-B13 different PL families as a fraction of total PLs.

Catabolical pathways identified in S-E07 and S-B13 indicate a heterotrophic lifestyle. Moreover, the apparent absence of a respiratory chain suggests sole dependence on fermentative pathways and substrate level phosphorylation for coupled energy release and conservation. Both S-E07 and S-B13 encode a diverse array of carbohydrate active enzymes (CAZymes), with a conspicuous enrichment (Genes/Mbp), and diversity (number of different families) of polysaccharide lyases (PLs) (Fig 2, Figure A in S1 File, Table B in S1 File). In contrast, the SAGs are relatively depauperate in genes encoding glycoside hydrolases (GHs) including enzymes involved in the degradation of cellulose (1 putative endoglucanase (GH5), 3 putative β-glucosidases (GH3, GH116, and GH9), and no putative cellobiohydrolase), and enzymes involved in the degradation of xylans (xylanases, andβ-xylosidases).

**Fig 3:**
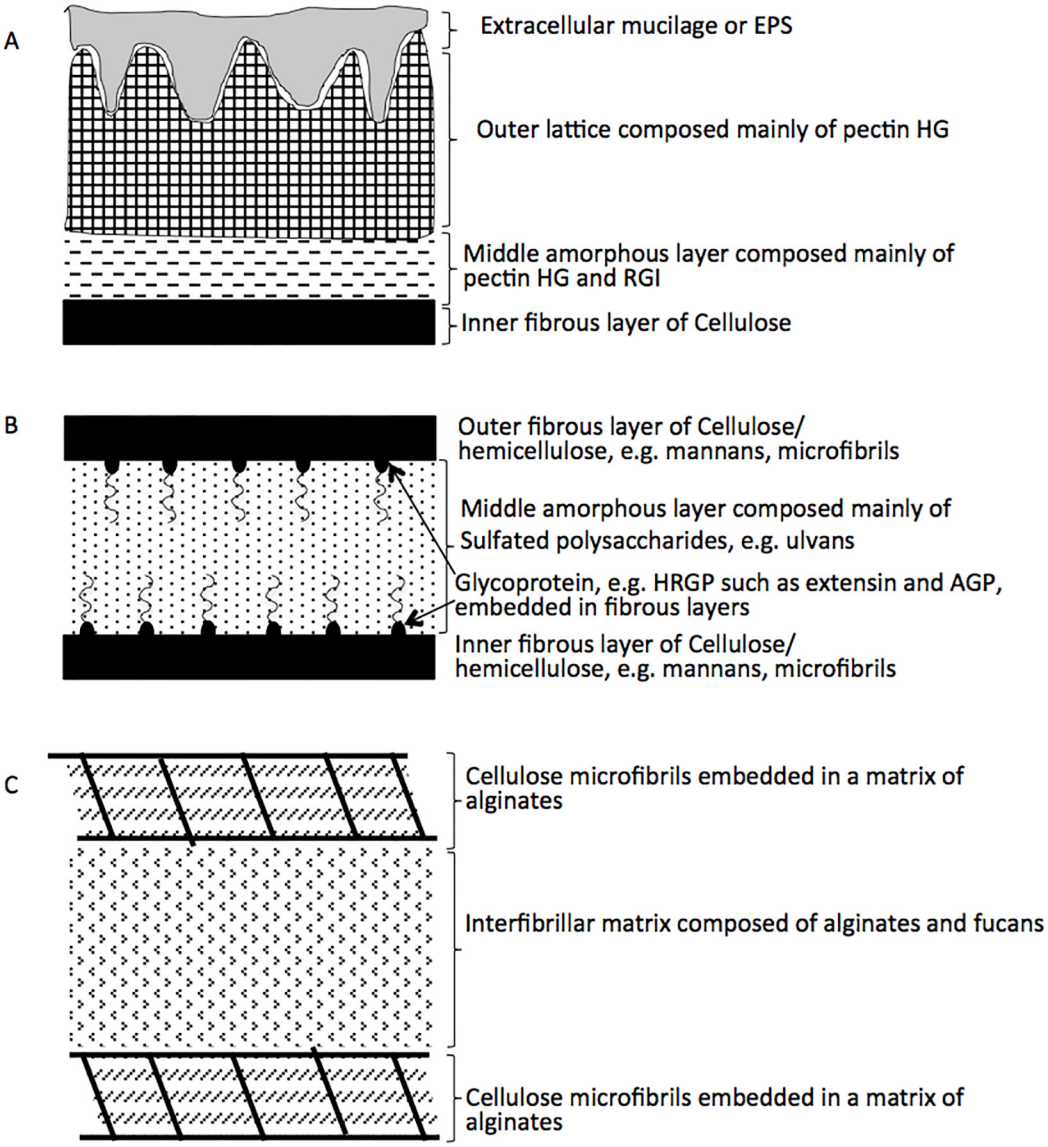
Schematic representation of algal cell walls. The cell wall composition differs between various algal groups [43]. Within the Charophyta (A), the wall is formed of an inner fibrillar layer made of cellulose microfibrils. The fibrillar layer is enmeshed in and surrounded by a middle amorphous matrix of pectin (homogalacturonan, HG, and rhamnogalacturonan I, RGI) that anchors the inner fibrillar cellulose layer to an outer lattice of homogalacturonan. Extracellular polymeric substances or mucilages are also present outside the outer lattice [38, 43, 101]. Similarly, cell walls of Chlorophyta (B) contain skeletal polysaccharides enmeshed in a matrix. However, the skeletal polysaccharides in Chlorophyta cell walls form double fibrillar layers (inner layer and outer layer) with an amorphous matrix in between. The fibrillar layers vary in composition between cellulose, β-1,3-xylans or β-1,4-mannans or complex heteropolymers, and are rich in hydroproline-rich glycoprotein such as extensins and AGPs. The amorphous matrix polysaccharides are generally in the form of ulvans (e.g. in Ulva species). Brown algal cell walls (C) consist of a fibrillar framework of cellulose microfibrils present in layers parallel to the cell surface but with no clear orientation within each layer. Two such layers are depicted in the figure. All cellulose layers are enmeshed in acidic polysaccharides, e.g. alginates. The interfibrillar matrices are composed of alginates and fucans [41, 43]**.**

Interestingly, many of the polymers that S-E07 and S-B13 are predicted to degrade are integral components of cell walls of the green algal phyla Charophyta (most commonly encountered in freshwater habitats), and Chlorophyta (widely distributed in freshwater, marine, and terrestrial habitats), as well as the brown algal Class Phaeophyceae. Green and brown algal cell walls are complex, with a diverse array of structural fibrillar polymers enmeshed in complex matrices with crystalline polymer components (Fig 3). Both S-E07 and S-B13 encode genes necessary for the conversion of these cell wall components, including pectin, alginate, ulvans, fucans, hydroxyproline-rich glycoproteins (HRGP), e.g. arabinogalactan proteins (AGP) and extensins, and xyloglucan (Table 2, Figure B in S1 File, Supplementary Text in S1 File). Moreover, the SAGs also encode pathways mediating the conversion of soluble organic compounds commonly utilized for storage in algae (e.g. starch and trehalose). A more in depth description of these capabilities follows.

**Table 2:** Polymers potentially targeted by Latescibacteria, their distribution and occurrence in algae, structure, degradation enzymes encoded in the Latescibacterial SAGs, potential degradation products, their transport systems encoded in the SAGs, and ultimate central catabolic pathway.

Polymer	Distribution	Degradation	Products	Transport system	Central pathway
1. Pectin					
Homogalacturonan	Land plants and Charophyta green algae	Pectin methylesterase	Pectinate/ Pectate (demethylated)		EMP
		Pectin acetylesterase	Deacetylated polymer		
		Pectin lyase, pectate lyase	Oligosaccharides with 4-deoxy-α-D-galact-4-enuronosyl groups at their non-reducing ends		
		Exopolygalacturonate lyase	digalacturonate		
		Oligogalacturonide lyase	5-dehydro-4-deoxy-D-glucuronate	ExuT symporter	
			galacturonic acid		
Rhamnogalacturonan I		Pectin methylesterase	Demethylated RGI		
		Pectin acetylesterase	Deacetylated RGI		
		Rhamnogalacturonan endolyase	Oligosaccharides with L-rhamnopyranose at the reducing end and 4-deoxy-4,5-unsaturated D-galactopyranosyl uronic acid at the non-reducing end		
		Rhamnogalacturonan exolyase	disaccharide 2-O-(4-deoxy-beta-L-threo-hex-4-enopyranuronosyl)-alpha-L-rhamnopyranose		
		d-4,5-unsaturated β-glucuronyl hydrolase	rhamnose	Rhamnose:proton symporter	BMC
			5-dehydro-4-deoxy-D-glucuronate	ExuT symporter	EMP
		β-galactosidase	Galactose	Galactose:sodium symporter	EMP
Alginates	Brown Algae	Poly β-D-mannuronate lyase	Oligosaccharides with 4-deoxy-α-L-*erythro*-hex-4-enopyranuronosyl groups at their non-reducing ends		
		Oligoalginate lyase	4-deoxy-α-L-erythro-hex-4-enopyranuronose spontaneously converted to 5-dehydro-4-deoxy-D-glucuronate	ExuT symporter	EMP
2. Fucans					
Homofucan	Brown Algae	Sulfatase	Unsulfated homofucans		
		Fucoidan lyase-like protein (hypothetical protein)	Unsaturated, non-sulfated di- and tetrasaccharides		
		α-L-fucosidase	Fucose	Fucose:proton symporter	BMC
Xylofucogalactan/ xylofucomannan		Sulfatase	Unsulfated fucans		
		α-L-fucosidase	Fucose	Fucose:proton symporter	BMC
		β-glucuronidase	Glucuronic acid	ExuT symporter	EMP
Xylofucoglucuronan		Sulfatase	Unsulfated fucans		
		α-L-fucosidase	Fucose	Fucose:proton symporter	BMC
3. Ulvans	Chlorophyta	Sulfatase	Unsulfated ulvans		
		Heparin lyase	Unsaturated, non-sulfated di- and tetrasaccharides		
		d-4,5-unsaturated β-glucuronyl hydrolase	5-dehydro-4-deoxy-D-glucuronate	ExuT symporter	EMP
			Rhamnose	Rhamnose:proton symporter	BMC
			Xylose	Xylose:proton symporter	PPP
4. Xyloglucan	Land plants, some green algae	endo-β-1,4-glucanase	A mixture of oligosaccharides		
		α-1,2-fucosidase	Fucose	Fucose:proton symporter	BMC
		β-galactosidase	Galactose	Galactose:sodium symporter	EMP
		β-glucosidase	Glucose	Glucose:sodium symporter	EMP
5. Hydroxyproline-rich glycoprotein (HRGP)					
Extensin	Land plants, some green algae	β-L-arabinofuranosidase	Arabinose	ABC transporter	PPP
Arabinogalactan protein (AGP)		endo-β-1,6-galactanase ^(?)^	galactan oligosaccharides		
		β-glucuronidase	Glucuronic acid	ExuT symporter	EMP
		α-Fucosidase	Fucose	Fucose:proton symporter	BMC
		α-rhamnosidase	Rhamnose	Rhamnose:proton symporter	BMC
		β-galactosidase	Galactose	Galactose:sodium symporter	EMP
6. Others					
Extracellular proteins	All organisms	Non-specific endopeptidases	Oligopeptides	ABC transporter	
		Dipeptidases	Dipeptides	ABC transporter	
		Dipeptide peptidase, aminopeptidases, or Carboxypeptidases	Free amino acids	Symporters for Pro, Ala, Asp, Glu, Gly, cationic aaABC transporter for Pro	EMP (Asp and Glu)
Starch	Land plants, and green algae storage compounds	α-amylase	Oligosaccharides		
		α-glucosidase	Glucose	Glucose:sodium symporter	EMP
Trehalose	Brown algae storage compound	Trehalase	Glucose	Glucose:sodium symporter	EMP
			Glucose-1-P		
Poly-D-galactosamine	Some fungi such as *Aspergillus*, and *Neurocrassa*	Endo1,4-poly-D-galactosaminidase	Galactosamine	PTS	EMP

### Algal cell wall degradation potential

#### 1. Pectins

Pectins are components of the amorphous matrix and outer lattice of Charophyta cell wall (Fig 3A) [38]. Both S-E07 and S-B13 encode machinery for depolymerizing the pectic polysaccharide homogalacturonan (HG) (Table 2). They encode carboxyl esterases (CE8 and CE12) for the removal of accessory acetyl and methyl groups attached to the backbone, pectin lyase and pectate lyase (PL1, and PL10) to breakdown the backbone to oligosaccharides with 4-deoxy-α-D-galact-4-enuronosyl groups at their non-reducing ends, exopolygalacturonate lyase (PL9) to cleave digalacturonate unit, and oligogalacturonide lyase (PL22) to degrade the digalacturonate units to 5-dehydro-4-deoxy-D-glucuronate and galacturonic acid as the final end products of HG degradation [39, 40]. In addition to HG, S-E07 and S-B13 encode all the necessary machinery to degrade rhamnogalacturonan I (RGI) (Table 2). These include carboxyl esterases (CE8 and CE12), rhamnogalacturonan endolyase (PL11) that attack the backbone to produce oligosaccharides with L-rhamnopyranose at the reducing end and 4-deoxy-4,5-unsaturated D-galactopyranosyl uronic acid at the non-reducing end, rhamnogalacturonan exolyase (PL11) that attacks those oligosaccharides to release the disaccharide 2-O-(4-deoxy-beta-L-threo-hex-4-enopyranuronosyl)-alpha-L-rhamnopyranose from the reducing end, and d-4,5-unsaturated β-glucuronyl hydrolase (GH88) that degrades those disaccharides to rhamnose and 5-dehydro-4-deoxy-D-glucuronate. The SAGs also encode β-galactosidase (GH42) for removal of galactosyl sugar substitutions [39, 40].

#### 2. Alginate

Alginates are present in the brown algal cell walls enmeshing fibrillar cellulose and also in the interfibrillar layers with fucans (Fig 3C) [41]. Both S-E07 and S-B13 encode PLs for the complete degradation of alginate (Table 2). These PLs include alginate lyases (PL6, PL15, PL17) that break down the alginate backbone producing oligosaccharides with 4-deoxy-α-L-*erythro*-hex-4-enopyranuronosyl groups at their non-reducing ends, as well as oligoalginate lyase (PL15, and PL17) that exolytically cleave these oligosaccharides into monosaccharides and releases 4-deoxy-α-L-erythro-hex-4-enopyranuronose from the non-reducing end. The produced 4-deoxy-α-L-erythro-hex-4-enopyranuronose is spontaneously converted into 5-dehydro-4-deoxy-D-glucuronate as the final end product of alginate degradation [42].

#### 3. Fucans

In addition to pectin and alginate, S-E07, and S-B13 also encode machinery for fucan degradation. Fucans are present, together with alginates, in brown algal cell walls interfibrillar matrix (Fig 3C) [41]. Fucans exhibit wide variations in chemical structures, ranging from the highly sulfated homofucan polymers to the highly branched high-uronic-acid, low-sulfate-containing polymers (xylofucoglucan, xylofucogalactan, xylofucomannan, xylofucoglucuronan) [41]. However, mechanistic details on the degradation of fucans are still in their infancy. Genomic analysis of “*Latescibacteria*” SAGs that S-E07, and S-B13 have the capacity to transform several fucans including homofucans, sulfated-xylofucoglucan, and sulfated-xylofucoglucoronan. Indeed, a potential homofucan-degrading enzyme with sequence similarity to *Mariniflexile fucanivorans* fucoidan lyase could attack the backbone releasing unsaturated, non-sulfated fucan di- and tetrasaccharides. The SAGs also encode many α-fucosidases (GH29, and GH95), that could attack those oligosaccharides and release fucosyl residues from the reducing end. Genomic evidence for the degradation of the highly branched high-uronic-acid, low-sulfate-containing polymers include many α-fucosidases (GH29, and GH95), and one α-glucuronidase (GH67).

#### 4. Ulvans

Ulvans are present in the amorphous interfibrillar matrix of Chlorophyta cell walls (Fig 3B) [43–45]. Ulvan backbones are made of a few repeating disaccharides (Supplementary Text). However, the exact composition of ulvans is largely unknown. One important characteristic of ulvans is the presence of unusual sugars, e.g. iduronic acid, in its backbone [44]. Iduronic acid is also an important constituent of mammalian glycosaminoglycans (GAGs), e.g. heparan sulfate, dermatan sulfate, heparin [46]. “*Latescibacteria*” SAGs harbor several PLs annotated as heparin and heparan lyase (PL12 and PL21). Structural similarity in sugar composition between ulvans and mammalian GAGs such as heparin suggest that those polysaccharide lyases (annotated as PL12 and PL21 with heparinase activity) might be potential ulvan lyases responsible for *ulvan backbone cleavage to produce di- and tetrasaccharides* with an unsaturated β-glucuronyl residue located at the non-reducing end [47]. SAGs also harbor several copies of unsaturated glucuronyl hydrolases (GH88) that could potentially act on the oligosaccharides produced and release 5-dehydro-4-deoxy-D-glucuronate and other sugar residues, e.g. rhamnose, and xylose, as end products.

#### 5. Xyloglucan

Xyloglucan is a component of Charophyta and Chlorophyta cell wall usually present in association with cellulose microfibrils (Fig 3A and 3B) [48–50]. Both S-E07 and S-B13 encode machinery to degrade xyloglucan, a component of Charophyta and Chlorophyta cell walls usually present in association with cellulose microfibrils (Fig 3A and 3B) [48–50], including endo-β-1,4-glucanases (GH74), that cleave the xyloglucan backbone at locations of unsubstituted glycosyl moieties and give rise to a mixture of oligosaccharides, α-1,2-fucosidase (GH95), and β-galactosidases (GH2, GH42) that attack those oligosaccharides to give rise to XXXG xyloglucans. The latter oligosaccharide can be attacked by oligoxyloglucan β-glycosidase (GH3) generating isoprimeverose (Xyl-α(1,6)-Glu), and glucose. However, no homologs of oligoxyloglucan β-glycosidase were identified.

#### 6. Hydroxyproline-rich, other O-linked, and N-linked glycoproteins

Hydroxyproline-rich glycoproteins (HRGP) are minor components in green algal cell walls (Fig 3) [51, 52]. Both S-E07, and S-B13 SAGs encode β-L-arabinofuranosidase (GH127) that specifically targets arabinose residues attached to hydroxyproline in extensins [53] and release the sugar monomer arabinose. The SAGs also encode machinery for arabinogalactan protein (AGP) degradation including endo-β-1,6-galactanases (GH30) that hydrolyses the β-1,6-galactan side chains and gives rise to galactan oligosaccharides, β-galactosidases (GH2, GH42), β-glucuronidase (GH79), α-fucosidase (GH29, GH95), and α-rhamnosidase (GH28, GH78, GH106) that attack the produced oligosaccharides and release substituting sugar monomers, e.g. galactose, glucuronic acid, fucose, and rhamnose [54]. In addition to HRGP degradation potential, the SAGs encode several α-N-acetylgalactosaminidases (GH109) that specifically release N-acetylgalactosaminyl residues from O-linked glycoproteins [55], as well as several α-mannosidases (GH38) that could potentially release mannosyl residues from N-linked glycoproteins [56]. Recently, sialic acid (neuraminic acid), a 9-carbon sugar acid was identified in green algal N-linked glycoproteins [57]. While a sialidase (GH33) homologue was not identified in the SAGs, they do encode for all the enzymes required for sialic acid degradation, including sialate O-acetylesterase, N-acetylneuraminate lyase, and N-acyl-D-glucosamine 2-epimerase that will collectively degrade sialic acid into pyruvate and N-acetyl-glucosamine (NAG).

#### 7. Degradation of cell wall proteins

Both S-E07 and S-B13 SAGs encode multiple peptidases that can attack the peptide moiety of glycoproteins in algal cell walls (Table C in S1 File). The majority of these peptidases (~66% in S-E07, and 63.4% in S-B13) are thought to be nutritional, where they non-specifically break down proteins into oligopeptides (protease families C25, M06, M10, M20, M41, M48, M50, S01, S08, S09, S41, S54, and U62), dipeptides (protease family M19), and free amino acids (protease families M24, M28, S49, T03).

#### 8. Sulfatase activity on sulfated polysaccharide

Both S-E07 and S-B13 encode multiple sulfatases (n = 14 in S-E07 and n = 3 in S-B13) belonging to the family of arylsulfatases (pfam 00884). Many of the polymers in marine algal cell walls are sulfated, e.g. ulvans, homofucans, sulfated-xylofucoglucan, and sulfated-xylofucoglucoronan [58]. Removal of the sulfate groups from such polysaccharides prior to their degradation facilitates access of GHs and PLs to side chains and backbones [59]. The SAGs also harbor the essential anaerobic sulfatase maturation enzyme-coding gene [60] for post-translational modification of a critical Cys or Ser in the active site to a C-α-formylglycine [61].

### Degradation of algal storage compounds and additional polymers of non-algal origin

In addition to algal cell wall components, both S-E07 and S-B13 encode GHs that could potentially target algal intracellular carbon storage compounds, or secreted polysaccharides sourced from other organisms. The SAGs encode GHs specific for starch (α-amylase belonging to GH119, GH57, GH13, and α-glucosidase belonging to GH97), as well as for trehalose (trehalase/maltase belonging to GH65) degradation. Starch is recognized as an important intracellular storage compound in green algae and green plants [62], while trehalose is an intracellular storage compound in brown algae [41]. In addition, S-E07 and S-B13 encode β-fructofuranosidase (GH32) specific for sucrose, and endo1,4-poly-D-galactosaminidase (GH114) specific for poly-D-galactosamine (Table 2).

### Extracellular polymeric substance (EPS) as additional potential source of energy for the “*Latescibacteria*”

EPS forms extensive mucilaginous sheath outside the algal cell wall and function in adhesion, gliding motility, biofilm formation, and protection. Although the exact chemistry of EPS is not entirely known, EPS was shown to be composed mainly of polysaccharides (up to 75%), with minor protein content (2–10%). The polysaccharide fraction is rich in uronic acids, as well as monosaccharides, mainly glucose, galactose, mannose, xylose, arabinose, fucose, and rhamnose [63, 64]. As mentioned above, “*Latescibacteria*” SAGs harbor genes involved in the uptake and catabolism of all such components.

### “*Latescibacteria*” SAGs harbor extensive transport systems for sugars, and amino acids/oligopeptides uptake

**Fig 4:**
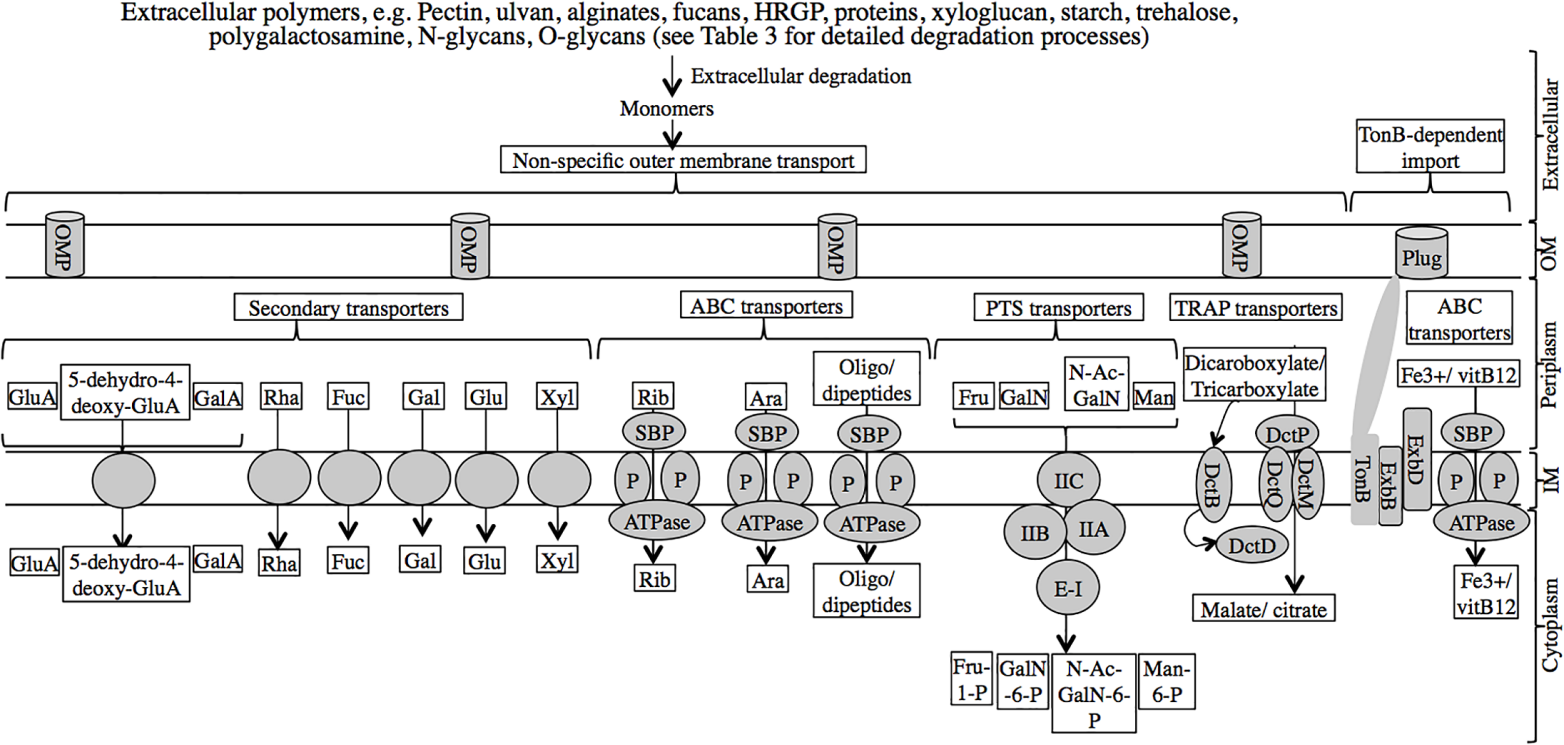
Import systems in “*Latescibacteria*” predicted from the SAGs. Extracellular degradation of polymers, as detailed in Table 2, results in the production of monomers that could potentially be transported across the outer membrane (OM) of “*Latescibacteria*” cell wall through non-specific outer membrane porins (OMP). In the periplasm, those monomers are then transported across the inner membrane (IM) via dedicated transporters including (1) Secondary transporters: glucosamine (GluA), galactosamine (GalA), and 5-dehydro-4-deoxy-glucosamine (5-dehydro-4-deoxy-GluA) are potentially imported using a single common transporter ExuT. Fucose (Fuc), rhamnose (Rha), and xylose (Xyl) are imported via dedicated proton symporters, while glucose (Glu), and galactose (Gal) are imported via dedicated sodium symporters. (2) ATP-binding cassette (ABC) transporters: ribose (Rib) and arabinose (Ara) sugars, as well as oligopeptides and dipeptides have dedicated ABC transporters with specific periplasmic substrate binding protein (SBP), two membrane permeases (P), and an ATPase. And (3) Phosphotransferase system (PTS) transporters: mannose (Man), fructose (Fru), galactosamine (GalN), and N-acetyl galactosamine (N-Ac-GalN) are imported via dedicated PTS transporters with cytoplasmic enzyme-I component (E-I) and membrane associated enzyme II components (IIA, IIB, and IIC). Sugars are phosphorylated during this kind of transport. The SAGs also encode a dedicated signal transduction system, and a tripartite ATP-independnent transporter (TRAP) for sensing, and importing, respectively, dicarboxylates, e.g. malate, and tricarboxylates, e.g. citrate, across the inner membrane. The signal transduction system is composed of the sensor histidine kinase DctB, and the cytoplasmic response regulator DctD, while the TRAP transporter is composed of the periplasmic solute receptor (DctP), the membrane small permease component (DctQ), and the membrane large permease component (DctM). TonB-dependent import of vitamin B12 and iron complexes is also predicted from the SAGs. Several proteins with Plug domains could potentially act as the outer membrane receptor protein for vitamin B12 and iron complexes. Binding of the ligand to the receptor activates TonB-dependent import across the outer membrane via three proteins TonB, ExbB, and ExbD, that couple proton motive force to ligand transport across the outer membrane. In the periplasm, vitamin B12 or iron complexes are then transported across the inner membrane via a dedicated ABC transporter.

Both S-E07 and S-B13 encode several non-specific porins for transport of substrates across the outer membrane, coupled to specialized transporters in the inner membrane, including multiple secondary (symport), ABC (ATP-binding cassette), and phosphotransferase system (PTS) transporters for the uptake of a wide array of monomers, e.g. those putatively produced from the degradation of all polymers described above (Fig 4, Table 2). Uronic acids and uronic acid derivatives are potentially imported using a single common transporter (a sugar phosphate permease transporter of the major facilitator superfamily similar to ExuT transporter of *Ralstonia solanacearum* [65]). Fucose, rhamnose, as well as xylose are potentially imported via dedicated proton symporters, while glucose and galactose are potentially imported via dedicated sodium symporters. Moreover, the SAGs encode components of dedicated ABC transporters for arabinose, ribose, and oligopeptides and dipeptides as well as components of the PTS specific for N-acetylgalactosamine, fructose, and mannose import. The SAGs also encode a complete two-component signal transduction system for sensing di/tricarboxylates, e.g. malate, citrate, (DctBD), as well as a tripartite ATP-independent di/tricarboxylate transport system (TRAP) (DctPQM) [66].

### Catabolism of imported sugars

Both S-E07 and S-B13 encode extensive pathways for the catabolism of a wide array of sugars, sugar acids, amino sugars, amino acids, as well as citrate and malate. Monomer degradation pathways in the SAGs are predicted to converge on one of three central metabolic routes, (i) feeding into the EMP pathway (for glucose, galactose, mannose, fructose, sugar acids, amino sugars, aspartate, and citrate and malate), (ii) feeding into PPP (for xylose, ribose, and arabinose), or (iii) the special fucose and rhamnose degradation pathways to propionate and propanol.

**Fig 5:**
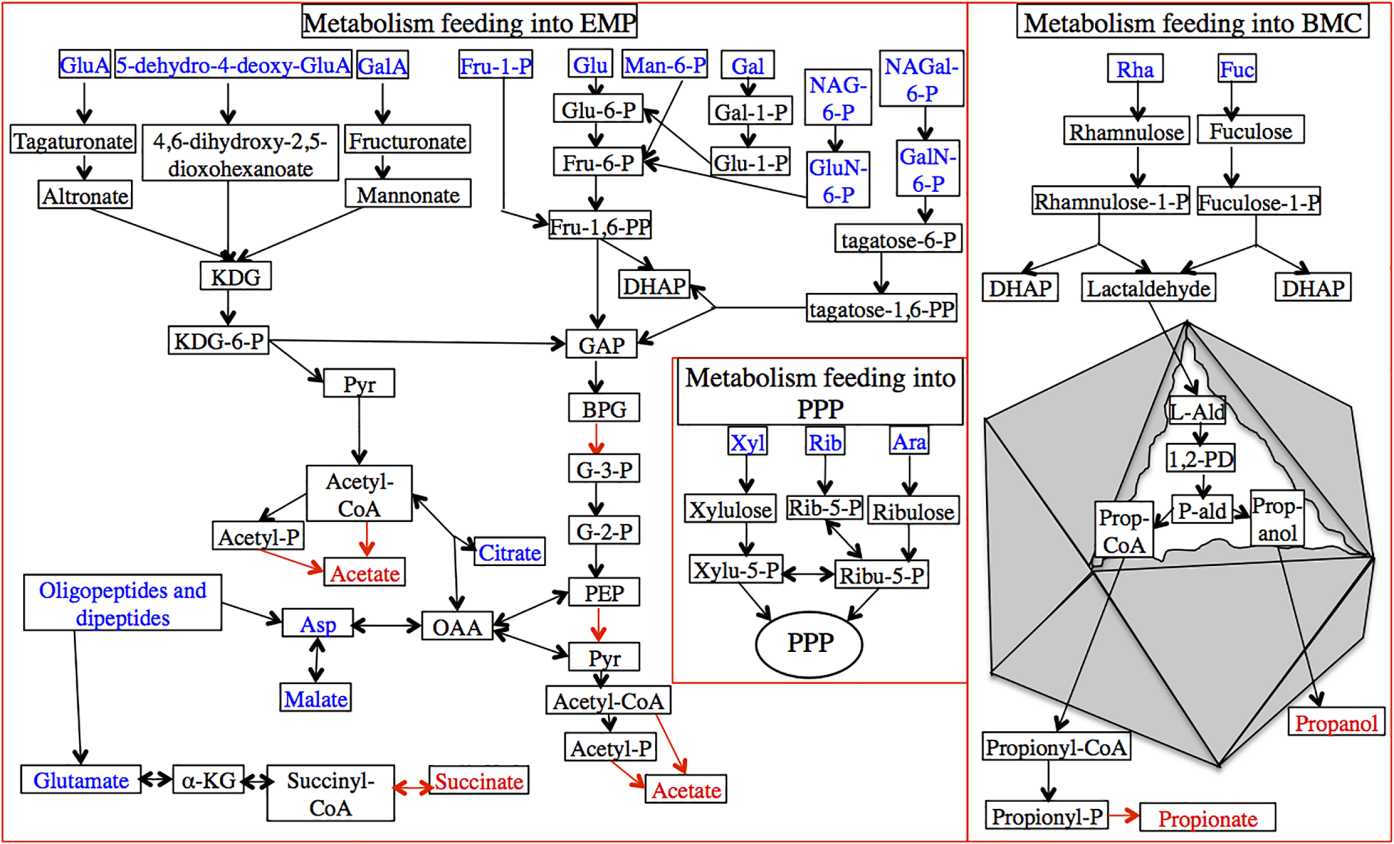
Metabolic reconstruction deduced from “Latescbacteria SAGs”. Metabolism is shown for the monomers produced during extracellular degradation of polymers (Table 2) followed by their transport across the outer and inner membranes as shown in Fig 3. Three major routes are shown (depicted by red boxes) for the degradation of those monomers, Embden-Meyerhof-Paranas (EMP) pathway, Pentose phosphate pathway (PPP), and bacterial microcompartment (BMC) pathway. The BMC is depicted by an octahedral structure showing all reactions thought to occur inside of the BMC. All possible substrates potentially supporting growth are shown in blue, predicted final products are shown in red, and reactions with substrate level phosphorylations are shown by red arrows. Abbreviations (other than those mentioned in Fig 3 legend): KDG, 2-dehydro-3-deoxy-D-gluconate; Pyr, pyruvate; Asp, aspartic acid; OAA, oxaloacetate; α-KG, α-ketoglutarate; Glu, glucose; Fru, fructose; Fru-1,6-PP, fructose-1,6-bisphosphate; DHAP, dihydroxyacetone phosphate; GAP, glyceraldehyde-3-phosphate; BPG, bisphosphoglycerate; G-3-P, 3-phosphoglycerate; G-2-P, 2-phosphoglycerate; PEP, phosphoenolpyruvate; Man, mannose; Gal, galactose; NAG, N-acetylglucosamine; NAGal, N-acetylgalactosamine; GluN, glucosamine; GalN, galactosamineRib, ribose; Ribu, ribulose; Xyl, xylose; Xylu, xylulose; Ara, arabinose; Rha, rhamnose; Fuc, fucose; L-Ald, lactaldehyde; 1,2-PD, 1,2-propanediol; P-ald, propionaldehyde; Prop-CoA, propionyl-CoA.

Monomer catabolism is detailed in the supplementary text and Fig 5. Briefly, the genomes encode a complete glycolytic pathway for metabolism of various C6 sugars to pyruvate, including glucose, galactose, mannose, and fructose and the amino sugars N-acetylgalactosamine, N-acetylglucosamine, and D-galactosamine. The genomes also encode the necessary enzymes for channeling the C6 sugar acids galacturonic acid, glucuronic acid, and 5-dehydro-4-deoxy-D-glucuronate to the central metabolite 2-dehydro-3-deoxy-D-gluconate (KDG), which is subsequently converted to pyruvate and glyceraldehyde-3-phosphate (GAP), that feed into the EMP. In addition, the amino acid aspartate, as well as dicarboxylates (malate) and tricarboxylates (citrate) that could potentially serve as C and energy source are catabolized via conversion to oxaloacetate and subsequently to phosphoenolpyruvate (PEP). On the other hand, the C5 sugars xylose, ribose, and arabinose are metabolized via the non-oxidative branch of the pentose phosphate pathway by first conversion to xylulose-5-P. Collectively, the metabolism of these compunds via the EMP or the PPP results in the production of pyruvate. Pyruvate could potentially be converted to acetyl-CoA via the action of pyruvate:ferredoxin oxidoreductase. Indeed, as indicated previously, the SAGs encode the machinery necessary for substrate-level phosphorylation including acetyl CoA synthase, as well as propanediol transacetylase and acetate kinase, both of which convert acetyl-CoA to acetate with concomitant ATP production (Fig 5).

Fucose and rhamnose metabolism requires a different catabolic pathway and partially occurs in an intracellular bacterial microcompartment (BMC) to protect against cellular damage by containing the reactive intermediate propionaldehyde [67, 68]. Both S-E07 and S-B13 encode a dedicated pathway for the degradation of fucose and rhamnose to lactaldehyde and dihydroxyacetone-phosphate. Several genes encoding for BMC structural shell proteins with BMC domains (pfam 00936, as well as pfam 03319) were identified in the SAGs consistent with a recent observation by Axen and colleagues exploring the taxonomic distribution of BMCs across bacterial phyla [69]. Inside the BMC, lactaldehyde is converted to 1,2-propanediol (1, 2-PD). Although homologues for 1,2-PD dehydratase, the enzyme responsible for conversion of 1,2-PD to propionaldehyde, were not identified in S-E07 and S-B13, both SAGs harbor NAD-dependent aldehyde dehydrogenase, and NADH-dependent alcohol dehydrogenase for conversion of propionaldehyde to propionyl-CoA, and propanol, respectively. Propionyl-CoA can then be converted to propionate with the concomitant production of 1 mole of ATP per propionate produced.

### Additional genomic features

Both S-E07 and S-B13 encode machinery for pili and flagella production, enabling potential attachment to surfaces [70], as well as gas vesicles production for maintaining a position in the water column with the most favorable growth conditions [71] (Supplementary Text). In addition, the SAGs encode multiple oxidative stress enzymes that counter harmful effects of changing oxygen tension caused by vertical migration in the stratified water column while in pursuit of decaying algal cells or other food particles. These include rubrerythrin, rubredoxin, rubredoxin oxidoreductase, superoxide reductase (desulfoferredoxin), ferritin-like protein, NADPH-dependent alkyl hydroperoxide reductase, and glutathione peroxidase [72], as well as machinery for bacillithiol biosynthesis, a thiol implicated in peroxide sensing [72–75].

### General features of Etoliko lagoon SAG E-K07

While the Etoliko lagoon SAG E-K07 shared similar metabolic potential with respect to algal cell wall polymer degradation to the Sakinaw Lake SAGs several unique features were apparent. In addition to harboring a large genome (estimated size 7.7 Mbp, Table 1) E-K07 encodes machinery for the following: (1) Degradation of the amino acids Thr, D-Cys, Glu, and Met, (2) Neuraminidase (GH33) gene for cleavage of sialic acid residues from N-linked glycoproteins, and endo-β-1,4-glucuronan lyase (PL20) [76], that targets β-(1→4)-glucuronan, a minor polysaccharide present in green algal cell walls [77], and (3) A papain (peptidase family C01), and a hyicolysin-like peptidase (family M30), possibly involved in matrix degradation. Also, E-K07 SAG encodes several stress response pathways, signal transduction, and defense mechanisms that were not identified in Sakinaw Lake SAGs. These include (1) oxidative stress enzymes catalase and ferroxidase, (2) CRISPR-associated genes including the 6 core *cas* genes (*cas1*-*cas6*), as well as the CRISPR-associated *csn1* gene [78], and (3) type VI secretion system including ten of the thirteen core *tss* genes [79].

## Discussion

Our analysis of four “*Latescibacteria*” SAGs obtained from the anaerobic monimolimnion water column of Sakinaw Lake, and the anaerobic sediments of Etoliko lagoon revealed extensive saccharolytic and proteolytic capabilities, with preference for specific polysaccharides and glycoproteins such as pectins, alginates, fucans, ulvans, xyloglucans, starch, extensins, and arabinogalactan protein originating from algal cell walls and EPS. While the degradation of some of these polymers (e.g. pectins and alginates) have been fairly well characterized at the genomic, enzymatic, and organismal levels [39, 40, 42], limited information is available regarding the pathways, genes, and microorganisms mediating the degradation of others (e.g. fucans, ulvans, extensins and arabinogalactan proteins) [44, 46, 47, 53, 54, 80]. More importantly, our knowledge of the degradation of many of these compounds is based on the study of model aerobic organisms with little knowledge of such pathways in anaerobes.

We argue that the observed patterns of polymer degradation, and monomer/oligomer transport and catabolism reflect niche specialization within *“Latescibacteria”* for survival and substrate acquisition in aquatic ecosystems. Specifically, we hypothesize that *“Latescibacteria”* SAGs analyzed in Sakinaw lake and Etoliko lagoon are involved in the degradation of a considerable fraction of algal cell wall polysaccharides and glycoprotein, algal EPS, and algal storage molecules within the detritus of green and brown algae originating at the oxic and photic zones and sinking to the anoxic and aphotic zones through sedimentation. Primary productivity is an important source for organic matter deposited in lakes [81, 82]. Algal cells represent up to 90% of such sinking organic matters, especially in stratified lakes like Sakinaw. Prior studies have demonstrated that CO_2_ fixation by algae represents the major source of organic carbon input in Sakinaw Lake, with the water column being the main site for the degradation of fixed organic carbon [83]. The stratified nature and lack of upwelling within meromictic lakes results in greater accumulation of organic matter into the lake’s deeper anoxic layers [84]. The overall contribution of algal detritus to lacustrine sediments is often enhanced by the frequent occurrence of algal blooms, an ecological phenomenon predicted to increase due to global warming trends, and the progressive increase in fertilizers usage [85]. This has been reported in the lagoon systems of Western Greece, where the occurrence of algal blooms and subsequent sedimentation of organic matter represent one of the driving forces for the observed progressive eutrophication and anoxia within this ecosystem [86, 87].

It should also be noted that, in addition to polymers putatively degraded “*Latescibacteria*”, algal cells are known to produce considerable quantities of oils (up to 60% of their weight), especially under unfavorable conditions (e.g. N and P starvation, temperature, salinity, or pH shifts, or heavy metal accumulation) [88, 89]. Interestingly, the analyzed SAGs lack all enzymes of the fatty acid degradation pathway to acetyl CoA. Similarly, cellulose represents an important constituent of green and brown algal cell wall [43], but the analyzed “*Latescibacteria*” SAGs display an extremely sparse cellulose degradation capacity (Figure A in S1 File). We reason that readily degradable components within algal detritus, e.g. cellular lipids and fatty acids, free proteins, and cellulose, are promptly utilized by microorganisms in the algal phycosphere [90–92], as well as by aerobic and anaerobic copiotrophs in the surrounding water column during the sedimentation process. Thus *“Latescibacteria”* residing in the deeper anaerobic layers of Sakinaw lake and Etoliko lagoon sediments have evolved to specialize in the degradation of the more recalcitrant substrates that accumulate as algal detritus descends to deeper anoxic layers in stratified aquatic ecosystems. Indeed, studies in meromictic lakes have demonstrated that degradation of algal blooms occurs during sedimentation leading to biomass loss and chemical structure alteration of the algal blooms with depth [81, 82].

The proposed ecological role for members of the “*Latescibacteria*” strongly suggests cellular attachment to sinking algal detritus. “*Latescibacteria*” SAGs encode genes for flagella and pili production, and formation of gas vesicles; traits that could enhance cellular capacity for tracking and attachment to particulate organic matter. A recent survey of microbial communities in the oxygen starved Black Sea with considerable primary productivity within the upper oxic zone, shows higher relative abundance of “*Latescibacteria*” in particulate-associated samples derived from the deep anoxic zone when compared to water samples from the same location [24].

In addition to the major contribution to sinking organic matter in water bodies, algal biomass degradation under anaerobic conditions has recently received additional attention due to its potential use for biogas production [93–99]. Surprisingly, little is currently known regarding the microbial community involved in algal biomass degradation under anaerobic conditions [93]. Thus analysis of *“Latescibacteria”* SAGs directly contributes to our understanding of potential bacterial lineages involved in the anaerobic turnover of algal cell components.

Finally, the *“Latescibacteria”* SAGs encode numerous biosynthetic capabilities and a rich repertoire of catabolic enzymes and transporters with the potential to promote growth on a large number of substrates. Such capabilities are in contrast to multiple recently obtained genomes of several uncultured bacterial and archaeal CP, where sparse anabolic capabilities, small genome size, and apparent dependence on syntrophic interactions for growth were observed [9, 13]. As such, the reported physiological properties (anaerobic nature and predicted slow growth rate due to possession of a relatively large genome size and a single rRNA operon), metabolic capabilities (distinct preference to specific polymers and sugars/sugar acids, auxotrophy to specific amino acids), and ecological distribution (preference to anaerobic and eutrophic habitats) should be considered when designing strategies for the isolation of members of the *“Latescibacteria”*.

## Supporting Information

S1 File Supporting Information document containing supplementary text, Tables A-C, and Figures A-B, accompany this manuscript. Table A. Genbank accession numbers, candidate order, and study site of all near-full-length 16S rRNA gene sequences affiliated with “*Latescibacteria*” that were used to construct phylogenetic trees shown in Fig 1. Table B. Total number of glycosyl hydrolases (GHs), polysaccharide lyases (PLs), and carbohydrate esterases (CEs) in the two most complete “*Latescibacteria*” SAGs compared to other lignocellulolytic and alginolytic organisms. Table C. Number of peptidases belonging to various Merops peptidase families identified in “*Latescibacteria*” genomes and their possible physiological roles. Figure A. Total number of “*Latescibacteria*” genes belonging to the different families of glycosyl hydrolases (GHs) and polysaccharide lyases (PLs) shown on the X-axis for SAGs S-E07 and S-B13. Figure B. Schematic representation of polymers shown in Table 2.
(DOCX)
