# Supplementary material for: In Silico Analysis of the Metabolic Potential and Niche Specialization of Candidate Phylum "Latescibacteria" (WS3)
Source: PLoS One. 2015 Jun 3;10(6):e0127499. doi: 10.1371/journal.pone.0127499 (PMC4454575; doi:10.1371/journal.pone.0127499)

**Supplementary Material for**

***In silico* analysis of the metabolic potential and niche specialization of candidate phylum “*Latescibacteria*” (WS3).**

Noha H. Youssef, Ibrahim F. Farag, Christian Rinke, Steven J. Hallam, Tanja Woyke, and Mostafa Elshahed

**Supplementary text:**

***I. Current taxonomic outline of the “Latescibacteria”.*** To identify all Sanger-generated near-full length “*Latescibacteria*” 16S rRNA gene sequences available in GenBank nr database (1), we performed BlastN (2) comparisons of all sequences classified as WS3 in Greengenes (3) against the Genbank nr database, using 75% sequence similarity cutoff. The identified sequences (n=9597) were filtered for chimera, length (> 900 bps), ambiguous nucleotides, and homopolymer stretches (<8) using MOTHUR (4). Filtered sequences were subjected to extensive phylogenetic analyses using MEGA6-Beta2 (5). This resulted in the identification of 1198 near-full length (>900bp) “*Latescibacteria*”-affiliated sequences, which were used to construct a reference phylogenetic tree for the “*Latescibacteria*” for determining the affiliation of identified SAGs.

***II. Detailed genomic analysis of “Latescibacteria”.*** This section describes a detailed analysis of S-E07 and S-B13 to reconstruct biosynthetic potential, export systems, information transfer machineries, central metabolic pathways, detailed sugar catabolic pathways, and detailed attachment and stress response strategies of “*Latescibacteria*”.

1. *Biosynthesis in “Latescibacteria”.*
   1. Amino acid biosynthesis: SAGs encode machinery responsible for the biosynthesis of most amino acids from glycolytic or TCA intermediates. Exceptions are alanine, proline, and branched chain amino acids, where genes encoding their biosynthesis are completely missing from the SAGs. Therefore it appears that “*Latescibacteria*” is auxotrophic for those amino acids. In support of this observation, SAGs encode several transporters for alanine, proline, and branched chain amino acids uptake. Also, only eight of the eleven genes for histidine biosynthesis are encoded by the SAGs. Hence, it is not clear if “*Latescibacteria*” is indeed auxotrophic for histidine or if those genes are missing due to the incomplete genomes. The SAGs also encode L-aspartate-alpha-decarboxylase responsible for conversion of aspartate to β-alanine, a precursor of pantothenate biosynthesis. The SAGs encode alanine racemase for conversion of L-ala to D-ala required for peptidoglycan biosynthesis.
   2. Purine and pyrimidine biosynthesis. The SAGs encode most of the purine biosynthetic machinery including all the genes necessary for conversion of glutamine and phosphoribosylpyrophosphate (PRPP) to aminoimidazolesuccinocarboxamide (AICAR) with the exception of phosphoribosylaminoimidazolesuccinocarboxamide synthase [EC. 6.3.2.6]. The SAGs also lack the 2 genes encoding phosphoribosylaminoimidazolecarboxamide formyltransferase and IMP cyclohydrolase for synthesis of inosine-monophosphate from AICAR. However, all genes for conversion of IMP to adenine and guanine and their deoxy derivatives were identified. In addition, the SAGs encode all genes necessary for pyrimidine biosynthesis with the exception of CTP synthase. It is worth noting that the above purine and pyrimidine biosynthesis genes that are missing from the Sakinaw Lake SAGs are encoded by the Etoliko lagoon SAG E-K07.
   3. Cofactor biosynthesis. While the SAGs encode enzymes for the conversion of thiamine to TPP, and riboflavin to FAD and FMN, they seem to be incapable of their biosynthesis thiamine, riboflavin. SAGs are also auxotrophic for vitamin B6, biotin, lipoic acid, and coenzyme A. On the other hand, “*Latescibacteria*” encodes machinery for the biosynthesis of nicotininc acid and nicotinamide, pantothenate, folate, and heme (including UroporphyrinogenI and III, Coproporphyrinogen I and III, protoporphyrinogen IX, and siroheme), and para-aminobenzoate.
   4. Fatty acid and phospholipid biosynthesis. Two of the fatty acid biosynthesis enzymes enoyl-ACP reductase, and fatty acyl-ACP hydrolase were not identified. The SAGs encode genes essential for biosynthesis of 1,2 diacyl-sn-glycerol-3-phosphate, as well as for the biosynthesis of the glycerophospholipids phosphatidyl-glycerol, phosphatidyl-serine, and phosphatidyl-ethanolamine.
   5. Terpenoid backbone biosynthesis. Terpenoid backbone is essential for the biosynthesis of the lipid carrier undecaprenyl-phosphate. The SAGs encode machinery for biosynthesis of 4-hydroxy-3-methylbut-2-en-1-yl diphosphate. However, IPP reductase which converts 4-hydroxy-3-methylbut-2-en-1-yl diphosphate to dimethylally-PP and isopentenyl-PP was not identified. The SAGs also lack the gene necessary for conversion of geranyl-PP to farnesyl-PP but possess all the downstream machinery for conversion of farnesyl-PP to undecaprenyl-PP.
   6. Nucleotide-sugar biosynthesis. The SAGs harbor evidence for biosynthesis of activated sugars including UDP-glucose, UDP-galactose, dTDP-rhamnose, GDP-mannose, GDP-fucose, and UDP-glucuronic acid. These nucleotide sugars are most probably involved in O-antigen biosynthesis as well as protein post-translational glycosylation.
   7. Cell membrane/cell wall biosynthesis and metabolism. Two peptidoglycan biosynthesis genes are missing from the Sakinaw lake SAGs including UDP-N-acetylglucosamine 1-carboxyvinyltransferase, and UDP-N-acetylmuramoyl-L-alanine:D-glutamate ligase. All other peptidoglycan genes are encoded by the SAGs. Given the data, “*Latescibacteria*” peptidoglycan lacks L-lys and has 2,6-diaminopimelate instead. Moreover, “*Latescibacteria*” appear to have a Gram-negative cell wall since machinery for lipopolysaccharide biosynthesis were identified including biosynthesis of KDO2-lipid A, the core oligosaccharide, and the lipidA-core. Glycosyl transferases (lipid carrier-phosphate-sugar-phospho transferases) that transfer glycosyl moities from NDP to the undecaprenyl-phosphate for O-antigen repeating unit assembly were also identified. While, a gene encoding the enzyme that transfers the repeating sugar unit to the growing O-antigen chain (O-antigen polymerase) was not identified, SAGs encode the enzyme that transfers the completed O-antigen from its lipid carrier to the core-lipid A (Lipid A core - O-antigen ligase). SAGs also encode MsbA, the lipid flippase that transports LPS from the inner leaflet to the outer leaflet of the cell membrane, and the machinery necessary for insertion of LPS in the outer membrane including LptBCDEF/G.
2. *Export systems in “Latescibacteria”*. The SAGs encode a complete Sec-independent Type I secretion system, including outer membrane protein TolC, membrane fusion protein (MFP), and an ATPase/permease transporter. Type I SS are known to export proteins from the cytoplasm to the extracellular milieu bypassing the periplasm. The SAGs also encode β-barrel assembly (BAM) complex for insertion of protein in the outer membrane. This includes periplasmic chaperones (Skp, and SurA) to keep the proteins in the folded state, and the BAM complex proteins BamA, and BamD. The genomes also encode complete Sec-dependent (SecA, SecB, SecD/F, SecYEG, YajC, YidC, FtsY, and signal recognition particle SRP) and Tat-dependent (TatA/E, TatC, and TatD) transport machineries for the transloaction of proteins across the inner membrane.
3. *Information transfer machines.*
   1. *DNA replication.* SAGs encode some of the DNA replication machinery including two subunits of DNA polymerase III (α, and γ/τ), replicative DNA helicase DnaB, DNA primase DnaG, single-stranded DNA-binding proteins, RNaseH, DNA polymerase I, and DNA ligase.
   2. *Transcription.* SAGs encode only three subunits of DNA-directed RNA polymerase, α, β, and β’. Sigma factors belonging to families Sigma-70/32, Sigma-54, Sigma-24 were identified in the SAGs, as were transcription elongation factors NusA and GreA. Several transcriptional regulators are present in the genome belonging to families AlpA, XRE, AsnC, ArsR, DeoR, GntR, TetR, TraR/DksA, NrdR, and CPR/FNR.
   3. *Translation.* Most of the small subunit (17 out of 20) and the large subunit (27 out of 35) ribosomal proteins were identified. SAGs also encode at least one tRNA for all amino acids except phenylalanine and tyrosine. Amino acyl-tRNA synthetases are encoded for all amino acids except phenylalanine and tyrosine. Translation initiation factors IF-1, IF-2, and IF-3, and translation elongation factors EF-Tu, and EF-G were also identified. Other translation-associated factors include ribosome-binding factor A, peptide chain release factors 1 and 2, methionine aminopeptidase type I, tRNA nucleotidyltransferase (CCA adding enzyme), as well as N-formylmethionyl-tRNA deformylase and formyltransferase.
4. *Central metabolic pathways*
   1. *Glycolysis/ gluconeogenesis.* The SAGs encode a complete glycolytic pathway including the three irreversible enzymes glucokinase, phosphofructokinase, and pyruvate kinase. Gluconeogenic irreversible enzyme pyruvate:phosphate dikinase was also identified. While the SAGs lack a fructose-1,6-bisphosphatase, a copy of archaeal inositol monophosphatase, shown to have a dual activity as inositol-1(4)-monophosphatase, and fructose-1,6-bisphosphatase, was identified with the potential to serve a gluconeogenic role in “*Latescibacteria”*.
   2. *Pentose phosphate pathway.* The SAGs encode all the enzymes of the reductive branch of the pentose phosphate pathway. This branch is essential for the conversion of 6-carbon sugars to the 5-carbon sugars ribose and deoxyribose (essential for purine and pyrimidine biosynthesis), and to phosphoribosyl-pyrophosphate (essential for histidine biosynthesis). On the other hand, no enzymes for the oxidative branch of the pathway (glucose-6-phosphate dehydrogenase, 6-phosphogluconolactonase, or 6-phosphogluconate dehydrogenase) were identified.
   3. *Tricarboxylic acid cycle.* TCA cycle in “*Latescibacteria*” seems to be mainly anapleurotic, providing biosynthetic intermediates (e.g α-ketoglutarate, and succinyl-CoA) for biosynthesis, as well as allowing the use of dicarboxylic acids (e.g. malate) and tricarboxylic acids (e.g. citrate) as potential energy and C sources (see below and main text). TCA cycle enzymes encoded by the SAGs include citrate synthase, α-ketoacid:ferredoxin oxidoreductase, succinyl-CoA ligase, and malate dehydrogenase.
5. *Dedicated sugar catabolic pathways.*

In addition to the extensive polymer degradation and transport machinery identified, the SAGs encode extensive pathways for the catabolism of a wide array of sugars, sugar acids, amino sugars, a few amino acids, as well as citrate and malate putatively imported by the *Latescibacteria*. All monomer degradation pathways converge on one of three central metabolic routes, (i) feeding into the EMP pathway (for glucose, galactose, mannose, fructose, sugar acids, amino sugars, glutamate and aspartate, and citrate and malate), (ii) feeding into PPP (for xylose, ribose, and arabinose), or (iii) the special fucose and rhamnose degradation pathways to propionate and propanol. Monomer catabolism is detailed below.

*a. Monomers feeding into EMP.* The SAGs encode a complete glycolytic pathway (see above) for metabolism of various C6 sugars to pyruvate, including glucose (after activation to glu-6-P), galactose (after conversion to glu-1-P via the LeLoir pathway), mannose (after conversion to fructose-6-P via mannose-6-P isomerase), and fructose (after conversion to fructose-1,6-bisphosphate via phosphofructokinase). The SAGs also encode enzymes for channeling the C6 sugar acids galacturonic acid, glucuronic acid, and 5-dehydro-4-deoxy-D-glucuronate to the central metabolite 2-dehydro-3-deoxy-D-gluconate (KDG). Briefly, galcaturonic acid, and glucuronic acid are potentially converted to fructuronate, and tagaturonate, respectively, by the action of glucuronate isomerase (EC. 5.3.1.12). Mannitol-1-P/ altronate NAD-dependent dehydrogenase then converts fructuronate, and tagaturonate to mannonate, and altronate, respectively. Mannonate dehydratase, and altronate dehydratase then convert mannonate, and altronate, respectively, to KDG. As for 5-dehydro-4-deoxy-D-glucuronate, it is potentially converted to KDG by the consecutive actions of an isomerase and a dehydrogenase. While a specific 5-dehydro-4-deoxy-D-glucuronate isomerase, e.g. EC. 5.3.1.17, was not identified, a specific dehydrogenase (EC. 1.1.1.125) is present with the potential to fill this role. Following conversion to KDG, the consecutive action of KDG kinase, and KDGP aldolase then converts KDG to pyruvate and glyceraldehyde-3-phosphate (GAP), which feed into the EMP*.* The SAGs also have the potential to catabolize C6 aminosugars. N-acetylgalactosamine (NAGal), N-acetylglucosamine (NAG), and D-galactosamine (GalN), are modified during PTS import to NAGal-6-P, NAG-6-P, and GalN-6-P, respectively. N-acetylglucosamine-6-P deacetylase converts NAGal-6-P to GalN-6-P, and NAG-6-P to GluN-6-P. Glucosamine-6-P isomerase/deaminase converts GluN-6-P to fructose-6-P, which feeds into EMP, and GalN-6-P to tagatose-6-P. Phosphofructokinase then coverts tagatose-6-P to tagatose-1,6-bisphosphate, which is broken down by the action of fructose-1,6-bisphosphate aldolase to GAP and dihydroxyacetone phosphate (DHAP), both of which feed into the EMP. Aspartate could potentially serve as C and energy source. The SAGs encodes an aspartate aminotransferase, which convert Asp to oxaloacetate (OAA). OAA could potentially be converted to phosphoenolpyruvate (PEP) via PEP carboxykinase, or to pyruvate via OAA decarboxylase. The potential for using dicarboxylates and tricarboxylates as potential C and energy sources was also identified. The presence of a citrate synthase with the absence of other essential TCA cycle enzymes (aconitase, isocitrate dehydrogenase) might suggest that citrate synthase is functioning in the reverse direction converting citrate to OAA and acetyl-CoA. The SAGs also encode a malate dehydrogenase that would convert malate to OAA. Oxaloacetate could potentially be converted to PEP or pyruvate as described above.

*b. Monomers feeding into PPP.* On the other hand, the C5 sugars xylose and arabinose are metabolized via the pentose phosphate pathway. The SAGs encodes an arabinose isomerase that converts arabinose to ribulose, and a xylose isomerase that converts xylose to xylulose. Xylulokinase converts ribulose, and xylulose to ribulose-5-P (Ribu-5-P), and xylulose-5-P, respectively. Ribu-5-P-3-epimerase converts Ribu-5-P to xylulose-5-P, which feeds into the PPP.

Collectively, the metabolism of the above sugars, sugar acids, and amino sugars results in the production of pyruvate via EMP. Pyruvate could potentially be converted to acetyl-CoA via the action of pyruvate:ferredoxin oxidoreductase. The SAGs encode acetyl CoA synthase, as well as propanediol transacetylase and acetate kinase, both of which convert acetyl-CoA to acetate with concomitant ATP production.

*c. Fucose and rhamnose metabolism to propanol and propionate.* Fucose and rhamnose metabolism requires a different pathway and partially occurs in an intracellular bacterial microcompartment (BMC) to protect against cellular damage by containing the reactive metabolite intermediate propionaldehyde (6, 7)*.* The SAGS encode a dedicated pathway for the degradation of both fucose and rhamnose. Fucose isomerase, and xylose isomerase convert fucose, and rhamnose to fuculose, and rhamnulose, respectively. Sugar (pentulose and hexulose) kinases convert fuculose, and, rhamnulose to fuculose-1-P, and rhamnulose-1-P, respectively. Fuculose-1-P aldolase then converts fuculose-1-P, and rhamnulose-1-P to lactaldehyde and DHAP. DHAP feeds into EMP, while lactaldehyde is converted to 1,2-propanediol (1, 2-PD) via the action of alcohol dehydrogenase (propanol preferring). Genomic evidence suggests possession of an intracellular bacterial microcompartment (BMC). The SAGs encode BMC structural shell proteins with BMC domains (pfam 00936, as well as pfam 03319), indicating that *Latescibacteria* have the potential to construct intracellular BMC structures for 1,2-PD conversion. Inside the BMC, 1,2-PD is converted to propionaldehyde via 1,2-propanediol dehydratase. Two distinct 1,2-propanediol dehydratases were identified; a B12-dependent diol dehydratase, which employes a B12 cofactor and requires an activating enzyme of the HSP70-ATPase class (8); and a B12-independent diol dehydratase requiring an activating enzyme of the PFL-activase family of enzymes (9, 10). Homologues of neither the B12-dependent, nor the B12-independent diol dehydratases were identified. However, SAGs encode homologues of both families of the activating enzymes (PFL-activase family, and HSP70 ATPase domain-containing activase enzyme). The next step in 1,2-PD degradation is the NAD-dependent dehydrogenation of propionaldehyde to propionyl-CoA. The SAGs encode a NAD-dependent aldehyde dehydrogenase potentially catalyzing this reaction. NADH-dependent alcohol dehydrogenase also converts propionaldehyde to propanol, potentially recycling NAD to the oxidized form. Propionyl-CoA can then be converted to propionate via the consecutive action of propanediol transacetylase and acetate kinase with the concomitant production of 1 mole of ATP per propionate produced.

*F. Detailed attachment and stress response strategies.*

The above catabolic capabilities strongly suggest that polymeric polysaccharides and glycoproteins of mainly algal origin represent an important nutrition source for “*Latescibacteria”*. SAGs also harbor evidence for pili production, and possession of flagella that could potentially enable surface attachment (11), gas vesicles production that could potentially help maintain a position in the water column that provides the most favorable growth conditions including the availability of food sources (12), as well as oxidative stress enzymes to overcome the changing O_2_ tension that might be encountered as a consequence of moving through the water column in pursuit of decaying algal cells or other food particles. These mechanisms are described below.

*a. Type IV Pili (TFP) production.* The SAGS encode for type IV pilin major subunit (PilA) and the prepilin peptidase (Merops family A24) responsible for the removal of N-terminal type III leader sequence and transferring a methyl group to the new N-terminus, both essential for PilA activation. An outer membrane secretin essential for export of pilus across the outer membrane was also identified. The SAGS also encode inner membrane complex components PilMNO, as well as the traffic ATPase PilF are essential for pilus assembly as well as a gene encoding for PilT, a retraction ATPase essential for pilus depolymerization (13). Minor pilins were not identified in the SAGs and might be dispensible for pilus assembly and biogenesis as shown before for some G^-^ bacteria, e.g. *Neisseria* sp (14).

*b. Possession of flagella.* The SAGs encode most of the 24-core set of genes previously suggested to constitute the structural components of the flagellum (15). With the exception of the filament FliC and the hook-filament junction proteins FlgK and FlgL, SAGs encode the hook protein FlgE, hook capping protein FlgD, proximal rod proteins FliE, FlgB, and FlgC, outer membrane L and P ring proteins FlgH and FlgI, MS-basal body ring protein FliF, C-ring protein FliN, FliM, FliG, motor proteins MotAB, export proteins FlhAB, FliI, FliP, FliQ, FliR, FliH, FliO, hook length control protein FliK. The lack of the filament and hook-filament junction proteins in the presence of all other structural assembly components could possibly be explained by incompleteness of the SAGs.

*c. Gas vesicles.* Gas vesicles are intracellular gas-filled protein structures common to prokaryotic microorganisms inhabiting aquatic habitats, especially those inhabiting the anaerobic layers of stratified lakes. They provide buoyancy to the cells by decreasing their cell density. The SAGs encode a *gvp* cluster with all essential genes for gas vesicle production including GvpA, the gas vesicle structural protein, the ATPase chaperone GvpN, GvpO with an essential but unknown function, and GvpFGKLM, which could function as scaffolding protein (16).

1. *Oxidative stress enzymes.* The SAGs encode several genes involved in oxidative stress. These include rubrerythrin, rubredoxin, rubredoxin oxireductase, superoxide reductase (desulfoferredoxin), ferritin-like protein, NADPH-dependent alkyl hydroperoxide reductase, and glutathione peroxidase (17). All the genes essential for bacillithiol biosynthesis (N-acetyl-alpha-D-glucosaminyl L-malate synthase BshA, bacillithiol biosynthesis deacetylase BshB1, and bacillithiol biosynthesis cysteine-adding enzyme BshC) were identified. Bacillithiol is a low molecular weight thiol (LMW) produced by Firmicutes and other Gram-positive bacteria to protect against disulfide bond formation under oxidative stress (18, 19). Bacillithiol is the Gram-positive alternative to glutathione, the major LMW thiol of Gram-negative bacteria and eukaryotes (20). Genes necessary for glutathione biosynthesis were not identified in the SAGs. Interestingly, genes for bacillithiol biosynthesis have been identified in only a few Gram-negative bacteria including *Myxococcus xanthus*, and *Natranaerobius thermophilus* (21).

***III. Structural description and occurrences of algal cell wall polymers.*** Below, we provide a detailed description of various algal cell wall polymer structures (Fig. S2). Distribution and occurrences among various algal taxa are also provided.

1. Pectin. Pectins are widely distributed in nature as major components of the primary cell wall of non-woody terrestrial plants (22). They, together with hemicelluloses, constitute the matrix in which cellulose microfibrils are embedded (23). Pectins are also abundant in green algal cell walls of the order Charophyta, especially in the middle amorphous layer and the outer lattice (24). Pectin occurs either as an unsubstituted homogalacturonan with a linear backbone of α-1,4-linked D-galacturonic acid residues, and where the carboxyl groups of the galacturonate units are often methylesterified and/or acetylated, or as a substituted xylogalacturonan (homogalacturonan backbone branched by β-1,3-linked D-xylose), or rhamnogalacturonan I where some D-galacturonate units in the homogalacturonan backbone are replaced by α-1,2-linked L-rhamnose, to which side chains of D-galactose, L-arabinose and α-D-xylopyranose branch off (23). Green algae of the order Charophyta have both homogalacturonan and rhamnogalacturonan I in their cell wall middle amorphous layer, and mainly homogalacturonan in the outer cell wall lattice (24).
2. Alginate. Alginate is the major matrix component of the brown algal cell wall, which may constitute up to 45% of the dry weight of the alga (25). Alginate is present in brown algae within the fibrillar cell wall layers enmeshing cellulose microfibrils, and also in the inter-fibrillar layers (26). Alginate is a linear co-polymer of 1,4-linked β-D-mannuronate, with variable amounts of its C-5 epimer α-L-guluronate (26).
3. Fucans. Fucans are present together with alginates in the inter-fibrillar layers of brown algal cell walls. They constitute up to 20% of the dry cell wall weight (25), and are tightly associated with proteins and cellulose, where they might have a role in adaptation to osmotic stress (26). Fucans exhibit wide variations in chemical structures, ranging from the highly sulfated homofucan polymers, to the highly branched high-uronic-acid, low-sulfate-containing polymers (xylofucoglucan, xylofucoglucuronan) (25). Homofucans backbone consists of α-linked fucose sulfated at the C4 or C2 position. Fucans also exist in highly substituted forms. These include xylofucoglucan: polymer of 1,4-linked β-D-glucose with substitutions at the O-6 position with mono, di, or triglycosyl residues of α-linked xylose, β-linked galactose, and α-linked fucose; xylofucoglucuronan: polymer of 1,4-linked β-D-glucuronic acid substituted with α-linked xylose, and α-linked fucose (27).
4. Ulvans. Ulvans are integral components of the green algal cell walls of the order Chlorophyta, esp. *Ulva* species, where they represent 8-30% of the cell wall dry weight. While the exact chemical composition of ulvans is not fully characterized, there is evidence that the repeating unit in ulvan backbone is mixture of 3 disaccharides: 3-sulfated rhamnose (Rha3S) linked to glucuronic acid, Rha3S lnked to iduronic acid, or Rha3S linked to xylose (28). Recent studies on a marine Bacteroidetes species, Nonlabens ulvanivorans, identified the main ulvan-degrading enzyme to be ulvan lyase, capable of cleaving the glycosidic bond between Rha3S and one of the uronic acid residues to give rise to oligosaccharides with an unsaturated uronic acid at the non-reducing end (28). No similarities to other polysaccharide lyases in the databases were detected for the purified ulvan lyase. Previous studies on an unidentified marine Gram-negative bacterium indicated the presence of an endo-ulvan lyase activity (29). While the purified enzyme was not sequenced, the product pattern identified by H-NMR and C-NMR indicated an enzyme with different activity than the ulvan lyase purified from Nonlabens ulvanivorans. This suggests an apparent variation between ulvan lyases from different species.
5. Xyloglucan. While xyloglucan is the major hemicellulose in non-gramineous plants and was shown to be present in all vascular plants, it only constitutes a minor component of green algal cell walls, esp. *Ulva* sp. in order Chlorophyta, and some Charophyta green algae (30, 31). In green plants, xyloglucan has a backbone of β-1,4-linked cellotetraose units, which are substituted in C-6 by xylosyl residues at two or three of the first four glucosyl residues (but not the residue closest to the reducing end). Some of the xylosyl residues are subsequently further substituted by (1,2)-β-D-galactosyl residues at the second and/or third xylose residue and by (1,2)-α-L-fucose at the galactosyl unit of the third xylose residue (32). Previous research suggested that the linkage of xylose residues to the xyloglucan backbone differs between green plants and algae, where it is α-1,6 in green plants and β-linked in algae (33).
6. Hydroxyproline-rich glycoprotein. In addition to polysaccharides, algal cell walls also contain glycoproteins. Indeed, proteins can account to 20-40% of the cell wall dry weight in some instances (34-36). Hydroxyproline-rich glycoproteins (HRGPs) have been found in the insoluble cell wall fraction of green algae (36). Hydroxyproline-rich glycoproteins similar to those of extensins and arabinogalactan proteins (AGPs) from land plants were detected in green algal cell walls (31). Extensins are HRGPs where hydroxyproline residues are glycosylated with short chains of L-arabinose. Recently, it was shown that glycosyl hydrolases belonging to the family GH127 with β-L-arabinofuranosidase activity specifically target arabinose residues attached to hydroxyproline in extensins. Arabinogalactans proteins (AGP) are HRGPs present in the cell walls of brown and green algae. The glycan moiety of AGPs accounts for >90% of its mass (37). The basic structure of the glycan moiety is a backbone of β-1,3-galactan, branched with side chains of β-1,6-galactan. Galactosyl residues are further substituted with arabinose, and less frequently also with fucose, rhamnose, and (methyl) glucuronic acid (37).

**References.**

1. 1. **Benson DA, Clark K, Karsch-Mizrachi I, Lipman DJ, Ostell J, Sayers EW.** 2014. GenBank. Nucleic Acids Res **42:**D32-37.
2. 2. **Johnson M, Zaretskaya I, Raytselis Y, Merezhuk Y, McGinnis S, Madden TL.** 2008. NCBI BLAST: a better web interface. Nucleic Acids Res **36:**W5-9.
3. 3. **McDonald D, Price MN, Goodrich J, Nawrocki EP, DeSantis TZ, Probst A, Andersen GL, Knight R, Hugenholtz P.** 2012. An improved Greengenes taxonomy with explicit ranks for ecological and evolutionary analyses of bacteria and archaea. ISME J **6:**610-618.
4. 4. **Schloss PD, Westcott SL, Ryabin T, Hall JR, Hartmann M, Hollister EB, Lesniewski RA, Oakley BB, Parks DH, Robinson CJ, Sahl JW, Stres B, Thallinger GG, Van Horn DJ, Weber CF.** 2009. Introducing mothur: open-source, platform-independent, community-supported software for describing and comparing microbial communities. Appl Environ Microbiol **75:**7537-7541.
5. 5. **Tamura K, Stecher G, Peterson D, Filipski A, Kumar S.** 2013. MEGA6: Molecular Evolutionary Genetics Analysis version 6.0. Mol Biol Evol **30:**2725-2729.
6. 6. **Havemann GD, Bobik TA.** 2003. Protein content of polyhedral organelles involved in coenzyme B12-dependent degradation of 1,2-propanediol in *Salmonella enterica* serovar *Typhimurium* LT2. J Bacteriol **185:**5086-5095.
7. 7. **Petit E, LaTouf WG, Coppi MV, Warnick TA, Currie D, Romashko I, Deshpande S, Haas K, Alvelo-Maurosa JG, Wardman C, Schnell DJ, Leschine SB, Blanchard JL.** 2013. Involvement of a bacterial microcompartment in the metabolism of fucose and rhamnose by *Clostridium phytofermentans*. PLoS ONE **8:**e54337.
8. 8. **Seifert C, Bowien S, Gottschalk G, Daniel R.** 2001. Identification and expression of the genes and purification and characterization of the gene products involved in reactivation of coenzyme B12-dependent glycerol dehydratase of *Citrobacter freundii*. Eur J Biochem **268:**2369-2378.
9. 9. **O'Brien JR, Raynaud C, Croux C, Girbal L, Soucaille P, Lanzilotta WN.** 2004. Insight into the mechanism of the B12-independent glycerol dehydratase from *Clostridium butyricum*: preliminary biochemical and structural characterization. Biochemistry **43:**4635-4645.
10. 10. **Raynaud C, Sarcabal P, Meynial-Salles I, Croux C, Soucaille P.** 2003. Molecular characterization of the 1,3-propanediol (1,3-PD) operon of *Clostridium butyricum*. Proc Natl Acad Sci USA **100:**5010-5015.
11. 11. **Dunne WM.** 2002. Bacterial Adhesion: Seen Any Good Biofilms Lately? Clin Microbiol Rev **15:**155-166.
12. 12. **Walsby AE.** 1994. Gas vesicles. Microbiol Rev **58:**94-144.
13. 13. **Takhar HK, Kemp K, Kim M, Howell PL, Burrows LL.** 2013. The platform protein is essential for type IV pilus biogenesis. J Biol Chem **288:**9721-9728.
14. 14. **Winther-Larsen HC, Hegge FT, Wolfgang M, Hayes SF, van Putten JP, Koomey M.** 2001. *Neisseria gonorrhoeae* PilV, a type IV pilus-associated protein essential to human epithelial cell adherence. Proc Natl Acad Sci USA **98:**15276-15281.
15. 15. **Liu R, Ochman H.** 2007. Stepwise formation of the bacterial flagellar system. Proc Natl Acad Sci USA **104:**7116-7121.
16. 16. **Pfeifer F.** 2012. Distribution, formation and regulation of gas vesicles. Nat Rev Microbiol **10:**705-715.
17. 17. **Briukhanov AL, Netrusov AI.** 2007. Aerotolerance of strictly anaerobic microorganisms and factors of defense against oxidative stress: a review. Prikl Biokhim Mikrobiol **43:**635-652.
18. 18. **Posada AC, Kolar SL, Dusi RG, Francois P, Roberts AA, Hamilton CJ, Liu GY, Cheung A.** 2014. Importance of bacillithiol in the oxidative stress response of *Staphylococcus aureus*. Infect Immun **82:**316-332.
19. 19. **Newton GL, Rawat M, La Clair JJ, Jothivasan VK, Budiarto T, Hamilton CJ, Claiborne A, Helmann JD, Fahey RC.** 2009. Bacillithiol is an antioxidant thiol produced in Bacilli. Nat Chem Biol **5:**625-627.
20. 20. **Fahey RC, Brown WC, Adams WB, Worsham MB.** 1978. Occurrence of glutathione in bacteria. J Bacteriol **133:**1126-1129.
21. 21. **Gaballa A, Newton GL, Antelmann H, Parsonage D, Upton H, Rawat M, Claiborne A, Fahey RC, Helmann JD.** 2010. Biosynthesis and functions of bacillithiol, a major low-molecular-weight thiol in Bacilli. Proc Natl Acad Sci USA **107:**6482-6486.
22. 22. **Voragen AJ, Coenen G-J, Verhoef R, Schols H.** 2009. Pectin, a versatile polysaccharide present in plant cell walls. Struct Chem **20:**263-275.
23. 23. **Harholt J, Suttangkakul A, Vibe Scheller H.** 2010. Biosynthesis of pectin. Plant Physiol **153:**384-395.
24. 24. **Domozych DS, Sorensen I, Popper ZA, Ochs J, Andreas A, Fangel JU, Pielach A, Sacks C, Brechka H, Ruisi-Besares P, Willats WG, Rose JK.** 2014. Pectin metabolism and assembly in the cell wall of the charophyte green alga *Penium margaritaceum*. Plant Physiol **165:**105-118.
25. 25. **Michel G, Tonon T, Scornet D, Cock JM, Kloareg B.** 2010. The cell wall polysaccharide metabolism of the brown alga *Ectocarpus siliculosus*. Insights into the evolution of extracellular matrix polysaccharides in Eukaryotes. New Phytol **188:**82-97.
26. 26. **Deniaud-Bouët E, Kervarec N, Michel G, Tonon T, Kloareg B, Hervé C.** 2014. Chemical and enzymatic fractionation of cell walls from Fucales: insights into the structure of the extracellular matrix of brown algae. Ann Bot **114:**1203-1216.
27. 27. **Ale MT, Mikkelsen JD, Meyer AS.** 2011. Important determinants for fucoidan bioactivity: a critical review of structure-function relations and extraction methods for fucose-containing sulfated polysaccharides from brown seaweeds. Mar Drugs **9:**2106-2130.
28. 28. **Nyvall Collen P, Sassi JF, Rogniaux H, Marfaing H, Helbert W.** 2011. Ulvan lyases isolated from the Flavobacteria *Persicivirga ulvanivorans* are the first members of a new polysaccharide lyase family. J Biol Chem **286:**42063-42071.
29. 29. **Lahaye M, Brunel M, Bonnin E.** 1997. Fine chemical structure analysis of oligosaccharides produced by an ulvan-lyase degradation of the water-soluble cell-wall polysaccharides from *Ulva* sp. (Ulvales, Chlorophyta). Carbohydr Res **304:**325-333.
30. 30. **Bobin-Dubigeon C, Lahaye M, Guillon F, Barry J-L, Gallant DJ.** 1997. Factors limiting the biodegradation of Ulva sp cell-wall polysaccharides. J Sci Food Agri **75:**341-351.
31. 31. **Domozych DS, Ciancia M, Fangel JU, Mikkelsen MD, Ulvskov P, Willats WG.** 2012. The cell walls of green algae: a journey through evolution and diversity. Front Plant Sci **3:**82.
32. 32. **Hsieh YSY, Harris PJ.** 2009. Xyloglucans of monocotyledons have diverse structures. Mol Plant **2:**943-965.
33. 33. **Lahaye M, Jegou D, Buleon A.** 1994. Chemical characteristics of insoluble glucans from the cell wall of the marine green alga *Ulva lactuca* (L.) Thuret. Carbohydr Res **262:**115-125.
34. 34. **Fleurence J.** 1999. The enzymatic degradation of algal cell walls: a useful approach for improving protein accessibility? J Appl Phycol **11:**313-314.
35. 35. **Burczyk J, Śmietana B, Termińska-Pabis K, Zych M, Kowalowski P.** 1999. Comparison of nitrogen content amino acid composition and glucosamine content of cell walls of various chlorococcalean algae. Phytochemistry **51:**491-497.
36. 36. **Voigt J, Stolarczyk A, Zych M, Malec P, Burczyk J.** 2014. The cell-wall glycoproteins of the green alga *Scenedesmus obliquus*. The predominant cell-wall polypeptide of *Scenedesmus obliquus* is related to the cell-wall glycoprotein gp3 of *Chlamydomonas reinhardtii*. Plant Sci **215–216:**39-47.
37. 37. **Knoch E, Dilokpimol A, Geshi N.** 2014. Arabinogalactan proteins: focus on carbohydrate active enzymes. Front Plant Sci **5:**198.
38. 38. **Kormas KA, Meziti A, Dahlmann A, GJ DEL, Lykousis V.** 2008. Characterization of methanogenic and prokaryotic assemblages based on mcrA and 16S rRNA gene diversity in sediments of the Kazan mud volcano (Mediterranean Sea). Geobiology **6:**450-460.
39. 39. **Lin X, Kennedy D, Fredrickson J, Bjornstad B, Konopka A.** 2012. Vertical stratification of subsurface microbial community composition across geological formations at the Hanford Site. Environ Microbiol **14:**414-425.
40. 40. **Youssef N, Sheik CS, Krumholz LR, Najar FZ, Roe BA, Elshahed MS.** 2009. Comparison of species richness estimates obtained using nearly complete fragments and simulated pyrosequencing-generated fragments in 16S rRNA gene-based environmental surveys. Appl Environ Microbiol **75:**5227-5236.
41. 41. **Beal EJ, House CH, Orphan VJ.** 2009. Manganese- and iron-dependent marine methane oxidation. Science **325:**184-187.
42. 42. **D'Auria G, Baron-Rodriguez M, Durban-Vicente A, Moya A, Rojo C, Latorre A, Rodrigo M.** 2010. Unravelling the bacterial diversity found in the semi-arid Tablas de Daimiel National Park wetland (central Spain). Aquat Microb Ecol **59:**33-44.
43. 43. **Derakshani M, Lukow T, Liesack W.** 2001. Novel bacterial lineages at the (sub)division level as detected by signature nucleotide-targeted recovery of 16S rRNA genes from bulk soil and rice roots of flooded rice microcosms. Appl Environ Microbiol **67:**623-631.
44. 44. **Forget NL, Murdock SA, Juniper SK.** 2010. Bacterial diversity in Fe-rich hydrothermal sediments at two South Tonga Arc submarine volcanoes. Geobiology **8:**417-432.
45. 45. **Freitag TE, Prosser JI.** 2003. Community structure of ammonia-oxidizing bacteria within anoxic marine sediments. Appl Environ Microbiol **69:**1359-1371.
46. 46. **Fuchsman CA, Kirkpatrick JB, Brazelton WJ, Murray JW, Staley JT.** 2011. Metabolic strategies of free-living and aggregate-associated bacterial communities inferred from biologic and chemical profiles in the Black Sea suboxic zone. FEMS Microbiol Ecol **78:**586-603.
47. 47. **Green-Garcia AM.** 2007. Characterization of the lucinid bivalve-bacteria symbiotic system: the significance of the geochemical habitat on bacterial symbiont diversity and phylogeny. Master of Science (M.S.). Louisiana State University.
48. 48. **Handley KM, VerBerkmoes NC, Steefel CI, Williams KH, Sharon I, Miller CS, Frischkorn KR, Chourey K, Thomas BC, Shah MB, Long PE, Hettich RL, Banfield JF.** 2013. Biostimulation induces syntrophic interactions that impact C, S and N cycling in a sediment microbial community. ISME J **7:**800-816.
49. 49. **Harris JK, Caporaso JG, Walker JJ, Spear JR, Gold NJ, Robertson CE, Hugenholtz P, Goodrich J, McDonald D, Knights D, Marshall P, Tufo H, Knight R, Pace NR.** 2013. Phylogenetic stratigraphy in the Guerrero Negro hypersaline microbial mat. ISME J **7:**50-60.
50. 50. **Jensen SI, Kuhl M, Prieme A.** 2007. Different bacterial communities associated with the roots and bulk sediment of the seagrass *Zostera marina*. FEMS Microbiol Ecol **62:**108-117.
51. 51. **Jiang L, Zheng Y, Peng X, Zhou H, Zhang C, Xiao X, Wang F.** 2009. Vertical distribution and diversity of sulfate-reducing prokaryotes in the Pearl River estuarine sediments, Southern China. FEMS Microbiol Ecol **70:**93-106.
52. 52. **Kochling T, Lara-Martin P, Gonzalez-Mazo E, Amils R, Sanz JL.** 2011. Microbial community composition of anoxic marine sediments in the Bay of Cadiz (Spain). Int Microbiol **14:**143-154.
53. 53. **Kojima H, Tsutsumi M, Ishikawa K, Iwata T, Mussmann M, Fukui M.** 2012. Distribution of putative denitrifying methane oxidizing bacteria in sediment of a freshwater lake, Lake Biwa. Syst Appl Microbiol **35:**233-238.
54. 54. **Lloyd KG, Albert DB, Biddle JF, Chanton JP, Pizarro O, Teske A.** 2010. Spatial structure and activity of sedimentary microbial communities underlying a Beggiatoa spp. mat in a Gulf of Mexico hydrocarbon seep. PLoS One **5:**e8738.
55. 55. **Nercessian O, Noyes E, Kalyuzhnaya MG, Lidstrom ME, Chistoserdova L.** 2005. Bacterial populations active in metabolism of C1 compounds in the sediment of Lake Washington, a freshwater lake. Appl Environ Microbiol **71:**6885-6899.
56. 56. **Omoregie EO, Mastalerz V, de Lange G, Straub KL, Kappler A, Roy H, Stadnitskaia A, Foucher JP, Boetius A.** 2008. Biogeochemistry and community composition of iron- and sulfur-precipitating microbial mats at the Chefren mud volcano (Nile Deep Sea Fan, Eastern Mediterranean). Appl Environ Microbiol **74:**3198-3215.
57. 57. **Orcutt BN, Joye SB, Kleindienst S, Knittel K, Ramette A, Reitz A, Samarkin V, Treude T, Boetius A.** 2010. Impact of natural oil and higher hydrocarbons on microbial diversity, distribution, and activity in Gulf of Mexico cold-seep sediments. Deep Sea Research Part II: Topical Studies in Oceanography **57:**2008-2021.
58. 58. **Pachiadaki MG, Kallionaki A, Dahlmann A, De Lange GJ, Kormas KA.** 2011. Diversity and spatial distribution of prokaryotic communities along a sediment vertical profile of a deep-sea mud volcano. Microb Ecol **62:**655-668.
59. 59. **Redmond MC, Valentine DL, Sessions AL.** 2010. Identification of novel methane-, ethane-, and propane-oxidizing bacteria at marine hydrocarbon seeps by stable isotope probing. Appl Environ Microbiol **76:**6412-6422.
60. 60. **Redmond MC, Valentine DL.** 2012. Natural gas and temperature structured a microbial community response to the Deepwater Horizon oil spill. Proc Natl Acad Sci USA **109:**20292-20297.
61. 61. **Sahl JW, Fairfield N, Harris JK, Wettergreen D, Stone WC, Spear JR.** 2010. Novel microbial diversity retrieved by autonomous robotic exploration of the world's deepest vertical phreatic sinkhole. Astrobiology **10:**201-213.
62. 62. **Schauer R, Roy H, Augustin N, Gennerich HH, Peters M, Wenzhoefer F, Amann R, Meyerdierks A.** 2011. Bacterial sulfur cycling shapes microbial communities in surface sediments of an ultramafic hydrothermal vent field. Environ Microbiol **13:**2633-2648.
63. 63. **Schmidtova J, Hallam SJ, Baldwin SA.** 2009. Phylogenetic diversity of transition and anoxic zone bacterial communities within a near-shore anoxic basin: Nitinat Lake. Environ Microbiol **11:**3233-3251.
64. 64. **Schreiber L, Holler T, Knittel K, Meyerdierks A, Amann R.** 2010. Identification of the dominant sulfate-reducing bacterial partner of anaerobic methanotrophs of the ANME-2 clade. Environ Microbiol **12:**2327-2340.
65. 65. **Sudek LA, Templeton AS, Tebo BM, Staudigel H.** 2009. Microbial Ecology of Fe (hydr)oxide Mats and Basaltic Rock from Vailulu'u Seamount, American Samoa. Geomicrobiol J **26:**581-596.
66. 66. **Woebken D, Lam P, Kuypers MM, Naqvi SW, Kartal B, Strous M, Jetten MS, Fuchs BM, Amann R.** 2008. A microdiversity study of anammox bacteria reveals a novel Candidatus Scalindua phylotype in marine oxygen minimum zones. Environ Microbiol **10:**3106-3119.
67. 67. **Yinxin Z, Yang Z, Jacqueline MG, Jianfeng H, Tianling Z.** 2011. Culture-independent and -dependent methods to investigate the diversity of planktonic bacteria in the northern Bering Sea. Pol Biol **35:**117-129.
68. 68. **Zeng Y, Zou Y, Chen B, Grebmeier J, Li H, Yu Y, Zheng T.** 2011. Phylogenetic diversity of sediment bacteria in the northern Bering Sea. Pol Biol **34:**907-919.
69. 69. **Kong HH, Oh J, Deming C, Conlan S, Grice EA, Beatson MA, Nomicos E, Polley EC, Komarow HD, Murray PR, Turner ML, Segre JA.** 2012. Temporal shifts in the skin microbiome associated with disease flares and treatment in children with atopic dermatitis. Genome Res **22:**850-859.
70. 70. **Macalady JL, Jones DS, Lyon EH.** 2007. Extremely acidic, pendulous cave wall biofilms from the Frasassi cave system, Italy. Environ Microbiol **9:**1402-1414.
71. 71. **Qi X, Wang E, Xing M, Zhao W, Chen X.** 2012. Rhizosphere and non-rhizosphere bacterial community composition of the wild medicinal plant *Rumex patientia*. World J Microbiol Biotechnol **28:**2257-2265.
72. 72. **Santelli CM, Orcutt BN, Banning E, Bach W, Moyer CL, Sogin ML, Staudigel H, Edwards KJ.** 2008. Abundance and diversity of microbial life in ocean crust. Nature **453:**653-656.
73. 73. **Song H, Li Z, Du B, Wang G, Ding Y.** 2012. Bacterial communities in sediments of the shallow Lake Dongping in China. J Appl Microbiol **112:**79-89.
74. 74. **Sunagawa S, Woodley CM, Medina M.** 2010. Threatened corals provide underexplored microbial habitats. PLoS One **5:**e9554.
75. 75. **Elshahed MS, Youssef NH, Spain AM, Sheik C, Najar FZ, Sukharnikov LO, Roe BA, Davis JP, Schloss PD, Bailey VL, Krumholz LR.** 2008. Novelty and uniqueness patterns of rare members of the soil biosphere. Appl Environ Microbiol **74:**5422-5428.
76. 76. **Hubert C, Loy A, Nickel M, Arnosti C, Baranyi C, Bruchert V, Ferdelman T, Finster K, Christensen FM, Rosa de Rezende J, Vandieken V, Jorgensen BB.** 2009. A constant flux of diverse thermophilic bacteria into the cold Arctic seabed. Science **325:**1541-1544.
77. 77. **McAllister SM, Davis RE, McBeth JM, Tebo BM, Emerson D, Moyer CL.** 2011. Biodiversity and emerging biogeography of the neutrophilic iron-oxidizing Zetaproteobacteria. Appl Environ Microbiol **77:**5445-5457.
78. 78. **Militon C, Boucher D, Vachelard C, Perchet G, Barra V, Troquet J, Peyretaillade E, Peyret P.** 2010. Bacterial community changes during bioremediation of aliphatic hydrocarbon-contaminated soil. FEMS Microbiol Ecol **74:**669-681.
79. 79. **Schauer R, Bienhold C, Ramette A, Harder J.** 2010. Bacterial diversity and biogeography in deep-sea surface sediments of the South Atlantic Ocean. ISME J **4:**159-170.
80. 80. **Schwarz JI, Eckert W, Conrad R.** 2007. Community structure of Archaea and Bacteria in a profundal lake sediment Lake Kinneret (Israel). Syst Appl Microbiol **30:**239-254.
81. 81. **Dillon J, McMath L, Trout A.** 2009. Seasonal changes in bacterial diversity in the Salton Sea. Hydrobiologia **632:**49-64.
82. 82. **Kato S, Chan C, Itoh T, Ohkuma M.** 2013. Functional gene analysis of freshwater iron-rich flocs at circumneutral pH and isolation of a stalk-forming microaerophilic iron-oxidizing bacterium. Appl Environ Microbiol **79:**5283-5290.
83. 83. **Kim SJ, Koh DC, Park SJ, Cha IT, Park JW, Na JH, Roh Y, Ko KS, Kim K, Rhee SK.** 2012. Molecular analysis of spatial variation of iron-reducing bacteria in riverine alluvial aquifers of the Mankyeong River. J Microbiol **50:**207-217.
84. 84. **Isenbarger TA, Finney M, Rios-Velazquez C, Handelsman J, Ruvkun G.** 2008. Miniprimer PCR, a new lens for viewing the microbial world. Appl Environ Microbiol **74:**840-849.
85. 85. **Schottner S, Pfitzner B, Grunke S, Rasheed M, Wild C, Ramette A.** 2011. Drivers of bacterial diversity dynamics in permeable carbonate and silicate coral reef sands from the Red Sea. Environ Microbiol **13:**1815-1826.
86. 86. **Amaral-Zettler LA, Rocca JD, Lamontagne MG, Dennett MR, Gast RJ.** 2008. Changes in microbial community structure in the wake of Hurricanes Katrina and Rita. Environ Sci Technol **42:**9072-9078.
87. 87. **Costello EK, Halloy SR, Reed SC, Sowell P, Schmidt SK.** 2009. Fumarole-supported islands of biodiversity within a hyperarid, high-elevation landscape on Socompa Volcano, Puna de Atacama, Andes. Appl Environ Microbiol **75:**735-747.
88. 88. **Cruz-Martinez K, Suttle KB, Brodie EL, Power ME, Andersen GL, Banfield JF.** 2009. Despite strong seasonal responses, soil microbial consortia are more resilient to long-term changes in rainfall than overlying grassland. ISME J **3:**738-744.
89. 89. **Du J, Xiao K, Huang Y, Li H, Tan H, Cao L, Lu Y, Zhou S.** 2011. Seasonal and spatial diversity of microbial communities in marine sediments of the South China Sea. Antonie Van Leeuwenhoek **100:**317-331.
90. 90. **Dunbar J, Eichorst SA, Gallegos-Graves LV, Silva S, Xie G, Hengartner NW, Evans RD, Hungate BA, Jackson RB, Megonigal JP, Schadt CW, Vilgalys R, Zak DR, Kuske CR.** 2012. Common bacterial responses in six ecosystems exposed to 10 years of elevated atmospheric carbon dioxide. Environ Microbiol **14:**1145-1158.
91. 91. **Flynn TM, Sanford RA, Ryu H, Bethke CM, Levine AD, Ashbolt NJ, Santo Domingo JW.** 2013. Functional microbial diversity explains groundwater chemistry in a pristine aquifer. BMC Microbiol **13:**146.
92. 92. **Garren M, Raymundo L, Guest J, Harvell CD, Azam F.** 2009. Resilience of coral-associated bacterial communities exposed to fish farm effluent. PLoS One **4:**e7319.
93. 93. **Gordon-Bradley N, Lymperopoulou DS, Williams HN.** 2014. Differences in bacterial community structure on Hydrilla verticillata and Vallisneria americana in a freshwater spring. Microbes Environ **29:**67-73.
94. 94. **Graff A, Conrad R.** 2005. Impact of flooding on soil bacterial communities associated with poplar (Populus sp.) trees. FEMS Microbiol Ecol **53:**401-415.
95. 95. **Haldar S, Choudhury SR, Sengupta S.** 2011. Genetic and functional diversities of bacterial communities in the rhizosphere of Arachis hypogaea. Antonie Van Leeuwenhoek **100:**161-170.
96. 96. **Hansel CM, Fendorf S, Jardine PM, Francis CA.** 2008. Changes in bacterial and archaeal community structure and functional diversity along a geochemically variable soil profile. Appl Environ Microbiol **74:**1620-1633.
97. 97. **Itoh H, Ishii S, Shiratori Y, Oshima K, Otsuka S, Hattori M, Senoo K.** 2013. Seasonal transition of active bacterial and archaeal communities in relation to water management in paddy soils. Microbes Environ **28:**370-380.
98. 98. **Kotik M, Famerova V.** 2012. Sequence diversity in haloalkane dehalogenases, as revealed by PCR using family-specific primers. J Microbiol Meth **88:**212-217.
99. 99. **Lesaulnier C, Papamichail D, McCorkle S, Ollivier B, Skiena S, Taghavi S, Zak D, van der Lelie D.** 2008. Elevated atmospheric CO_2_ affects soil microbial diversity associated with trembling aspen. Environ Microbiol **10:**926-941.
100. 100. **Mills HJ, Hunter E, Humphrys M, Kerkhof L, McGuinness L, Huettel M, Kostka JE.** 2008. Characterization of nitrifying, denitrifying, and overall bacterial communities in permeable marine sediments of the northeastern Gulf of Mexico. Appl Environ Microbiol **74:**4440-4453.
101. 101. **Ng C-C, Huang W-C, Chang C-C, Tzeng W-S, Chen T-W, Liu Y-S, Shyu Y-T.** 2006. Tufa microbial diversity revealed by 16S rRNA cloning in Taroko National Park, Taiwan. Soil Biol Biochem **38:**342-348.
102. 102. **Santo Domingo JW, Revetta RP, Iker B, Gomez-Alvarez V, Garcia J, Sullivan J, Weast J.** 2011. Molecular survey of concrete sewer biofilm microbial communities. Biofouling **27:**993-1001.
103. 103. **Shivaji S, Pratibha MS, Sailaja B, Hara Kishore K, Singh AK, Begum Z, Anarasi U, Prabagaran SR, Reddy GS, Srinivas TN.** 2011. Bacterial diversity of soil in the vicinity of Pindari glacier, Himalayan mountain ranges, India, using culturable bacteria and soil 16S rRNA gene clones. Extremophiles **15:**1-22.
104. 104. **Wust PK, Horn MA, Drake HL.** 2011. Clostridiaceae and Enterobacteriaceae as active fermenters in earthworm gut content. ISME J **5:**92-106.
105. 105. **Yoon DN, Park SJ, Kim SJ, Jeon CO, Chae JC, Rhee SK.** 2010. Isolation, characterization, and abundance of filamentous members of Caldilineae in activated sludge. J Microbiol **48:**275-283.
106. 106. **Yuan Y, Si G, Wang J, Luo T, Zhang G.** 2014. Bacterial community in alpine grasslands along an altitudinal gradient on the Tibetan Plateau. FEMS Microbiol Ecol **87:**121-132.
107. 107. **Zeng J, Yang HM, Lou K.** 2010. Prokaryotic diversity of a non-sulfide, low-salt cold spring sediment of Shawan County, China. J Basic Microbiol **50:**484-493.
108. 108. **Zhao D, Huang R, Zeng J, Yan W, Wang J, Ma T, Wang M, Wu QL.** 2012. Diversity analysis of bacterial community compositions in sediments of urban lakes by terminal restriction fragment length polymorphism (T-RFLP). World J Microbiol Biotechnol **28:**3159-3170.
109. 109. **Zhao DY, Liu P, Fang C, Sun YM, Zeng J, Wang JQ, Ma T, Xiao YH, Wu QL.** 2013. Submerged macrophytes modify bacterial community composition in sediments in a large, shallow, freshwater lake. Can J Microbiol **59:**237-244.
110. 110. **Acosta-Gonzalez A, Rossello-Mora R, Marques S.** 2013. Characterization of the anaerobic microbial community in oil-polluted subtidal sediments: aromatic biodegradation potential after the Prestige oil spill. Environ Microbiol **15:**77-92.
111. 111. **Baniulyte D, Favila E, Kelly JJ.** 2009. Shifts in microbial community composition following surface application of dredged river sediments. Microb Ecol **57:**160-169.
112. 112. **Bernard L, Mougel C, Maron PA, Nowak V, Leveque J, Henault C, Haichar FZ, Berge O, Marol C, Balesdent J, Gibiat F, Lemanceau P, Ranjard L.** 2007. Dynamics and identification of soil microbial populations actively assimilating carbon from 13C-labelled wheat residue as estimated by DNA- and RNA-SIP techniques. Environ Microbiol **9:**752-764.
113. 113. **Falteisek L, Cepicka I.** 2012. Microbiology of diverse acidic and non-acidic microhabitats within a sulfidic ore mine. Extremophiles **16:**911-922.
114. 114. **Gillan DC, Pernet P.** 2007. Adherent bacteria in heavy metal contaminated marine sediments. Biofouling **23:**1-13.
115. 115. **Hao da C, Ge GB, Yang L.** 2008. Bacterial diversity of Taxus rhizosphere: culture-independent and culture-dependent approaches. FEMS Microbiol Lett **284:**204-212.
116. 116. **Hunger S, Schmidt O, Hilgarth M, Horn MA, Kolb S, Conrad R, Drake HL.** 2011. Competing formate- and carbon dioxide-utilizing prokaryotes in an anoxic methane-emitting fen soil. Appl Environ Microbiol **77:**3773-3785.
117. 117. **Ishii S, Hotta Y, Watanabe K.** 2008. Methanogenesis versus electrogenesis: morphological and phylogenetic comparisons of microbial communities. Biosci Biotechnol Biochem **72:**286-294.
118. 118. **Kato S, Nakawake M, Ohkuma M, Yamagishi A.** 2012. Distribution and phylogenetic diversity of cbbM genes encoding RubisCO form II in a deep-sea hydrothermal field revealed by newly designed PCR primers. Extremophiles **16:**277-283.
119. 119. **KB S, B G, A H, E G.** 2007. Spatial structure of the microbial community in sandy carbonate sediment. Marine Ecology Progress Series **346:**61-74.
120. 120. **LaPara TM, Nakatsu CH, Pantea L, Alleman JE.** 2000. Phylogenetic analysis of bacterial communities in mesophilic and thermophilic bioreactors treating pharmaceutical wastewater. Appl Environ Microbiol **66:**3951-3959.
121. 121. **Singleton DR, Sangaiah R, Gold A, Ball LM, Aitken MD.** 2006. Identification and quantification of uncultivated Proteobacteria associated with pyrene degradation in a bioreactor treating PAH-contaminated soil. Environ Microbiol **8:**1736-1745.
122. 122. **Tringe SG, von Mering C, Kobayashi A, Salamov AA, Chen K, Chang HW, Podar M, Short JM, Mathur EJ, Detter JC, Bork P, Hugenholtz P, Rubin EM.** 2005. Comparative metagenomics of microbial communities. Science **308:**554-557.
123. 123. **Gan Y, Qiu Q, Liu P, Rui J, Lu Y.** 2012. Syntrophic oxidation of propionate in rice field soil at 15 and 30 degrees C under methanogenic conditions. Appl Environ Microbiol **78:**4923-4932.
124. 124. **Holmes AJ, Tujula NA, Holley M, Contos A, James JM, Rogers P, Gillings MR.** 2001. Phylogenetic structure of unusual aquatic microbial formations in Nullarbor caves, Australia. Environ Microbiol **3:**256-264.
125. 125. **Jiang DM, Kato C, Zhou XW, Wu ZH, Sato T, Li YZ.** 2010. Phylogeographic separation of marine and soil myxobacteria at high levels of classification. ISME J **4:**1520-1530.
126. 126. **Lau MC, Aitchison JC, Pointing SB.** 2009. Bacterial community composition in thermophilic microbial mats from five hot springs in central Tibet. Extremophiles **13:**139-149.
127. 127. **Teske A, Durbin A, Ziervogel K, Cox C, Arnosti C.** 2011. Microbial community composition and function in permanently cold seawater and sediments from an arctic fjord of svalbard. Appl Environ Microbiol **77:**2008-2018.
128. 128. **Edlund A, Hardeman F, Jansson JK, Sjoling S.** 2008. Active bacterial community structure along vertical redox gradients in Baltic Sea sediment. Environ Microbiol **10:**2051-2063.
129. 129. **Lloyd KG, Lapham L, Teske A.** 2006. An anaerobic methane-oxidizing community of ANME-1b archaea in hypersaline Gulf of Mexico sediments. Appl Environ Microbiol **72:**7218-7230.
130. 130. **Nunoura T, Oida H, Nakaseama M, Kosaka A, Ohkubo SB, Kikuchi T, Kazama H, Hosoi-Tanabe S, Nakamura K, Kinoshita M, Hirayama H, Inagaki F, Tsunogai U, Ishibashi J, Takai K.** 2010. Archaeal diversity and distribution along thermal and geochemical gradients in hydrothermal sediments at the Yonaguni Knoll IV hydrothermal field in the Southern Okinawa trough. Appl Environ Microbiol **76:**1198-1211.
131. 131. **Powell SM, Bowman JP, Snape I, Stark JS.** 2003. Microbial community variation in pristine and polluted nearshore Antarctic sediments. FEMS Microbiol Ecol **45:**135-145.
132. 132. **Schwarz JI, Lueders T, Eckert W, Conrad R.** 2007. Identification of acetate-utilizing Bacteria and Archaea in methanogenic profundal sediments of Lake Kinneret (Israel) by stable isotope probing of rRNA. Environ Microbiol **9:**223-237.
133. 133. **Sitte J, Akob DM, Kaufmann C, Finster K, Banerjee D, Burkhardt EM, Kostka JE, Scheinost AC, Buchel G, Kusel K.** 2010. Microbial links between sulfate reduction and metal retention in uranium- and heavy metal-contaminated soil. Appl Environ Microbiol **76:**3143-3152.
134. 134. **Xing W, Zhao Y, Zuo JE.** 2010. Microbial activity and community structure in a lake sediment used for psychrophilic anaerobic wastewater treatment. J Appl Microbiol **109:**1829-1837.
135. 135. **Yan Z, Song N, Cai H, Tay JH, Jiang H.** 2012. Enhanced degradation of phenanthrene and pyrene in freshwater sediments by combined employment of sediment microbial fuel cell and amorphous ferric hydroxide. J Hazard Mater **199-200:**217-225.
136. 136. **Bowman JP, McCuaig RD.** 2003. Biodiversity, community structural shifts, and biogeography of prokaryotes within Antarctic continental shelf sediment. Appl Environ Microbiol **69:**2463-2483.
137. 137. **Brodie EL, Desantis TZ, Joyner DC, Baek SM, Larsen JT, Andersen GL, Hazen TC, Richardson PM, Herman DJ, Tokunaga TK, Wan JM, Firestone MK.** 2006. Application of a high-density oligonucleotide microarray approach to study bacterial population dynamics during uranium reduction and reoxidation. Appl Environ Microbiol **72:**6288-6298.
138. 138. **Dojka MA, Hugenholtz P, Haack SK, Pace NR.** 1998. Microbial diversity in a hydrocarbon- and chlorinated-solvent-contaminated aquifer undergoing intrinsic bioremediation. Appl Environ Microbiol **64:**3869-3877.
139. 139. **Durbin AM, Teske A.** 2011. Microbial diversity and stratification of South Pacific abyssal marine sediments. Environ Microbiol **13:**3219-3234.
140. 140. **Engel AS, Meisinger DB, Porter ML, Payn RA, Schmid M, Stern LA, Schleifer KH, Lee NM.** 2010. Linking phylogenetic and functional diversity to nutrient spiraling in microbial mats from Lower Kane Cave (USA). ISME J **4:**98-110.
141. 141. **Ferrer M, Guazzaroni ME, Richter M, Garcia-Salamanca A, Yarza P, Suarez-Suarez A, Solano J, Alcaide M, van Dillewijn P, Molina-Henares MA, Lopez-Cortes N, Al-Ramahi Y, Guerrero C, Acosta A, de Eugenio LI, Martinez V, Marques S, Rojo F, Santero E, Genilloud O, Perez-Perez J, Rossello-Mora R, Ramos JL.** 2011. Taxonomic and functional metagenomic profiling of the microbial community in the anoxic sediment of a sub-saline shallow lake (Laguna de Carrizo, Central Spain). Microb Ecol **62:**824-837.
142. 142. **Ho CH, Liu SM.** 2010. Impact of coplanar PCBs on microbial communities in anaerobic estuarine sediments. J Environ Sci Health B **45:**437-448.
143. 143. **Hoshino T, Morono Y, Terada T, Imachi H, Ferdelman TG, Inagaki F.** 2011. Comparative study of subseafloor microbial community structures in deeply buried coral fossils and sediment matrices from the challenger mound in the porcupine seabight. Front Microbiol **2:**231.
144. 144. **Hunter EM, Mills HJ, Kostka JE.** 2006. Microbial community diversity associated with carbon and nitrogen cycling in permeable shelf sediments. Appl Environ Microbiol **72:**5689-5701.
145. 145. **Inagaki F, Suzuki M, Takai K, Oida H, Sakamoto T, Aoki K, Nealson KH, Horikoshi K.** 2003. Microbial communities associated with geological horizons in coastal subseafloor sediments from the sea of okhotsk. Appl Environ Microbiol **69:**7224-7235.
146. 146. **Inagaki F, Nunoura T, Nakagawa S, Teske A, Lever M, Lauer A, Suzuki M, Takai K, Delwiche M, Colwell FS, Nealson KH, Horikoshi K, D'Hondt S, Jorgensen BB.** 2006. Biogeographical distribution and diversity of microbes in methane hydrate-bearing deep marine sediments on the Pacific Ocean Margin. Proc Natl Acad Sci USA **103:**2815-2820.
147. 147. **Ley RE, Harris JK, Wilcox J, Spear JR, Miller SR, Bebout BM, Maresca JA, Bryant DA, Sogin ML, Pace NR.** 2006. Unexpected diversity and complexity of the Guerrero Negro hypersaline microbial mat. Appl Environ Microbiol **72:**3685-3695.
148. 148. **Li T, Wang P.** 2013. Richness and diversity of bacteria in the Nansha carbonate platform (Core MD05-2896), South China Sea. World J Microbiol Biotechnol **29:**1895-1905.
149. 149. **Nunoura T, Soffientino B, Blazejak A, Kakuta J, Oida H, Schippers A, Takai K.** 2009. Subseafloor microbial communities associated with rapid turbidite deposition in the Gulf of Mexico continental slope (IODP Expedition 308). FEMS Microbiol Ecol **69:**410-424.
150. 150. **Nunoura T, Nishizawa M, Kikuchi T, Tsubouchi T, Hirai M, Koide O, Miyazaki J, Hirayama H, Koba K, Takai K.** 2013. Molecular biological and isotopic biogeochemical prognoses of the nitrification-driven dynamic microbial nitrogen cycle in hadopelagic sediments. Environ Microbiol doi:10.1111/1462-2920.12152.
151. 151. **Shu Q, Jiao N.** 2008. Profiling Planctomycetales diversity with reference to anammox-related bacteria in a South China Sea, deep-sea sediment. Mar Ecol **29:**413-420.
152. 152. **Vasconcelos C, McKenzie JA.** 1997. Microbial mediation of modern dolomite precipitation and diagenesis under anoxic conditions (Lagoa Vermelha, Rio de Janeiro, Brazil). J Sed Res **67:**378-390.
153. 153. **Walsh DA, Zaikova E, Howes CG, Song YC, Wright JJ, Tringe SG, Tortell PD, Hallam SJ.** 2009. Metagenome of a versatile chemolithoautotroph from expanding oceanic dead zones. Science **326:**578-582.

Table A. Genbank accession numbers, candidate order, and study site of all near-full-length 16S rRNA gene sequences affiliated with “Latescibacteria” that were used to construct phylogenetic trees shown in Fig. 1.

| WS3 Class | Accessions | Study | Reference |
| --- | --- | --- | --- |
| c PRR-12 Unclass | DQ103596.1 | Mud volcano sediments | (38) |
| c PRR-12 Unclass | DQ103598.1 | Mud volcano sediments | (38) |
| c PRR-12 Unclass | HM186623.1 | Hanford Site subsurface sediment | (39) |
| c PRR-12 Unclass | FJ478657.1 | Undisturbed tall grass prairie | (40) |
| c PRR-12 Unclass | EU133901.1 | Soil from an undisturbed mixed grass prairie preserve | Unpublished |
| c PRR-12 Unclass | EU135574.1 | Soil from an undisturbed mixed grass prairie preserve | Unpublished |
| c PRR-12 Unclass | EU133583.1 | Soil from an undisturbed mixed grass prairie preserve | Unpublished |
| c PRR-12 Unclass | AB252949.1 | Japan: Ishikawa, Kaga, Lagoon Shibayama | Unpublished |
| c PRR-12 Unclass | EU135577.1 | Soil from an undisturbed mixed grass prairie preserve | Unpublished |
| c PRR-12 Unclass | JN860302.1 | Low temperature hydrothermal oxides at the South West Indian Ridge | Unpublished |
| c PRR-12 Unclass | JN860303.1 | Low temperature hydrothermal oxides at the South West Indian Ridge | Unpublished |
| GNO3 | FJ264743.1 | Methane seep sediment | (41) |
| GNO3 | FJ516882.1 | The semiarid 'Tablas de Daimiel National Park' wetland | (42) |
| GNO3 | FJ516997.1 | The semiarid 'Tablas de Daimiel National Park' wetland | (42) |
| GNO3 | FJ517000.1 | The semiarid 'Tablas de Daimiel National Park' wetland | (42) |
| GNO3 | AJ390441.1 | Soil and rice roots of flooded rice microcosms | (43) |
| GNO3 | AJ390446.1 | Soil and rice roots of flooded rice microcosms | (43) |
| GNO3 | FJ905749.1 | Iron oxide sediments, Volcano 1, Tonga Arc | (44) |
| GNO3 | AY114313.1 | Anoxic marine sediment | (45) |
| GNO3 | AY114319.1 | Anoxic marine sediment | (45) |
| GNO3 | GU145525.1 | Black Sea | (46) |
| GNO3 | GU145538.1 | Black Sea | (46) |
| GNO3 | EU488056.1 | Siliciclastic sedment from Thalassia sea grass bed | (47) |
| GNO3 | JX120387.1 | Subsurface aquifer sediment | (48) |
| GNO3 | JN429245.1 | Guerrero Negro hypersaline microbial mat | (49) |
| GNO3 | JN429447.1 | Guerrero Negro hypersaline microbial mat | (49) |
| GNO3 | JN429592.1 | Guerrero Negro hypersaline microbial mat | (49) |
| GNO3 | JN430002.1 | Guerrero Negro hypersaline microbial mat | (49) |
| GNO3 | JN444892.1 | Guerrero Negro hypersaline microbial mat | (49) |
| GNO3 | JN446912.1 | Guerrero Negro hypersaline microbial mat | (49) |
| GNO3 | JN447570.1 | Guerrero Negro hypersaline microbial mat | (49) |
| GNO3 | JN449268.1 | Guerrero Negro hypersaline microbial mat | (49) |
| GNO3 | JN457169.1 | Guerrero Negro hypersaline microbial mat | (49) |
| GNO3 | JN457618.1 | Guerrero Negro hypersaline microbial mat | (49) |
| GNO3 | JN461796.1 | Guerrero Negro hypersaline microbial mat | (49) |
| GNO3 | JN461979.1 | Guerrero Negro hypersaline microbial mat | (49) |
| GNO3 | JN462028.1 | Guerrero Negro hypersaline microbial mat | (49) |
| GNO3 | JN462653.1 | Guerrero Negro hypersaline microbial mat | (49) |
| GNO3 | JN464025.1 | Guerrero Negro hypersaline microbial mat | (49) |
| GNO3 | JN464165.1 | Guerrero Negro hypersaline microbial mat | (49) |
| GNO3 | JN467855.1 | Guerrero Negro hypersaline microbial mat | (49) |
| GNO3 | JN468501.1 | Guerrero Negro hypersaline microbial mat | (49) |
| GNO3 | JN470792.1 | Guerrero Negro hypersaline microbial mat | (49) |
| GNO3 | JN474002.1 | Guerrero Negro hypersaline microbial mat | (49) |
| GNO3 | JN474116.1 | Guerrero Negro hypersaline microbial mat | (49) |
| GNO3 | JN475020.1 | Guerrero Negro hypersaline microbial mat | (49) |
| GNO3 | JN476530.1 | Guerrero Negro hypersaline microbial mat | (49) |
| GNO3 | JN476597.1 | Guerrero Negro hypersaline microbial mat | (49) |
| GNO3 | JN476852.1 | Guerrero Negro hypersaline microbial mat | (49) |
| GNO3 | JN476888.1 | Guerrero Negro hypersaline microbial mat | (49) |
| GNO3 | JN477139.1 | Guerrero Negro hypersaline microbial mat | (49) |
| GNO3 | JN477214.1 | Guerrero Negro hypersaline microbial mat | (49) |
| GNO3 | JN477371.1 | Guerrero Negro hypersaline microbial mat | (49) |
| GNO3 | JN479349.1 | Guerrero Negro hypersaline microbial mat | (49) |
| GNO3 | JN479446.1 | Guerrero Negro hypersaline microbial mat | (49) |
| GNO3 | JN479559.1 | Guerrero Negro hypersaline microbial mat | (49) |
| GNO3 | JN479688.1 | Guerrero Negro hypersaline microbial mat | (49) |
| GNO3 | JN480170.1 | Guerrero Negro hypersaline microbial mat | (49) |
| GNO3 | JN480173.1 | Guerrero Negro hypersaline microbial mat | (49) |
| GNO3 | JN481368.1 | Guerrero Negro hypersaline microbial mat | (49) |
| GNO3 | JN482310.1 | Guerrero Negro hypersaline microbial mat | (49) |
| GNO3 | JN482402.1 | Guerrero Negro hypersaline microbial mat | (49) |
| GNO3 | JN483368.1 | Guerrero Negro hypersaline microbial mat | (49) |
| GNO3 | JN483573.1 | Guerrero Negro hypersaline microbial mat | (49) |
| GNO3 | JN483968.1 | Guerrero Negro hypersaline microbial mat | (49) |
| GNO3 | JN484281.1 | Guerrero Negro hypersaline microbial mat | (49) |
| GNO3 | JN484988.1 | Guerrero Negro hypersaline microbial mat | (49) |
| GNO3 | JN485564.1 | Guerrero Negro hypersaline microbial mat | (49) |
| GNO3 | JN485915.1 | Guerrero Negro hypersaline microbial mat | (49) |
| GNO3 | JN485948.1 | Guerrero Negro hypersaline microbial mat | (49) |
| GNO3 | JN486584.1 | Guerrero Negro hypersaline microbial mat | (49) |
| GNO3 | JN486871.1 | Guerrero Negro hypersaline microbial mat | (49) |
| GNO3 | JN486917.1 | Guerrero Negro hypersaline microbial mat | (49) |
| GNO3 | JN487303.1 | Guerrero Negro hypersaline microbial mat | (49) |
| GNO3 | JN487420.1 | Guerrero Negro hypersaline microbial mat | (49) |
| GNO3 | JN487428.1 | Guerrero Negro hypersaline microbial mat | (49) |
| GNO3 | JN487500.1 | Guerrero Negro hypersaline microbial mat | (49) |
| GNO3 | JN487812.1 | Guerrero Negro hypersaline microbial mat | (49) |
| GNO3 | JN488122.1 | Guerrero Negro hypersaline microbial mat | (49) |
| GNO3 | JN488395.1 | Guerrero Negro hypersaline microbial mat | (49) |
| GNO3 | JN488475.1 | Guerrero Negro hypersaline microbial mat | (49) |
| GNO3 | JN489415.1 | Guerrero Negro hypersaline microbial mat | (49) |
| GNO3 | JN489522.1 | Guerrero Negro hypersaline microbial mat | (49) |
| GNO3 | JN490832.1 | Guerrero Negro hypersaline microbial mat | (49) |
| GNO3 | JN490843.1 | Guerrero Negro hypersaline microbial mat | (49) |
| GNO3 | JN491553.1 | Guerrero Negro hypersaline microbial mat | (49) |
| GNO3 | JN491950.1 | Guerrero Negro hypersaline microbial mat | (49) |
| GNO3 | JN492332.1 | Guerrero Negro hypersaline microbial mat | (49) |
| GNO3 | JN492761.1 | Guerrero Negro hypersaline microbial mat | (49) |
| GNO3 | JN492998.1 | Guerrero Negro hypersaline microbial mat | (49) |
| GNO3 | JN493140.1 | Guerrero Negro hypersaline microbial mat | (49) |
| GNO3 | JN493185.1 | Guerrero Negro hypersaline microbial mat | (49) |
| GNO3 | JN493216.1 | Guerrero Negro hypersaline microbial mat | (49) |
| GNO3 | JN493854.1 | Guerrero Negro hypersaline microbial mat | (49) |
| GNO3 | JN494125.1 | Guerrero Negro hypersaline microbial mat | (49) |
| GNO3 | JN495130.1 | Guerrero Negro hypersaline microbial mat | (49) |
| GNO3 | JN495444.1 | Guerrero Negro hypersaline microbial mat | (49) |
| GNO3 | JN495567.1 | Guerrero Negro hypersaline microbial mat | (49) |
| GNO3 | JN496604.1 | Guerrero Negro hypersaline microbial mat | (49) |
| GNO3 | JN497659.1 | Guerrero Negro hypersaline microbial mat | (49) |
| GNO3 | JN497718.1 | Guerrero Negro hypersaline microbial mat | (49) |
| GNO3 | JN497756.1 | Guerrero Negro hypersaline microbial mat | (49) |
| GNO3 | JN498065.1 | Guerrero Negro hypersaline microbial mat | (49) |
| GNO3 | JN498176.1 | Guerrero Negro hypersaline microbial mat | (49) |
| GNO3 | JN500471.1 | Guerrero Negro hypersaline microbial mat | (49) |
| GNO3 | JN501089.1 | Guerrero Negro hypersaline microbial mat | (49) |
| GNO3 | JN501156.1 | Guerrero Negro hypersaline microbial mat | (49) |
| GNO3 | JN501458.1 | Guerrero Negro hypersaline microbial mat | (49) |
| GNO3 | JN501781.1 | Guerrero Negro hypersaline microbial mat | (49) |
| GNO3 | JN502539.1 | Guerrero Negro hypersaline microbial mat | (49) |
| GNO3 | JN503475.1 | Guerrero Negro hypersaline microbial mat | (49) |
| GNO3 | JN504073.1 | Guerrero Negro hypersaline microbial mat | (49) |
| GNO3 | JN504424.1 | Guerrero Negro hypersaline microbial mat | (49) |
| GNO3 | JN504870.1 | Guerrero Negro hypersaline microbial mat | (49) |
| GNO3 | JN505062.1 | Guerrero Negro hypersaline microbial mat | (49) |
| GNO3 | JN505791.1 | Guerrero Negro hypersaline microbial mat | (49) |
| GNO3 | JN505985.1 | Guerrero Negro hypersaline microbial mat | (49) |
| GNO3 | JN506054.1 | Guerrero Negro hypersaline microbial mat | (49) |
| GNO3 | JN506089.1 | Guerrero Negro hypersaline microbial mat | (49) |
| GNO3 | JN506273.1 | Guerrero Negro hypersaline microbial mat | (49) |
| GNO3 | JN506380.1 | Guerrero Negro hypersaline microbial mat | (49) |
| GNO3 | JN506387.1 | Guerrero Negro hypersaline microbial mat | (49) |
| GNO3 | JN506447.1 | Guerrero Negro hypersaline microbial mat | (49) |
| GNO3 | JN506462.1 | Guerrero Negro hypersaline microbial mat | (49) |
| GNO3 | JN506548.1 | Guerrero Negro hypersaline microbial mat | (49) |
| GNO3 | JN506617.1 | Guerrero Negro hypersaline microbial mat | (49) |
| GNO3 | JN507362.1 | Guerrero Negro hypersaline microbial mat | (49) |
| GNO3 | JN507471.1 | Guerrero Negro hypersaline microbial mat | (49) |
| GNO3 | JN507587.1 | Guerrero Negro hypersaline microbial mat | (49) |
| GNO3 | JN507838.1 | Guerrero Negro hypersaline microbial mat | (49) |
| GNO3 | JN507861.1 | Guerrero Negro hypersaline microbial mat | (49) |
| GNO3 | JN507966.1 | Guerrero Negro hypersaline microbial mat | (49) |
| GNO3 | JN508598.1 | Guerrero Negro hypersaline microbial mat | (49) |
| GNO3 | JN508854.1 | Guerrero Negro hypersaline microbial mat | (49) |
| GNO3 | JN509042.1 | Guerrero Negro hypersaline microbial mat | (49) |
| GNO3 | JN509116.1 | Guerrero Negro hypersaline microbial mat | (49) |
| GNO3 | JN509129.1 | Guerrero Negro hypersaline microbial mat | (49) |
| GNO3 | JN509263.1 | Guerrero Negro hypersaline microbial mat | (49) |
| GNO3 | JN509411.1 | Guerrero Negro hypersaline microbial mat | (49) |
| GNO3 | JN509452.1 | Guerrero Negro hypersaline microbial mat | (49) |
| GNO3 | JN509464.1 | Guerrero Negro hypersaline microbial mat | (49) |
| GNO3 | JN509617.1 | Guerrero Negro hypersaline microbial mat | (49) |
| GNO3 | JN509621.1 | Guerrero Negro hypersaline microbial mat | (49) |
| GNO3 | JN509734.1 | Guerrero Negro hypersaline microbial mat | (49) |
| GNO3 | JN509754.1 | Guerrero Negro hypersaline microbial mat | (49) |
| GNO3 | JN509784.1 | Guerrero Negro hypersaline microbial mat | (49) |
| GNO3 | JN509956.1 | Guerrero Negro hypersaline microbial mat | (49) |
| GNO3 | JN509978.1 | Guerrero Negro hypersaline microbial mat | (49) |
| GNO3 | JN510111.1 | Guerrero Negro hypersaline microbial mat | (49) |
| GNO3 | JN510232.1 | Guerrero Negro hypersaline microbial mat | (49) |
| GNO3 | JN510350.1 | Guerrero Negro hypersaline microbial mat | (49) |
| GNO3 | JN510380.1 | Guerrero Negro hypersaline microbial mat | (49) |
| GNO3 | JN510409.1 | Guerrero Negro hypersaline microbial mat | (49) |
| GNO3 | JN510427.1 | Guerrero Negro hypersaline microbial mat | (49) |
| GNO3 | JN510541.1 | Guerrero Negro hypersaline microbial mat | (49) |
| GNO3 | JN510559.1 | Guerrero Negro hypersaline microbial mat | (49) |
| GNO3 | JN510702.1 | Guerrero Negro hypersaline microbial mat | (49) |
| GNO3 | JN510839.1 | Guerrero Negro hypersaline microbial mat | (49) |
| GNO3 | JN511012.1 | Guerrero Negro hypersaline microbial mat | (49) |
| GNO3 | JN511516.1 | Guerrero Negro hypersaline microbial mat | (49) |
| GNO3 | JN512008.1 | Guerrero Negro hypersaline microbial mat | (49) |
| GNO3 | JN512273.1 | Guerrero Negro hypersaline microbial mat | (49) |
| GNO3 | JN512308.1 | Guerrero Negro hypersaline microbial mat | (49) |
| GNO3 | JN512511.1 | Guerrero Negro hypersaline microbial mat | (49) |
| GNO3 | JN512824.1 | Guerrero Negro hypersaline microbial mat | (49) |
| GNO3 | JN512851.1 | Guerrero Negro hypersaline microbial mat | (49) |
| GNO3 | JN512946.1 | Guerrero Negro hypersaline microbial mat | (49) |
| GNO3 | JN513052.1 | Guerrero Negro hypersaline microbial mat | (49) |
| GNO3 | JN513078.1 | Guerrero Negro hypersaline microbial mat | (49) |
| GNO3 | JN513111.1 | Guerrero Negro hypersaline microbial mat | (49) |
| GNO3 | JN513368.1 | Guerrero Negro hypersaline microbial mat | (49) |
| GNO3 | JN513912.1 | Guerrero Negro hypersaline microbial mat | (49) |
| GNO3 | JN514388.1 | Guerrero Negro hypersaline microbial mat | (49) |
| GNO3 | JN514473.1 | Guerrero Negro hypersaline microbial mat | (49) |
| GNO3 | JN514475.1 | Guerrero Negro hypersaline microbial mat | (49) |
| GNO3 | JN514748.1 | Guerrero Negro hypersaline microbial mat | (49) |
| GNO3 | JN514850.1 | Guerrero Negro hypersaline microbial mat | (49) |
| GNO3 | JN515128.1 | Guerrero Negro hypersaline microbial mat | (49) |
| GNO3 | JN515369.1 | Guerrero Negro hypersaline microbial mat | (49) |
| GNO3 | JN515403.1 | Guerrero Negro hypersaline microbial mat | (49) |
| GNO3 | JN515466.1 | Guerrero Negro hypersaline microbial mat | (49) |
| GNO3 | JN515473.1 | Guerrero Negro hypersaline microbial mat | (49) |
| GNO3 | JN515744.1 | Guerrero Negro hypersaline microbial mat | (49) |
| GNO3 | JN515937.1 | Guerrero Negro hypersaline microbial mat | (49) |
| GNO3 | JN516064.1 | Guerrero Negro hypersaline microbial mat | (49) |
| GNO3 | JN516106.1 | Guerrero Negro hypersaline microbial mat | (49) |
| GNO3 | JN516216.1 | Guerrero Negro hypersaline microbial mat | (49) |
| GNO3 | JN516318.1 | Guerrero Negro hypersaline microbial mat | (49) |
| GNO3 | JN517258.1 | Guerrero Negro hypersaline microbial mat | (49) |
| GNO3 | JN517328.1 | Guerrero Negro hypersaline microbial mat | (49) |
| GNO3 | JN517379.1 | Guerrero Negro hypersaline microbial mat | (49) |
| GNO3 | JN517422.1 | Guerrero Negro hypersaline microbial mat | (49) |
| GNO3 | JN517484.1 | Guerrero Negro hypersaline microbial mat | (49) |
| GNO3 | JN517509.1 | Guerrero Negro hypersaline microbial mat | (49) |
| GNO3 | JN517564.1 | Guerrero Negro hypersaline microbial mat | (49) |
| GNO3 | JN517566.1 | Guerrero Negro hypersaline microbial mat | (49) |
| GNO3 | JN517593.1 | Guerrero Negro hypersaline microbial mat | (49) |
| GNO3 | JN517869.1 | Guerrero Negro hypersaline microbial mat | (49) |
| GNO3 | JN518037.1 | Guerrero Negro hypersaline microbial mat | (49) |
| GNO3 | JN518067.1 | Guerrero Negro hypersaline microbial mat | (49) |
| GNO3 | JN518101.1 | Guerrero Negro hypersaline microbial mat | (49) |
| GNO3 | JN518197.1 | Guerrero Negro hypersaline microbial mat | (49) |
| GNO3 | JN518199.1 | Guerrero Negro hypersaline microbial mat | (49) |
| GNO3 | JN518205.1 | Guerrero Negro hypersaline microbial mat | (49) |
| GNO3 | JN518229.1 | Guerrero Negro hypersaline microbial mat | (49) |
| GNO3 | JN518252.1 | Guerrero Negro hypersaline microbial mat | (49) |
| GNO3 | JN518318.1 | Guerrero Negro hypersaline microbial mat | (49) |
| GNO3 | JN518617.1 | Guerrero Negro hypersaline microbial mat | (49) |
| GNO3 | JN518648.1 | Guerrero Negro hypersaline microbial mat | (49) |
| GNO3 | JN518675.1 | Guerrero Negro hypersaline microbial mat | (49) |
| GNO3 | JN519065.1 | Guerrero Negro hypersaline microbial mat | (49) |
| GNO3 | JN519146.1 | Guerrero Negro hypersaline microbial mat | (49) |
| GNO3 | JN519277.1 | Guerrero Negro hypersaline microbial mat | (49) |
| GNO3 | JN519313.1 | Guerrero Negro hypersaline microbial mat | (49) |
| GNO3 | JN519317.1 | Guerrero Negro hypersaline microbial mat | (49) |
| GNO3 | JN519432.1 | Guerrero Negro hypersaline microbial mat | (49) |
| GNO3 | JN519493.1 | Guerrero Negro hypersaline microbial mat | (49) |
| GNO3 | JN519521.1 | Guerrero Negro hypersaline microbial mat | (49) |
| GNO3 | JN519542.1 | Guerrero Negro hypersaline microbial mat | (49) |
| GNO3 | JN519777.1 | Guerrero Negro hypersaline microbial mat | (49) |
| GNO3 | JN519784.1 | Guerrero Negro hypersaline microbial mat | (49) |
| GNO3 | JN519813.1 | Guerrero Negro hypersaline microbial mat | (49) |
| GNO3 | JN519910.1 | Guerrero Negro hypersaline microbial mat | (49) |
| GNO3 | JN519955.1 | Guerrero Negro hypersaline microbial mat | (49) |
| GNO3 | JN520137.1 | Guerrero Negro hypersaline microbial mat | (49) |
| GNO3 | JN520183.1 | Guerrero Negro hypersaline microbial mat | (49) |
| GNO3 | JN520356.1 | Guerrero Negro hypersaline microbial mat | (49) |
| GNO3 | JN520371.1 | Guerrero Negro hypersaline microbial mat | (49) |
| GNO3 | JN520621.1 | Guerrero Negro hypersaline microbial mat | (49) |
| GNO3 | JN520693.1 | Guerrero Negro hypersaline microbial mat | (49) |
| GNO3 | JN520777.1 | Guerrero Negro hypersaline microbial mat | (49) |
| GNO3 | JN520808.1 | Guerrero Negro hypersaline microbial mat | (49) |
| GNO3 | JN520888.1 | Guerrero Negro hypersaline microbial mat | (49) |
| GNO3 | JN521296.1 | Guerrero Negro hypersaline microbial mat | (49) |
| GNO3 | JN521318.1 | Guerrero Negro hypersaline microbial mat | (49) |
| GNO3 | JN521412.1 | Guerrero Negro hypersaline microbial mat | (49) |
| GNO3 | JN521446.1 | Guerrero Negro hypersaline microbial mat | (49) |
| GNO3 | JN521478.1 | Guerrero Negro hypersaline microbial mat | (49) |
| GNO3 | JN521680.1 | Guerrero Negro hypersaline microbial mat | (49) |
| GNO3 | JN521773.1 | Guerrero Negro hypersaline microbial mat | (49) |
| GNO3 | JN521789.1 | Guerrero Negro hypersaline microbial mat | (49) |
| GNO3 | JN521795.1 | Guerrero Negro hypersaline microbial mat | (49) |
| GNO3 | JN521821.1 | Guerrero Negro hypersaline microbial mat | (49) |
| GNO3 | JN521855.1 | Guerrero Negro hypersaline microbial mat | (49) |
| GNO3 | JN522294.1 | Guerrero Negro hypersaline microbial mat | (49) |
| GNO3 | JN522431.1 | Guerrero Negro hypersaline microbial mat | (49) |
| GNO3 | JN522529.1 | Guerrero Negro hypersaline microbial mat | (49) |
| GNO3 | JN522562.1 | Guerrero Negro hypersaline microbial mat | (49) |
| GNO3 | JN522567.1 | Guerrero Negro hypersaline microbial mat | (49) |
| GNO3 | JN522706.1 | Guerrero Negro hypersaline microbial mat | (49) |
| GNO3 | JN522879.1 | Guerrero Negro hypersaline microbial mat | (49) |
| GNO3 | JN522906.1 | Guerrero Negro hypersaline microbial mat | (49) |
| GNO3 | JN523013.1 | Guerrero Negro hypersaline microbial mat | (49) |
| GNO3 | JN523022.1 | Guerrero Negro hypersaline microbial mat | (49) |
| GNO3 | JN523053.1 | Guerrero Negro hypersaline microbial mat | (49) |
| GNO3 | JN523102.1 | Guerrero Negro hypersaline microbial mat | (49) |
| GNO3 | JN523160.1 | Guerrero Negro hypersaline microbial mat | (49) |
| GNO3 | JN523183.1 | Guerrero Negro hypersaline microbial mat | (49) |
| GNO3 | JN523447.1 | Guerrero Negro hypersaline microbial mat | (49) |
| GNO3 | JN523536.1 | Guerrero Negro hypersaline microbial mat | (49) |
| GNO3 | JN523547.1 | Guerrero Negro hypersaline microbial mat | (49) |
| GNO3 | JN523552.1 | Guerrero Negro hypersaline microbial mat | (49) |
| GNO3 | JN523585.1 | Guerrero Negro hypersaline microbial mat | (49) |
| GNO3 | JN523628.1 | Guerrero Negro hypersaline microbial mat | (49) |
| GNO3 | JN523647.1 | Guerrero Negro hypersaline microbial mat | (49) |
| GNO3 | JN523651.1 | Guerrero Negro hypersaline microbial mat | (49) |
| GNO3 | JN523751.1 | Guerrero Negro hypersaline microbial mat | (49) |
| GNO3 | JN523771.1 | Guerrero Negro hypersaline microbial mat | (49) |
| GNO3 | JN523919.1 | Guerrero Negro hypersaline microbial mat | (49) |
| GNO3 | JN523926.1 | Guerrero Negro hypersaline microbial mat | (49) |
| GNO3 | JN523982.1 | Guerrero Negro hypersaline microbial mat | (49) |
| GNO3 | JN524277.1 | Guerrero Negro hypersaline microbial mat | (49) |
| GNO3 | JN524469.1 | Guerrero Negro hypersaline microbial mat | (49) |
| GNO3 | JN524547.1 | Guerrero Negro hypersaline microbial mat | (49) |
| GNO3 | JN524690.1 | Guerrero Negro hypersaline microbial mat | (49) |
| GNO3 | JN524784.1 | Guerrero Negro hypersaline microbial mat | (49) |
| GNO3 | JN524834.1 | Guerrero Negro hypersaline microbial mat | (49) |
| GNO3 | JN524909.1 | Guerrero Negro hypersaline microbial mat | (49) |
| GNO3 | JN524910.1 | Guerrero Negro hypersaline microbial mat | (49) |
| GNO3 | JN524989.1 | Guerrero Negro hypersaline microbial mat | (49) |
| GNO3 | JN525047.1 | Guerrero Negro hypersaline microbial mat | (49) |
| GNO3 | JN525057.1 | Guerrero Negro hypersaline microbial mat | (49) |
| GNO3 | JN525082.1 | Guerrero Negro hypersaline microbial mat | (49) |
| GNO3 | JN525090.1 | Guerrero Negro hypersaline microbial mat | (49) |
| GNO3 | JN525215.1 | Guerrero Negro hypersaline microbial mat | (49) |
| GNO3 | JN525260.1 | Guerrero Negro hypersaline microbial mat | (49) |
| GNO3 | JN525307.1 | Guerrero Negro hypersaline microbial mat | (49) |
| GNO3 | JN525336.1 | Guerrero Negro hypersaline microbial mat | (49) |
| GNO3 | JN525365.1 | Guerrero Negro hypersaline microbial mat | (49) |
| GNO3 | JN525470.1 | Guerrero Negro hypersaline microbial mat | (49) |
| GNO3 | JN525748.1 | Guerrero Negro hypersaline microbial mat | (49) |
| GNO3 | JN525804.1 | Guerrero Negro hypersaline microbial mat | (49) |
| GNO3 | JN525827.1 | Guerrero Negro hypersaline microbial mat | (49) |
| GNO3 | JN526008.1 | Guerrero Negro hypersaline microbial mat | (49) |
| GNO3 | JN526019.1 | Guerrero Negro hypersaline microbial mat | (49) |
| GNO3 | JN526023.1 | Guerrero Negro hypersaline microbial mat | (49) |
| GNO3 | JN526162.1 | Guerrero Negro hypersaline microbial mat | (49) |
| GNO3 | JN526188.1 | Guerrero Negro hypersaline microbial mat | (49) |
| GNO3 | JN526889.1 | Guerrero Negro hypersaline microbial mat | (49) |
| GNO3 | JN526943.1 | Guerrero Negro hypersaline microbial mat | (49) |
| GNO3 | JN527208.1 | Guerrero Negro hypersaline microbial mat | (49) |
| GNO3 | JN527461.1 | Guerrero Negro hypersaline microbial mat | (49) |
| GNO3 | JN527469.1 | Guerrero Negro hypersaline microbial mat | (49) |
| GNO3 | JN527490.1 | Guerrero Negro hypersaline microbial mat | (49) |
| GNO3 | JN527504.1 | Guerrero Negro hypersaline microbial mat | (49) |
| GNO3 | JN527742.1 | Guerrero Negro hypersaline microbial mat | (49) |
| GNO3 | JN527772.1 | Guerrero Negro hypersaline microbial mat | (49) |
| GNO3 | JN527848.1 | Guerrero Negro hypersaline microbial mat | (49) |
| GNO3 | JN527947.1 | Guerrero Negro hypersaline microbial mat | (49) |
| GNO3 | JN528042.1 | Guerrero Negro hypersaline microbial mat | (49) |
| GNO3 | JN528176.1 | Guerrero Negro hypersaline microbial mat | (49) |
| GNO3 | JN528256.1 | Guerrero Negro hypersaline microbial mat | (49) |
| GNO3 | JN529332.1 | Guerrero Negro hypersaline microbial mat | (49) |
| GNO3 | JN529423.1 | Guerrero Negro hypersaline microbial mat | (49) |
| GNO3 | JN529431.1 | Guerrero Negro hypersaline microbial mat | (49) |
| GNO3 | JN529603.1 | Guerrero Negro hypersaline microbial mat | (49) |
| GNO3 | JN529613.1 | Guerrero Negro hypersaline microbial mat | (49) |
| GNO3 | JN530038.1 | Guerrero Negro hypersaline microbial mat | (49) |
| GNO3 | JN530622.1 | Guerrero Negro hypersaline microbial mat | (49) |
| GNO3 | JN531195.1 | Guerrero Negro hypersaline microbial mat | (49) |
| GNO3 | JN531231.1 | Guerrero Negro hypersaline microbial mat | (49) |
| GNO3 | JN531398.1 | Guerrero Negro hypersaline microbial mat | (49) |
| GNO3 | JN531513.1 | Guerrero Negro hypersaline microbial mat | (49) |
| GNO3 | JN531675.1 | Guerrero Negro hypersaline microbial mat | (49) |
| GNO3 | JN532008.1 | Guerrero Negro hypersaline microbial mat | (49) |
| GNO3 | JN532213.1 | Guerrero Negro hypersaline microbial mat | (49) |
| GNO3 | JN532390.1 | Guerrero Negro hypersaline microbial mat | (49) |
| GNO3 | JN532748.1 | Guerrero Negro hypersaline microbial mat | (49) |
| GNO3 | JN533571.1 | Guerrero Negro hypersaline microbial mat | (49) |
| GNO3 | JN533948.1 | Guerrero Negro hypersaline microbial mat | (49) |
| GNO3 | JN534226.1 | Guerrero Negro hypersaline microbial mat | (49) |
| GNO3 | JN534405.1 | Guerrero Negro hypersaline microbial mat | (49) |
| GNO3 | JN534773.1 | Guerrero Negro hypersaline microbial mat | (49) |
| GNO3 | JN535973.1 | Guerrero Negro hypersaline microbial mat | (49) |
| GNO3 | JN535994.1 | Guerrero Negro hypersaline microbial mat | (49) |
| GNO3 | JN536230.1 | Guerrero Negro hypersaline microbial mat | (49) |
| GNO3 | JN536284.1 | Guerrero Negro hypersaline microbial mat | (49) |
| GNO3 | JN536716.1 | Guerrero Negro hypersaline microbial mat | (49) |
| GNO3 | JN537258.1 | Guerrero Negro hypersaline microbial mat | (49) |
| GNO3 | JN537384.1 | Guerrero Negro hypersaline microbial mat | (49) |
| GNO3 | JN537390.1 | Guerrero Negro hypersaline microbial mat | (49) |
| GNO3 | JN537507.1 | Guerrero Negro hypersaline microbial mat | (49) |
| GNO3 | JN537669.1 | Guerrero Negro hypersaline microbial mat | (49) |
| GNO3 | JN537906.1 | Guerrero Negro hypersaline microbial mat | (49) |
| GNO3 | JN538174.1 | Guerrero Negro hypersaline microbial mat | (49) |
| GNO3 | JN538196.1 | Guerrero Negro hypersaline microbial mat | (49) |
| GNO3 | JN538211.1 | Guerrero Negro hypersaline microbial mat | (49) |
| GNO3 | JN538754.1 | Guerrero Negro hypersaline microbial mat | (49) |
| GNO3 | JN539020.1 | Guerrero Negro hypersaline microbial mat | (49) |
| GNO3 | JN539308.1 | Guerrero Negro hypersaline microbial mat | (49) |
| GNO3 | JN539464.1 | Guerrero Negro hypersaline microbial mat | (49) |
| GNO3 | EF036307.1 | The roots and bulk sediment of the seagrass Zostera marina | (50) |
| GNO3 | FJ748805.1 | Pearl River Estuary sediments | (51) |
| GNO3 | GQ249615.1 | marine sediment: 18-20 cm layer | (52) |
| GNO3 | AB661565.1 | lake sediment | (53) |
| GNO3 | GU302492.1 | Marine sediments (900 m water depth, 0-24 m sediment depth) from Mississippi Canyon 118, northern slope of the Gulf of Mexico | (54) |
| GNO3 | DQ067004.1 | sediment of Lake Washington | (55) |
| GNO3 | EF687180.1 | Iron-oxidizing mat, Chefren mud volcano, Nile Deep Sea Fan, Eastern Mediterranean | (56) |
| GNO3 | EF687368.1 | Sulfide-oxidizing mat, Chefren mud volcano, Nile Deep Sea Fan, Eastern Mediterranean | (56) |
| GNO3 | EF687393.1 | Sulfide-oxidizing mat, Chefren mud volcano, Nile Deep Sea Fan, Eastern Mediterranean | (56) |
| GNO3 | AM745155.1 | marine sediments | (57) |
| GNO3 | HQ588522.1 | Amsterdam mud volcano sediment | (58) |
| GNO3 | GU584666.1 | marine hydrocarbon seep sediment | (59) |
| GNO3 | JN018949.1 | Deepwater Horizon oil spill/Gulf of Mexico | (60) |
| GNO3 | FJ484880.1 | wall biomat sample in El Zacaton at 4m depth | (61) |
| GNO3 | FN554086.1 | Logatchev hydrothermal vent field,Anya's Garden, watercolumn depth = 3038 m, sediment depth = 0-1 cm | (62) |
| GNO3 | EU265948.1 | Nitinat Lake at a depth of 20 m | (63) |
| GNO3 | EU265961.1 | Nitinat Lake at a depth of 20 m | (63) |
| GNO3 | FN549969.1 | Methane seep | (64) |
| GNO3 | FJ497306.1 | Fe-rich Mats and Basaltic Rock from Vailulu'u Seamount, American Samoa | (65) |
| GNO3 | EU478628.1 | Black Sea, 100 m | (66) |
| GNO3 | GQ850584.1 | bottom water in the northern Bering sea | (67) |
| GNO3 | EU734960.1 | Sediment bacteria in the northern Bering Sea | (68) |
| GNO3 | EU925876.1 | Sediment bacteria in the northern Bering Sea | (68) |
| GNO3 | EU925882.1 | Sediment bacteria in the northern Bering Sea | (68) |
| GNO3 | EF632751.1 | Aquatic environments of the high altitude Andean Altiplano (northern Chile) | Unpublished |
| GNO3 | AB240493.1 | Phragmites at Sosei River in Sappro, Japan | Unpublished |
| GNO3 | AB630662.1 | aquatic moss pillars | Unpublished |
| GNO3 | AB630663.1 | aquatic moss pillars | Unpublished |
| GNO3 | FJ717331.1 | marine sediment from Cullercoats | Unpublished |
| GNO3 | GQ246340.1 | North Yellow Sea sediments | Unpublished |
| GNO3 | GQ246450.1 | North Yellow Sea sediments | Unpublished |
| GNO3 | GQ433939.1 | seafloor Black FLOCS colonization experiment basalt grains | Unpublished |
| GNO3 | EU431759.1 | Calcium carbonate (moonmilk) muds at percolating waters where beetles feed | Unpublished |
| GNO3 | JN391703.1 | biofilm in anoxic tank of hybrid reactor | Unpublished |
| o CV106 | EU488395.1 | Siliciclastic sedment from Thalassia sea grass bed | (47) |
| o CV106 | HM272568.1 | skin, volar forearm" | (69) |
| o CV106 | DQ499326.1 | cave wall biofilms | (70) |
| o CV106 | JN579981.1 | soil | (71) |
| o CV106 | EU491440.1 | seafloor lavas from Hawai'i | (72) |
| o CV106 | EU491860.1 | seafloor lavas from Hawai'i | (72) |
| o CV106 | GU208269.1 | Dongping Lake sediment | (73) |
| o CV106 | GU119378.1 | reef water | (74) |
| o CV106 | GU119237.1 | reef water | (74) |
| o CV106 | EU734965.1 | sediment from station DBS1, northern Bering Sea | (68) |
| o CV106 | KC009975.1 | French Guiana coast | Unpublished |
| o CV106 | HM243942.1 | middle sediment from Honghu Lake | Unpublished |
| o CV106 | HM243986.1 | middle sediment from Honghu Lake | Unpublished |
| o CV106 | FJ484675.1 | wall biomat sample in El Zacaton at 17m depth | Unpublished |
| o CV106 | FJ902061.1 | biomat in the sediment of cenote LaPalita | Unpublished |
| o CV106 | KC009993.1 | French Guiana coast | Unpublished |
| o CV106 | KC010014.1 | French Guiana coast | Unpublished |
| o LD1-PA13 | EU135570.1 | Soil from an undisturbed mixed grass prairie preserve | (75) |
| o LD1-PA13 | AY114311.1 | anoxic marine sediment | (45) |
| o LD1-PA13 | FN396690.1 | Arctic marine surface sediment | (76) |
| o LD1-PA13 | JF320758.1 | Hydrothermal vent mat from Upper Lohiau vent site | (77) |
| o LD1-PA13 | JF320786.1 | hydrothermal vent mat from Upper North Hiolo vent site | (77) |
| o LD1-PA13 | AM935418.1 | pilot-scale bioremediation process of a hydrocarbon-contaminated soil | (78) |
| o LD1-PA13 | AM935176.1 | pilot-scale bioremediation process of a hydrocarbon-contaminated soil | (78) |
| o LD1-PA13 | AM936254.1 | pilot-scale bioremediation process of a hydrocarbon-contaminated soil | (78) |
| o LD1-PA13 | EF687454.1 | Nile Deep Sea Fan, Eastern Mediterranean | (56) |
| o LD1-PA13 | FJ712436.1 | Kazan mud volcano, East Mediterranean Sea | (58) |
| o LD1-PA13 | HQ588440.1 | Amsterdam mud volcano sediment | (58) |
| o LD1-PA13 | HQ588446.1 | Amsterdam mud volcano sediment | (58) |
| o LD1-PA13 | AM997855.1 | deep-sea surface sediments of the South Atlantic Ocean | (79) |
| o LD1-PA13 | FN553932.1 | Logatchev hydrothermal vent field | (62) |
| o LD1-PA13 | AM086134.1 | lake profundal sediment | (80) |
| o LD1-PA13 | AM086135.1 | lake profundal sediment | (80) |
| o LD1-PA13 | GQ472345.1 | lake sediment | (73) |
| o LD1-PA13 | EU925892.1 | sediment from station DBS1, northern Bering Sea | (68) |
| o LD1-PA13 | EU652629.1 | Yellow Sea sediment | Unpublished |
| o LD1-PA13 | JQ013346.1 | deep-sea sediment | Unpublished |
| o LD1-PA13 | JF775647.1 | grass carp (Ctenopharyngodon idellus) pond sediment | Unpublished |
| o LD1-PA13 | KC009986.1 | French Guiana coast | Unpublished |
| o LD1-PA13 | GU363029.1 | Marine sediment from the South China Sea | Unpublished |
| o LD1-PA13 | GU363057.1 | Marine sediment from the South China Sea | Unpublished |
| o LD1-PA13 | JX504495.1 | oolitic sands | Unpublished |
| o LD1-PA13 | JX504413.1 | oolitic sands | Unpublished |
| o LD1-PA13 | JX391644.1 | Surface Marine Sediments | Unpublished |
| o LD1-PA13 | HQ114187.1 | vermifilter system treated with continuous rural sewage | Unpublished |
| o LD1-PA13 | HM598262.1 | bacterial community in the sediments along a slope at South China Sea | Unpublished |
| o LD1-PA13 | JQ817352.1 | subseafloor sediment at the Formosa Ridge | Unpublished |
| o LD1-PA13 | JN886914.1 | sediments at the South West Indian Ridge | Unpublished |
| o LD1-PA13 | GQ356961.1 | methane seep sediment | Unpublished |
| o LD1-PA13 | HM243846.1 | middle sediment from Honghu Lake | Unpublished |
| o LD1-PA13 | HQ330558.1 | Lake Wivenhoe, Australia sediment | Unpublished |
| o LD1-PA13 | FJ545490.1 | North Yellow Sea sediment | Unpublished |
| o MSB-4E2 | FJ264784.1 | methane seep sediment | (41) |
| o MSB-4E2 | EU592418.1 | hypersaline sediment | (81) |
| o MSB-4E2 | EU487865.1 | Siliciclastic sedment from Thalassia sea grass bed | (47) |
| o MSB-4E2 | EU488301.1 | Siliciclastic sedment from Thalassia sea grass bed | (47) |
| o MSB-4E2 | EU488192.1 | Siliciclastic sedment from Thalassia sea grass bed | (47) |
| o MSB-4E2 | AB722255.1 | freshwater iron-rich microbial mat | (82) |
| o MSB-4E2 | HM228672.1 | riverine alluvial aquifers of the Mankyeong River | (83) |
| o MSB-4E2 | FJ712411.1 | Kazan Mud Volcano, Anaximander Mountains, East Mediterranean Sea | (58) |
| o MSB-4E2 | DQ811955.1 | mangrove soil | Unpublished |
| o MSB-4E2 | DQ811952.1 | mangrove soil | Unpublished |
| o MSB-4E2 | DQ811950.1 | mangrove soil | Unpublished |
| o MSB-4E2 | FJ484660.1 | wall biomat sample in El Zacaton at 17m depth | Unpublished |
| o MSB-4E2 | FJ484549.1 | wall biomat sample in El Zacaton at 17m depth | Unpublished |
| o MSB-4E2 | FJ716473.1 | Frasassi cave system, anoxic lake water | Unpublished |
| o MSB-4E2 | FJ484741.1 | wall biomat sample in El Zacaton at 17m depth | Unpublished |
| o MSB-4E2 | JF775619.1 | (Ctenopharyngodon idellus) pond sediment | Unpublished |
| o MSB-4E2 | GU982876.1 | marine sediment from the Western Pacific Ocean | Unpublished |
| o MSB-4E2 | JF422980.1 | 10 cm-deep methane seep sediment | Unpublished |
| o MSB-4E2 | HQ845891.1 | soil from coconut husk retting zone | Unpublished |
| o MSB-4E2 | FJ485078.1 | wall biomat sample in El Zacaton at 17m depth | Unpublished |
| o MSB-4E2 | FJ484664.1 | wall biomat sample in El Zacaton at 17m depth | Unpublished |
| o MSB-4E2 | FJ902124.1 | orange biomat sample from 8m deep in cenote Caracol | Unpublished |
| o SAW1 B6 | JN536934.1 | Guerrero Negro hypersaline microbial mat | (49) |
| o SAW1 B6 | JN531696.1 | Guerrero Negro hypersaline microbial mat | (49) |
| o SAW1 B6 | JN508675.1 | Guerrero Negro hypersaline microbial mat | (49) |
| o SAW1 B6 | JN472157.1 | Guerrero Negro hypersaline microbial mat | (49) |
| o SAW1 B6 | JN539634.1 | Guerrero Negro hypersaline microbial mat/hypersaline microbial mat | (49) |
| o SAW1 B6 | EU245112.1 | hypersaline microbial mat | (84) |
| o SAW1 B6 | EU245582.1 | Puerto Rico: Cabo Rojo, Candeleria lagoon | (84) |
| o SAW1 B6 | FJ712435.1 | Kazan Mud Volcano | (58) |
| o SAW1 B6 | HQ588533.1 | Amsterdam mud volcano sediment | (58) |
| o SAW1 B6 | HQ588531.1 | Amsterdam mud volcano sediment | (58) |
| o SAW1 B6 | HQ588580.1 | Amsterdam mud volcano sediment | (58) |
| o SAW1 B6 | FR851496.1 | permeable coral reef sands | (85) |
| o SAW1 B6 | GQ246422.1 | North Yellow Sea sediments | Unpublished |
| o SAW1 B6 | JN977151.1 | Jiaozhao Bay sediment | Unpublished |
| o SAW1 B6 | GU553724.1 | subseafloor sediment at the Yung-An Ridge | Unpublished |
| o SAW1 B6 | KC009974.1 | French Guiana coast | Unpublished |
| o SAW1 B6 | JQ816886.1 | subseafloor sediment at the Good Weather Ridge | Unpublished |
| o SAW1 B6 | JQ816895.1 | subseafloor sediment at the Good Weather Ridge | Unpublished |
| O sed1 PRR 10 | FJ351154.1 | Lake Pontchartrain | (86) |
| O sed1 PRR 10 | FJ479520.1 | Socompa Volcano, Puna de Atacama, Andes | (87) |
| O sed1 PRR 10 | FJ592732.1 | Socompa Volcano, Puna de Atacama, Andes | (87) |
| O sed1 PRR 10 | EF516273.1 | grassland soil | (88) |
| O sed1 PRR 10 | AJ390480.1 | bulk soil and rice roots of flooded rice microcosms | (43) |
| O sed1 PRR 10 | AJ390482.1 | bulk soil and rice roots of flooded rice microcosms | (43) |
| O sed1 PRR 10 | EU181953.1 | South China Sea | (89) |
| O sed1 PRR 10 | JQ366529.2 | FACE soil sample | (90) |
| O sed1 PRR 10 | JQ366538.2 | FACE soil sample | (90) |
| O sed1 PRR 10 | JQ366544.2 | FACE soil sample | (90) |
| O sed1 PRR 10 | JQ366826.2 | FACE soil sample | (90) |
| O sed1 PRR 10 | JQ366905.2 | FACE soil sample | (90) |
| O sed1 PRR 10 | JQ366909.2 | FACE soil sample | (90) |
| O sed1 PRR 10 | JQ366929.2 | FACE soil sample | (90) |
| O sed1 PRR 10 | JQ366966.2 | FACE soil sample | (90) |
| O sed1 PRR 10 | JQ366967.2 | FACE soil sample | (90) |
| O sed1 PRR 10 | JQ366968.2 | FACE soil sample | (90) |
| O sed1 PRR 10 | JQ366969.2 | FACE soil sample | (90) |
| O sed1 PRR 10 | JQ366970.2 | FACE soil sample | (90) |
| O sed1 PRR 10 | JQ366971.2 | FACE soil sample | (90) |
| O sed1 PRR 10 | JQ366972.2 | FACE soil sample | (90) |
| O sed1 PRR 10 | JQ366973.2 | FACE soil sample | (90) |
| O sed1 PRR 10 | JQ366974.2 | FACE soil sample | (90) |
| O sed1 PRR 10 | JQ367107.2 | FACE soil sample | (90) |
| O sed1 PRR 10 | JQ367380.2 | FACE soil sample | (90) |
| O sed1 PRR 10 | JQ367383.2 | FACE soil sample | (90) |
| O sed1 PRR 10 | JQ367419.2 | FACE soil sample | (90) |
| O sed1 PRR 10 | JQ367420.2 | FACE soil sample | (90) |
| O sed1 PRR 10 | KC604806.1 | groundwater | (91) |
| O sed1 PRR 10 | KC604813.1 | groundwater | (91) |
| O sed1 PRR 10 | GQ412818.1 | marine sediments | (92) |
| O sed1 PRR 10 | KC189791.1 | Wakulla Spring | (93) |
| O sed1 PRR 10 | AJ863173.1 | bulk soil | (94) |
| O sed1 PRR 10 | AJ863228.1 | bulk soil | (94) |
| O sed1 PRR 10 | GU269403.1 | bulk soil associated with the roots of Arachis hypogaea | (95) |
| O sed1 PRR 10 | JX120380.1 | subsurface aquifer sediment | (48) |
| O sed1 PRR 10 | EU335401.1 | soil aggregate | (96) |
| O sed1 PRR 10 | JN514162.1 | Guerrero Negro hypersaline microbial mat | (49) |
| O sed1 PRR 10 | AB656283.1 | rice paddy soil | (97) |
| O sed1 PRR 10 | AB656284.1 | rice paddy soil | (97) |
| O sed1 PRR 10 | AB656285.1 | rice paddy soil | (97) |
| O sed1 PRR 10 | AB656820.1 | rice paddy soil | (97) |
| O sed1 PRR 10 | AB656821.1 | rice paddy soil | (97) |
| O sed1 PRR 10 | AB656822.1 | rice paddy soil | (97) |
| O sed1 PRR 10 | AB656823.1 | rice paddy soil | (97) |
| O sed1 PRR 10 | AB656824.1 | rice paddy soil | (97) |
| O sed1 PRR 10 | AB656825.1 | rice paddy soil | (97) |
| O sed1 PRR 10 | AB656826.1 | rice paddy soil | (97) |
| O sed1 PRR 10 | AB656827.1 | rice paddy soil | (97) |
| O sed1 PRR 10 | AB656828.1 | rice paddy soil | (97) |
| O sed1 PRR 10 | AB656829.1 | rice paddy soil | (97) |
| O sed1 PRR 10 | AB656830.1 | rice paddy soil | (97) |
| O sed1 PRR 10 | AB657422.1 | rice paddy soil | (97) |
| O sed1 PRR 10 | AB657423.1 | rice paddy soil | (97) |
| O sed1 PRR 10 | AB658674.1 | rice paddy soil | (97) |
| O sed1 PRR 10 | AB659018.1 | rice paddy soil | (97) |
| O sed1 PRR 10 | AB659515.1 | rice paddy soil | (97) |
| O sed1 PRR 10 | AB659516.1 | rice paddy soil | (97) |
| O sed1 PRR 10 | AB659517.1 | rice paddy soil | (97) |
| O sed1 PRR 10 | AB659518.1 | rice paddy soil | (97) |
| O sed1 PRR 10 | AB660172.1 | rice paddy soil | (97) |
| O sed1 PRR 10 | AB660173.1 | rice paddy soil | (97) |
| O sed1 PRR 10 | AB660174.1 | rice paddy soil | (97) |
| O sed1 PRR 10 | AB660508.1 | rice paddy soil | (97) |
| O sed1 PRR 10 | AB660509.1 | rice paddy soil | (97) |
| O sed1 PRR 10 | AB660510.1 | rice paddy soil | (97) |
| O sed1 PRR 10 | AB661096.1 | rice paddy soil | (97) |
| O sed1 PRR 10 | AB661097.1 | rice paddy soil | (97) |
| O sed1 PRR 10 | JF145685.1 | skin | (69) |
| O sed1 PRR 10 | JN051306.1 | polluted aquifer | (98) |
| O sed1 PRR 10 | EF018381.1 | aspen rhizosphere | (99) |
| O sed1 PRR 10 | EF019236.1 | aspen rhizosphere | (99) |
| O sed1 PRR 10 | EF019249.1 | aspen rhizosphere | (99) |
| O sed1 PRR 10 | EF019550.1 | aspen rhizosphere | (99) |
| O sed1 PRR 10 | EF019905.1 | aspen rhizosphere | (99) |
| O sed1 PRR 10 | EF020005.1 | aspen rhizosphere | (99) |
| O sed1 PRR 10 | EF020076.1 | aspen rhizosphere | (99) |
| O sed1 PRR 10 | HM185859.1 | Hanford Site subsurface sediment | (39) |
| O sed1 PRR 10 | HM186233.1 | Hanford Site subsurface sediment | (39) |
| O sed1 PRR 10 | HM186564.1 | Hanford Site subsurface sediment | (39) |
| O sed1 PRR 10 | HM186680.1 | Hanford Site subsurface sediment | (39) |
| O sed1 PRR 10 | HM186746.1 | Hanford Site subsurface sediment | (39) |
| O sed1 PRR 10 | HM186757.1 | Hanford Site subsurface sediment | (39) |
| O sed1 PRR 10 | HM187008.1 | Hanford Site subsurface sediment | (39) |
| O sed1 PRR 10 | HM187178.1 | Hanford Site subsurface sediment | (39) |
| O sed1 PRR 10 | HM187207.1 | Hanford Site subsurface sediment | (39) |
| O sed1 PRR 10 | HM187275.1 | Hanford Site subsurface sediment | (39) |
| O sed1 PRR 10 | AM935797.1 | hydrocarbon-contaminated soil | (78) |
| O sed1 PRR 10 | DQ431883.1 | Gulf of Mexico sediment | (100) |
| O sed1 PRR 10 | DQ067003.1 | sediment of Lake Washington | (55) |
| O sed1 PRR 10 | AY874110.2 | National Park, Taiwan | (101) |
| O sed1 PRR 10 | JN187544.1 | soil | (71) |
| O sed1 PRR 10 | JN580047.1 | soil | (71) |
| O sed1 PRR 10 | JF341310.1 | concrete sewer biofilm | (102) |
| O sed1 PRR 10 | GQ287562.1 | India: Pinadri Glacier, Himalayas | (103) |
| O sed1 PRR 10 | FN659285.1 | earthworm gut content | (104) |
| O sed1 PRR 10 | EU875575.1 | activated sludge | (105) |
| O sed1 PRR 10 | FJ478598.1 | tall grass prairie | (40) |
| O sed1 PRR 10 | FJ478843.1 | tall grass prairie | (40) |
| O sed1 PRR 10 | FJ478864.1 | tall grass prairie | (40) |
| O sed1 PRR 10 | FJ479218.1 | tall grass prairie | (40) |
| O sed1 PRR 10 | FJ479480.1 | tall grass prairie | (40) |
| O sed1 PRR 10 | JX967628.1 | soil | (106) |
| O sed1 PRR 10 | GQ302574.1 | cold spring | (107) |
| O sed1 PRR 10 | JN868140.1 | sediment of Lake Zixia | (108) |
| O sed1 PRR 10 | JQ795212.1 | rhizosphere of Ceratophyllum demersum | (109) |
| O sed1 PRR 10 | JQ795340.1 | rhizosphere of Vallisneria natans | (109) |
| O sed1 PRR 10 | JN896937.1 | Tattapani geothermal spring in Himachal Pradesh | Unpublished |
| O sed1 PRR 10 | JQ663704.1 | soil and groundwater | Unpublished |
| O sed1 PRR 10 | JX080257.1 | gas field soil sample | Unpublished |
| O sed1 PRR 10 | AJ582051.1 | soil sample from a uranium mining waste | Unpublished |
| O sed1 PRR 10 | GU911391.1 | bottom sediments Lake Baikal sites of natural oil seeps | Unpublished |
| O sed1 PRR 10 | HQ891192.1 | sediments containing oil | Unpublished |
| O sed1 PRR 10 | EF032778.1 | cyanobacterial mat in Hawaii Volcanoes National Park lava cave | Unpublished |
| O sed1 PRR 10 | FJ205378.1 | deep marine sediments, depth:2725m | Unpublished |
| O sed1 PRR 10 | GU363007.1 | marine sediment from the South China Sea | Unpublished |
| O sed1 PRR 10 | HM598252.1 | the surface sediment near the northern continental marginal slope of Xisha Trough, China Sea | Unpublished |
| O sed1 PRR 10 | JX391273.1 | marine sediment | Unpublished |
| O sed1 PRR 10 | AM991194.1 | karst spring water | Unpublished |
| O sed1 PRR 10 | GQ500696.1 | Mammoth Cave Karst Aquifers | Unpublished |
| O sed1 PRR 10 | GQ500705.1 | Mammoth Cave Karst Aquifers | Unpublished |
| O sed1 PRR 10 | GQ860107.1 | PCB-Spiked Ohio River sediments | Unpublished |
| O sed1 PRR 10 | GQ860129.1 | PCB-Spiked Ohio River sediments | Unpublished |
| O sed1 PRR 10 | GQ860291.1 | Ohio River sediments | Unpublished |
| O sed1 PRR 10 | GU325865.1 | PCB-Spiked Ohio River sediments | Unpublished |
| O sed1 PRR 10 | GU325878.1 | PCB-Spiked Ohio River sediments | Unpublished |
| O sed1 PRR 10 | HM050538.1 | eutrophic shallow lakes | Unpublished |
| O sed1 PRR 10 | HM050566.1 | eutrophic shallow lakes | Unpublished |
| O sed1 PRR 10 | HM243794.1 | upper sediment from Honghu Lake | Unpublished |
| O sed1 PRR 10 | HM243853.1 | middle sediment from Honghu Lake | Unpublished |
| O sed1 PRR 10 | HQ143828.1 | carbonate-rich lake in British Columbia, Canada | Unpublished |
| O sed1 PRR 10 | HQ143829.1 | carbonate-rich lake in British Columbia, Canada | Unpublished |
| O sed1 PRR 10 | JF420679.1 | glacier sediment | Unpublished |
| O sed1 PRR 10 | JN832632.1 | bottom sediments of Cape Gorevoy Utes of Lake Baikal | Unpublished |
| O sed1 PRR 10 | JN832640.1 | bottom sediments of Cape Gorevoy Utes of Lake Baikal | Unpublished |
| O sed1 PRR 10 | JN832641.1 | bottom sediments of Cape Gorevoy Utes of Lake Baikal | Unpublished |
| O sed1 PRR 10 | JN832644.1 | bottom sediments of Cape Gorevoy Utes of Lake Baikal | Unpublished |
| O sed1 PRR 10 | JN832658.1 | bottom sediments of Cape Gorevoy Utes of Lake Baikal | Unpublished |
| O sed1 PRR 10 | JN832662.1 | bottom sediments of Cape Gorevoy Utes of Lake Baikal | Unpublished |
| O sed1 PRR 10 | JN832663.1 | bottom sediments of Cape Gorevoy Utes of Lake Baikal | Unpublished |
| O sed1 PRR 10 | JN832666.1 | bottom sediments of Cape Gorevoy Utes of Lake Baikal | Unpublished |
| O sed1 PRR 10 | JQ807883.1 | Lake Baikal, jelly-like biofilms | Unpublished |
| O sed1 PRR 10 | JQ807888.1 | Lake Baikal, jelly-like biofilms | Unpublished |
| O sed1 PRR 10 | AB568071.1 | wastewater treatment plant | Unpublished |
| O sed1 PRR 10 | AJ306763.1 | Bioreactor | Unpublished |
| O sed1 PRR 10 | HQ114087.1 | vermifilter system treated with continuous rural sewage | Unpublished |
| O sed1 PRR 10 | HQ114110.1 | vermifilter system treated with continuous rural sewage | Unpublished |
| O sed1 PRR 10 | HQ114156.1 | vermifilter system treated with continuous rural sewage | Unpublished |
| O sed1 PRR 10 | HQ114191.1 | vermifilter system treated with continuous rural sewage | Unpublished |
| O sed1 PRR 10 | AB240259.1 | Rhizosphere biofilm bulk soil of reed bed reactor in the laboratory | Unpublished |
| O sed1 PRR 10 | AB240377.1 | Rhizosphere biofilm bulk soil of reed bed reactor in the laboratory | Unpublished |
| O sed1 PRR 10 | AB426188.1 | lotus field soil | Unpublished |
| O sed1 PRR 10 | DQ093907.1 | rhizosphere | Unpublished |
| O sed1 PRR 10 | EU714504.1 | rhizosphere soil | Unpublished |
| O sed1 PRR 10 | EF662797.1 | soil microbial community | Unpublished |
| O sed1 PRR 10 | EF662837.1 | soil microbial community | Unpublished |
| O sed1 PRR 10 | EU132837.1 | soil from an undisturbed mixed grass prairie preserve | Unpublished |
| O sed1 PRR 10 | EU135563.1 | soil from an undisturbed mixed grass prairie preserve | Unpublished |
| O sed1 PRR 10 | EU135564.1 | soil from an undisturbed mixed grass prairie preserve | Unpublished |
| O sed1 PRR 10 | EU135565.1 | soil from an undisturbed mixed grass prairie preserve | Unpublished |
| O sed1 PRR 10 | EU135566.1 | soil from an undisturbed mixed grass prairie preserve | Unpublished |
| O sed1 PRR 10 | EU135569.1 | soil from an undisturbed mixed grass prairie preserve | Unpublished |
| O sed1 PRR 10 | JF833783.1 | potassium mine soil | Unpublished |
| O sed1 PRR 10 | JF833822.1 | potassium mine soil | Unpublished |
| O sed1 PRR 10 | JF833858.1 | potassium mine soil | Unpublished |
| O sed1 PRR 10 | JN038631.1 | Chongxi wetland soil | Unpublished |
| O sed1 PRR 10 | JN038633.1 | Chongxi wetland soil | Unpublished |
| O sed1 PRR 10 | JN038717.1 | Chongxi wetland soil | Unpublished |
| O sed1 PRR 10 | JN038791.1 | Chongxi wetland soil | Unpublished |
| O sed1 PRR 10 | JN417574.1 | Soil | Unpublished |
| O sed1 PRR 10 | JN854342.1 | field soil | Unpublished |
| O sed1 PRR 10 | JN855270.1 | field soil | Unpublished |
| O sed1 PRR 10 | JQ696461.1 | soil from Tet watersheds | Unpublished |
| O Sed1 Unclass | JQ580002.1 | sediments from Figueiras Beach | (110) |
| O Sed1 Unclass | FJ355165.1 | Lake Charles, floodwater | (86) |
| O Sed1 Unclass | FJ355166.1 | Lake Charles, floodwater | (86) |
| O Sed1 Unclass | EU234541.1 | surface applied Illinois River sediment | (111) |
| O Sed1 Unclass | EU234557.1 | surface applied Illinois River sediment | (111) |
| O Sed1 Unclass | DQ822228.1 | soil microcosm | (112) |
| O Sed1 Unclass | AJ390458.1 | bulk soil and rice roots of flooded rice microcosms | (43) |
| O Sed1 Unclass | EU181735.1 | South China Sea | (89) |
| O Sed1 Unclass | JQ366672.2 | FACE soil sample | (90) |
| O Sed1 Unclass | JQ366910.2 | FACE soil sample | (90) |
| O Sed1 Unclass | JQ367434.2 | FACE soil sample | (90) |
| O Sed1 Unclass | JQ367435.2 | FACE soil sample | (90) |
| O Sed1 Unclass | JQ367436.2 | FACE soil sample | (90) |
| O Sed1 Unclass | EU135561.1 | soil from an undisturbed mixed grass prairie preserve | (75) |
| O Sed1 Unclass | EU135571.1 | soil from an undisturbed mixed grass prairie preserve | (75) |
| O Sed1 Unclass | EU135573.1 | soil from an undisturbed mixed grass prairie preserve | (75) |
| O Sed1 Unclass | EU135575.1 | soil from an undisturbed mixed grass prairie preserve | (75) |
| O Sed1 Unclass | EU135578.1 | soil from an undisturbed mixed grass prairie preserve | (75) |
| O Sed1 Unclass | JQ217754.1 | mine water from gossan | (113) |
| O Sed1 Unclass | KC604930.1 | groundwater | (91) |
| O Sed1 Unclass | KC605085.1 | groundwater | (91) |
| O Sed1 Unclass | AY114325.1 | anoxic marine sediment | (45) |
| O Sed1 Unclass | DQ351773.1 | heavy metal contaminated marine sediments | (114) |
| O Sed1 Unclass | HM185965.1 | Hanford Site subsurface sediment | (114) |
| O Sed1 Unclass | HM186434.1 | Hanford Site subsurface sediment | (114) |
| O Sed1 Unclass | HM186643.1 | Hanford Site subsurface sediment | (114) |
| O Sed1 Unclass | HM186672.1 | Hanford Site subsurface sediment | (114) |
| O Sed1 Unclass | HM186678.1 | Hanford Site subsurface sediment | (114) |
| O Sed1 Unclass | HM186890.1 | Hanford Site subsurface sediment | (114) |
| O Sed1 Unclass | EU487980.1 | siliciclastic sedment from Thalassia sea grass bed | (47) |
| O Sed1 Unclass | EU488041.1 | siliciclastic sedment from Thalassia sea grass bed | (47) |
| O Sed1 Unclass | EU488298.1 | siliciclastic sedment from Thalassia sea grass bed | (47) |
| O Sed1 Unclass | EU488324.1 | siliciclastic sedment from Thalassia sea grass bed | (47) |
| O Sed1 Unclass | EU117058.1 | rhizosphere soil | (115) |
| O Sed1 Unclass | JN515170.1 | Guerrero Negro hypersaline microbial mat | (49) |
| O Sed1 Unclass | FR732395.1 | fen soil microcosm | (116) |
| O Sed1 Unclass | EU245606.1 | Puerto Rico: Cabo Rojo, Candeleria lagoon | (84) |
| O Sed1 Unclass | AB288605.1 | Rice paddy soil | (117) |
| O Sed1 Unclass | AB656286.1 | Rice paddy soil | (117) |
| O Sed1 Unclass | AB656287.1 | Rice paddy soil | (117) |
| O Sed1 Unclass | AB656288.1 | Rice paddy soil | (117) |
| O Sed1 Unclass | AB656289.1 | Rice paddy soil | (117) |
| O Sed1 Unclass | AB657424.1 | Rice paddy soil | (97) |
| O Sed1 Unclass | AB658106.1 | Rice paddy soil | (97) |
| O Sed1 Unclass | AB658108.1 | Rice paddy soil | (97) |
| O Sed1 Unclass | AB658109.1 | Rice paddy soil | (97) |
| O Sed1 Unclass | AB658110.1 | Rice paddy soil | (97) |
| O Sed1 Unclass | AB658669.1 | Rice paddy soil | (97) |
| O Sed1 Unclass | AB658670.1 | Rice paddy soil | (97) |
| O Sed1 Unclass | AB658672.1 | Rice paddy soil | (97) |
| O Sed1 Unclass | AB658673.1 | Rice paddy soil | (97) |
| O Sed1 Unclass | AB659015.1 | Rice paddy soil | (97) |
| O Sed1 Unclass | AB659016.1 | Rice paddy soil | (97) |
| O Sed1 Unclass | AB659017.1 | Rice paddy soil | (97) |
| O Sed1 Unclass | AB659019.1 | Rice paddy soil | (97) |
| O Sed1 Unclass | AB659513.1 | Rice paddy soil | (97) |
| O Sed1 Unclass | AB659514.1 | Rice paddy soil | (97) |
| O Sed1 Unclass | AB661098.1 | Rice paddy soil | (97) |
| O Sed1 Unclass | AB629236.1 | deep-sea hydrothermal field | (118) |
| O Sed1 Unclass | EF208642.1 | sandy carbonate sediment | (119) |
| O Sed1 Unclass | AF280852.1 | pharmaceutical wastewater | (120) |
| O Sed1 Unclass | EF019721.1 | aspen rhizosphere | (99) |
| O Sed1 Unclass | EF019928.1 | aspen rhizosphere | (99) |
| O Sed1 Unclass | AM935389.1 | hydrocarbon-contaminated soil | (78) |
| O Sed1 Unclass | JF341451.1 | concrete sewer biofilm | (102) |
| O Sed1 Unclass | DQ123706.1 | PAH-contaminated soil | (121) |
| O Sed1 Unclass | DQ123712.1 | PAH-contaminated soil | (121) |
| O Sed1 Unclass | DQ123725.1 | PAH-contaminated soil | (121) |
| O Sed1 Unclass | DQ123735.1 | PAH-contaminated soil | (121) |
| O Sed1 Unclass | GQ261283.1 | sediment of Dongping Lake | (73) |
| O Sed1 Unclass | AY921903.1 | farm soil | (122) |
| O Sed1 Unclass | FJ478933.1 | soil from an undisturbed mixed grass prairie preserve | (40) |
| O Sed1 Unclass | FJ479290.1 | soil from an undisturbed mixed grass prairie preserve | (40) |
| O Sed1 Unclass | FR871445.1 | greenhouse soil | Unpublished |
| O Sed1 Unclass | DQ256527.1 | Great Barrier Reef calcareous sediments | Unpublished |
| O Sed1 Unclass | DQ444035.1 | benzene-like compounds on the structure of microbial community in Songhuajiang River sediments | Unpublished |
| O Sed1 Unclass | DQ444129.1 | benzene-like compounds on the structure of microbial community in Songhuajiang River sediments | Unpublished |
| O Sed1 Unclass | EF393473.1 | Ohio River sediments: PCB-spiked | Unpublished |
| O Sed1 Unclass | JN873930.1 | Hydrothermal plumes | Unpublished |
| O Sed1 Unclass | JN873945.1 | Hydrothermal plumes | Unpublished |
| O Sed1 Unclass | EU617821.1 | Yellow Sea sediment | Unpublished |
| O Sed1 Unclass | FJ268521.1 | marine sediment in the oxygen minimum zone (1000 m depth) | Unpublished |
| O Sed1 Unclass | FJ813576.1 | marine sediment | Unpublished |
| O Sed1 Unclass | HM598177.1 | sediments along a slope at South China Sea | Unpublished |
| O Sed1 Unclass | JQ925128.1 | cold seep sediments | Unpublished |
| O Sed1 Unclass | FJ716426.1 | Frasassi cave system, anoxic lakewater | Unpublished |
| O Sed1 Unclass | GQ500702.1 | Mammoth Cave Karst Aquifers | Unpublished |
| O Sed1 Unclass | GQ860162.1 | Ohio River sediments | Unpublished |
| O Sed1 Unclass | GU127037.1 | anoxic zone from hydropower plant reservoir | Unpublished |
| O Sed1 Unclass | GU127059.1 | anoxic zone from hydropower plant reservoir | Unpublished |
| O Sed1 Unclass | GU325852.1 | Ohio River sediments | Unpublished |
| O Sed1 Unclass | GU325946.1 | Ohio River sediments | Unpublished |
| O Sed1 Unclass | GU325958.1 | Ohio River sediments | Unpublished |
| O Sed1 Unclass | JF728142.1 | Lake Kinneret | Unpublished |
| O Sed1 Unclass | JQ738939.1 | Lonar sediment surface rocks | Unpublished |
| O Sed1 Unclass | KC541115.1 | river sediment | Unpublished |
| O Sed1 Unclass | HQ114146.1 | vermifilter system treated with continuous rural sewage | Unpublished |
| O Sed1 Unclass | DQ093906.1 | rhizosphere | Unpublished |
| O Sed1 Unclass | JN409200.1 | rhizosphere soil of cucumber | Unpublished |
| O Sed1 Unclass | JX519121.1 | rhizosphere soil | Unpublished |
| O Sed1 Unclass | FJ484481.1 | wall biomat sample in El Zacaton at 17m depth | Unpublished |
| O Sed1 Unclass | FJ484527.1 | wall biomat sample in El Zacaton at 17m depth | Unpublished |
| O Sed1 Unclass | FJ485054.1 | wall biomat sample in El Zacaton at 17m depth | Unpublished |
| O Sed1 Unclass | FJ902045.1 | phreatic limestone sinkholes | Unpublished |
| O Sed1 Unclass | FJ902356.1 | phreatic limestone sinkholes | Unpublished |
| O Sed1 Unclass | DQ128791.2 | agricultural and forest soils | Unpublished |
| O Sed1 Unclass | EU881351.1 | maize-sweet potato cropland soil | Unpublished |
| O Sed1 Unclass | GU444093.1 | cotton straw treated soil | Unpublished |
| O Sed1 Unclass | JF772749.1 | rice paddy soil | Unpublished |
| O Sed1 Unclass | JN038588.1 | Chongxi wetland soil | Unpublished |
| O Sed1 Unclass | JX415401.1 | bulk soil | Unpublished |
| O Sed1 Unclass | JX415422.1 | bulk soil | Unpublished |
| o wb1 H11 | HQ849837.1 | marine sediments of the South China Sea | (89) |
| o wb1 H11 | JQ367182.2 | FACE soil sample | (90) |
| o wb1 H11 | FM956225.1 | rice field soil | (123) |
| o wb1 H11 | AB657425.1 | rice field soil | (123) |
| o wb1 H11 | AF317770.1 | Nullarbor caves, Australia | (124) |
| o wb1 H11 | EU438307.1 | marine sediments | (125) |
| o wb1 H11 | EF205586.1 | geothermal spring mat | (126) |
| o wb1 H11 | GU208350.1 | sediments of the shallow Lake | (73) |
| o wb1 H11 | GQ259301.1 | seawater and sediments from an arctic fjord of svalbard | (127) |
| o wb1 H11 | FJ358927.1 | marine reef sandy sediment | Unpublished |
| o wb1 H11 | HQ684407.1 | forest soil in Orchid Island | Unpublished |
| o wb1 H11 | HM445347.1 | white microbial mat from lava tube walls | Unpublished |
| o wb1 H11 | JN643037.1 | tan-red microbial mat from lava tube wall | Unpublished |
| o wb1 H11 | JN051339.1 | polluted aquifer | Unpublished |
| o wb1 H11 | AB426197.1 | lotus field soil | Unpublished |
| o wb1 H11 | EU652628.1 | Yellow Sea sediment | Unpublished |
| o wb1 H11 | KC009950.1 | French Guiana coast | Unpublished |
| o wb1 H11 | KC009967.1 | French Guiana coast | Unpublished |
| o_SSS58A | EU592470.1 | the Salton Sea | (81) |
| o_SSS58A | EF459830.1 | Baltic Sea sediment | (128) |
| o_SSS58A | EF459880.1 | Baltic Sea sediment | (128) |
| o_SSS58A | EU487903.1 | siliciclastic sedment from Thalassia sea grass bed | (47) |
| o_SSS58A | EU487957.1 | siliciclastic sedment from Thalassia sea grass bed | (47) |
| o_SSS58A | JN444823.1 | Guerrero Negro hypersaline microbial mat | (49) |
| o_SSS58A | JN449198.1 | Guerrero Negro hypersaline microbial mat | (49) |
| o_SSS58A | JN457471.1 | Guerrero Negro hypersaline microbial mat | (49) |
| o_SSS58A | JN457584.1 | Guerrero Negro hypersaline microbial mat | (49) |
| o_SSS58A | JN457637.1 | Guerrero Negro hypersaline microbial mat | (49) |
| o_SSS58A | JN501394.1 | Guerrero Negro hypersaline microbial mat | (49) |
| o_SSS58A | JN506477.1 | Guerrero Negro hypersaline microbial mat | (49) |
| o_SSS58A | JN508495.1 | Guerrero Negro hypersaline microbial mat | (49) |
| o_SSS58A | JN508970.1 | Guerrero Negro hypersaline microbial mat | (49) |
| o_SSS58A | JN512966.1 | Guerrero Negro hypersaline microbial mat | (49) |
| o_SSS58A | JN513619.1 | Guerrero Negro hypersaline microbial mat | (49) |
| o_SSS58A | JN514936.1 | Guerrero Negro hypersaline microbial mat | (49) |
| o_SSS58A | JN516498.1 | Guerrero Negro hypersaline microbial mat | (49) |
| o_SSS58A | JN516692.1 | Guerrero Negro hypersaline microbial mat | (49) |
| o_SSS58A | JN519173.1 | Guerrero Negro hypersaline microbial mat | (49) |
| o_SSS58A | JN520609.1 | Guerrero Negro hypersaline microbial mat | (49) |
| o_SSS58A | JN521058.1 | Guerrero Negro hypersaline microbial mat | (49) |
| o_SSS58A | JN523762.1 | Guerrero Negro hypersaline microbial mat | (49) |
| o_SSS58A | JN523818.1 | Guerrero Negro hypersaline microbial mat | (49) |
| o_SSS58A | JN526440.1 | Guerrero Negro hypersaline microbial mat | (49) |
| o_SSS58A | JN526805.1 | Guerrero Negro hypersaline microbial mat | (49) |
| o_SSS58A | JN527309.1 | Guerrero Negro hypersaline microbial mat | (49) |
| o_SSS58A | JN527328.1 | Guerrero Negro hypersaline microbial mat | (49) |
| o_SSS58A | JN527931.1 | Guerrero Negro hypersaline microbial mat | (49) |
| o_SSS58A | JN530484.1 | Guerrero Negro hypersaline microbial mat | (49) |
| o_SSS58A | JN531373.1 | Guerrero Negro hypersaline microbial mat | (49) |
| o_SSS58A | JN531689.1 | Guerrero Negro hypersaline microbial mat | (49) |
| o_SSS58A | JN532577.1 | Guerrero Negro hypersaline microbial mat | (49) |
| o_SSS58A | JN532855.1 | Guerrero Negro hypersaline microbial mat | (49) |
| o_SSS58A | JN533354.1 | Guerrero Negro hypersaline microbial mat | (49) |
| o_SSS58A | JN533497.1 | Guerrero Negro hypersaline microbial mat | (49) |
| o_SSS58A | JN534624.1 | Guerrero Negro hypersaline microbial mat | (49) |
| o_SSS58A | JN535814.1 | Guerrero Negro hypersaline microbial mat | (49) |
| o_SSS58A | JN537472.1 | Guerrero Negro hypersaline microbial mat | (49) |
| o_SSS58A | JN538675.1 | Guerrero Negro hypersaline microbial mat | (49) |
| o_SSS58A | JN539037.1 | Guerrero Negro hypersaline microbial mat | (49) |
| o_SSS58A | JN539471.1 | Guerrero Negro hypersaline microbial mat | (49) |
| o_SSS58A | JN539750.1 | Guerrero Negro hypersaline microbial mat | (49) |
| o_SSS58A | FN396641.1 | Arctic marine surface sediment | (76) |
| o_SSS58A | FN396645.1 | Arctic marine surface sediment | (76) |
| o_SSS58A | EF999394.1 | Pearl River Estuary sediments at 50cm | (51) |
| o_SSS58A | HM186666.1 | Hanford Site subsurface sediment | (39) |
| o_SSS58A | DQ521801.1 | hypersaline Gulf of Mexico sediments | (129) |
| o_SSS58A | DQ521802.1 | hypersaline Gulf of Mexico sediments | (129) |
| o_SSS58A | GU302489.1 | Gulf of Mexico hydrocarbon seep | (54) |
| o_SSS58A | AB305416.1 | thermal and geochemical gradients in hydrothermal sediments at the Yonaguni Knoll IV hydrothermal field in the Southern Okinawa trough | (130) |
| o_SSS58A | AB305537.1 | thermal and geochemical gradients in hydrothermal sediments at the Yonaguni Knoll IV hydrothermal field in the Southern Okinawa trough | (130) |
| o_SSS58A | FJ712468.1 | Kazan Mud Volcano, Anaximander Mountains, East Mediterranean Sea | (58) |
| o_SSS58A | HQ588557.1 | Amsterdam mud volcano sediment | (58) |
| o_SSS58A | AY133365.1 | coastal Antarctic sediment | (131) |
| o_SSS58A | AM181877.1 | Hypoliminion sediments of Lake Kinneret (Israel) | (132) |
| o_SSS58A | GU236082.1 | uranium-mining site | (133) |
| o_SSS58A | HM346697.1 | waterfowl lake sediment in Beijing Zoo | (134) |
| o_SSS58A | JN805704.1 | Fresh water sediment | (135) |
| o_SSS58A | KC922464.1 | groundwater discharge zone sediment | Unpublished |
| o_SSS58A | AB250558.1 | Isolated form the “Grotta Azzura” of Cape Palinuro (Salerno, Italy) | Unpublished |
| o_SSS58A | GU553780.1 | subseafloor sediment at the Yung-An Ridge | Unpublished |
| o_SSS58A | GU982860.1 | Marine Sediment from the Western Pacific Ocean | Unpublished |
| o_SSS58A | JX000661.1 | subseafloor sediment at the Fangliao Ridge | Unpublished |
| o_SSS58A | JF495345.1 | sediment from anoxic fjord | Unpublished |
| o_SSS58A | HF922342.1 | high pressure reactor | Unpublished |
| o_SSS58A | FJ484438.1 | wall biomat sample in El Zacaton at 17m depth | Unpublished |
| o_SSS58A | FJ484447.1 | wall biomat sample in El Zacaton at 17m depth | Unpublished |
| o_SSS58A | FJ485035.1 | wall biomat sample in El Zacaton at 17m depth | Unpublished |
| o_SSS58A | HM480237.1 | atoll of Kiritimati, Republic of Kiribati | Unpublished |
| o_SSS58A | HM480238.1 | atoll of Kiritimati, Republic of Kiribati | Unpublished |
| o_SSS58A | HM480239.1 | atoll of Kiritimati, Republic of Kiribati | Unpublished |
| o_SSS58A | JQ739102.1 | Lonar sediment surface rocks | Unpublished |
| PBSIII | JQ580294.1 | Sediments from Rodas Beach polluted with crude oil | (110) |
| PBSIII | JQ580360.1 | Sediments from Rodas Beach polluted with crude oil | (110) |
| PBSIII | JN868209.1 | Sediments from Rodas Beach polluted with crude oil | (110) |
| PBSIII | FJ351465.1 | Lake Pontchartrain, Transect 2 Station 2, sediment | (86) |
| PBSIII | AF424372.1 | Antarctic continental shelf sediment | (136) |
| PBSIII | AF424430.1 | Antarctic continental shelf sediment | (136) |
| PBSIII | DQ125879.1 | uranium contaminated soil | (137) |
| PBSIII | AJ390450 | uncultured soil bacterium PBS-III-9 | (43) |
| PBSIII | AF050547.1 | Hydrocarbon- and chlorinated-solvent-contaminated aquifer undergoing intrinsic bioremediation | (138) |
| PBSIII | JQ366533.2 | FACE soil sample | (90) |
| PBSIII | JQ366828.2 | FACE soil sample | (90) |
| PBSIII | FJ746129.1 | ocean sediment, 5306 m water depth | (139) |
| PBSIII | AM490689.1 | SA:Wyoming, Lower Kane Cave | (140) |
| PBSIII | HQ003610.1 | Carrizo shallow lake, Brakish Lake | (141) |
| PBSIII | HQ003611.1 | Carrizo shallow lake, Brakish Lake | (141) |
| PBSIII | KC604767.1 | groundwater | (91) |
| PBSIII | KC605005.1 | groundwater | (91) |
| PBSIII | EU487907.1 | siliciclastic sedment from Thalassia sea grass bed | (47) |
| PBSIII | JN427143.1 | Guerrero Negro hypersaline microbial mat | (49) |
| PBSIII | JN427543.1 | Guerrero Negro hypersaline microbial mat | (49) |
| PBSIII | JN429192.1 | Guerrero Negro hypersaline microbial mat | (49) |
| PBSIII | JN429594.1 | Guerrero Negro hypersaline microbial mat | (49) |
| PBSIII | JN429641.1 | Guerrero Negro hypersaline microbial mat | (49) |
| PBSIII | JN433482.1 | Guerrero Negro hypersaline microbial mat | (49) |
| PBSIII | JN433483.1 | Guerrero Negro hypersaline microbial mat | (49) |
| PBSIII | JN437655.1 | Guerrero Negro hypersaline microbial mat | (49) |
| PBSIII | JN438597.1 | Guerrero Negro hypersaline microbial mat | (49) |
| PBSIII | JN439121.1 | Guerrero Negro hypersaline microbial mat | (49) |
| PBSIII | JN439317.1 | Guerrero Negro hypersaline microbial mat | (49) |
| PBSIII | JN439445.1 | Guerrero Negro hypersaline microbial mat | (49) |
| PBSIII | JN439522.1 | Guerrero Negro hypersaline microbial mat | (49) |
| PBSIII | JN441783.1 | Guerrero Negro hypersaline microbial mat | (49) |
| PBSIII | JN442000.1 | Guerrero Negro hypersaline microbial mat | (49) |
| PBSIII | JN442300.1 | Guerrero Negro hypersaline microbial mat | (49) |
| PBSIII | JN444865.1 | Guerrero Negro hypersaline microbial mat | (49) |
| PBSIII | JN444875.1 | Guerrero Negro hypersaline microbial mat | (49) |
| PBSIII | JN445498.1 | Guerrero Negro hypersaline microbial mat | (49) |
| PBSIII | JN445714.1 | Guerrero Negro hypersaline microbial mat | (49) |
| PBSIII | JN448086.1 | Guerrero Negro hypersaline microbial mat | (49) |
| PBSIII | JN448989.1 | Guerrero Negro hypersaline microbial mat | (49) |
| PBSIII | JN451006.1 | Guerrero Negro hypersaline microbial mat | (49) |
| PBSIII | JN453307.1 | Guerrero Negro hypersaline microbial mat | (49) |
| PBSIII | JN453587.1 | Guerrero Negro hypersaline microbial mat | (49) |
| PBSIII | JN454087.1 | Guerrero Negro hypersaline microbial mat | (49) |
| PBSIII | JN454215.1 | Guerrero Negro hypersaline microbial mat | (49) |
| PBSIII | JN454271.1 | Guerrero Negro hypersaline microbial mat | (49) |
| PBSIII | JN454409.1 | Guerrero Negro hypersaline microbial mat | (49) |
| PBSIII | JN454752.1 | Guerrero Negro hypersaline microbial mat | (49) |
| PBSIII | JN454875.1 | Guerrero Negro hypersaline microbial mat | (49) |
| PBSIII | JN456092.1 | Guerrero Negro hypersaline microbial mat | (49) |
| PBSIII | JN456155.1 | Guerrero Negro hypersaline microbial mat | (49) |
| PBSIII | JN456385.1 | Guerrero Negro hypersaline microbial mat | (49) |
| PBSIII | JN456442.1 | Guerrero Negro hypersaline microbial mat | (49) |
| PBSIII | JN456744.1 | Guerrero Negro hypersaline microbial mat | (49) |
| PBSIII | JN456746.1 | Guerrero Negro hypersaline microbial mat | (49) |
| PBSIII | JN456995.1 | Guerrero Negro hypersaline microbial mat | (49) |
| PBSIII | JN457499.1 | Guerrero Negro hypersaline microbial mat | (49) |
| PBSIII | JN457617.1 | Guerrero Negro hypersaline microbial mat | (49) |
| PBSIII | JN457631.1 | Guerrero Negro hypersaline microbial mat | (49) |
| PBSIII | JN458796.1 | Guerrero Negro hypersaline microbial mat | (49) |
| PBSIII | JN459213.1 | Guerrero Negro hypersaline microbial mat | (49) |
| PBSIII | JN459513.1 | Guerrero Negro hypersaline microbial mat | (49) |
| PBSIII | JN459533.1 | Guerrero Negro hypersaline microbial mat | (49) |
| PBSIII | JN460882.1 | Guerrero Negro hypersaline microbial mat | (49) |
| PBSIII | JN460915.1 | Guerrero Negro hypersaline microbial mat | (49) |
| PBSIII | JN461293.1 | Guerrero Negro hypersaline microbial mat | (49) |
| PBSIII | JN461355.1 | Guerrero Negro hypersaline microbial mat | (49) |
| PBSIII | JN461492.1 | Guerrero Negro hypersaline microbial mat | (49) |
| PBSIII | JN461681.1 | Guerrero Negro hypersaline microbial mat | (49) |
| PBSIII | JN462016.1 | Guerrero Negro hypersaline microbial mat | (49) |
| PBSIII | JN462145.1 | Guerrero Negro hypersaline microbial mat | (49) |
| PBSIII | JN462590.1 | Guerrero Negro hypersaline microbial mat | (49) |
| PBSIII | JN462774.1 | Guerrero Negro hypersaline microbial mat | (49) |
| PBSIII | JN462808.1 | Guerrero Negro hypersaline microbial mat | (49) |
| PBSIII | JN463038.1 | Guerrero Negro hypersaline microbial mat | (49) |
| PBSIII | JN463063.1 | Guerrero Negro hypersaline microbial mat | (49) |
| PBSIII | JN463255.1 | Guerrero Negro hypersaline microbial mat | (49) |
| PBSIII | JN463458.1 | Guerrero Negro hypersaline microbial mat | (49) |
| PBSIII | JN463912.1 | Guerrero Negro hypersaline microbial mat | (49) |
| PBSIII | JN464055.1 | Guerrero Negro hypersaline microbial mat | (49) |
| PBSIII | JN465475.1 | Guerrero Negro hypersaline microbial mat | (49) |
| PBSIII | JN465850.1 | Guerrero Negro hypersaline microbial mat | (49) |
| PBSIII | JN465989.1 | Guerrero Negro hypersaline microbial mat | (49) |
| PBSIII | JN466425.1 | Guerrero Negro hypersaline microbial mat | (49) |
| PBSIII | JN466560.1 | Guerrero Negro hypersaline microbial mat | (49) |
| PBSIII | JN466784.1 | Guerrero Negro hypersaline microbial mat | (49) |
| PBSIII | JN467118.1 | Guerrero Negro hypersaline microbial mat | (49) |
| PBSIII | JN467222.1 | Guerrero Negro hypersaline microbial mat | (49) |
| PBSIII | JN467491.1 | Guerrero Negro hypersaline microbial mat | (49) |
| PBSIII | JN467994.1 | Guerrero Negro hypersaline microbial mat | (49) |
| PBSIII | JN469067.1 | Guerrero Negro hypersaline microbial mat | (49) |
| PBSIII | JN469146.1 | Guerrero Negro hypersaline microbial mat | (49) |
| PBSIII | JN469287.1 | Guerrero Negro hypersaline microbial mat | (49) |
| PBSIII | JN469967.1 | Guerrero Negro hypersaline microbial mat | (49) |
| PBSIII | JN470892.1 | Guerrero Negro hypersaline microbial mat | (49) |
| PBSIII | JN471270.1 | Guerrero Negro hypersaline microbial mat | (49) |
| PBSIII | JN471379.1 | Guerrero Negro hypersaline microbial mat | (49) |
| PBSIII | JN472023.1 | Guerrero Negro hypersaline microbial mat | (49) |
| PBSIII | JN472233.1 | Guerrero Negro hypersaline microbial mat | (49) |
| PBSIII | JN472287.1 | Guerrero Negro hypersaline microbial mat | (49) |
| PBSIII | JN472450.1 | Guerrero Negro hypersaline microbial mat | (49) |
| PBSIII | JN472501.1 | Guerrero Negro hypersaline microbial mat | (49) |
| PBSIII | JN472584.1 | Guerrero Negro hypersaline microbial mat | (49) |
| PBSIII | JN472843.1 | Guerrero Negro hypersaline microbial mat | (49) |
| PBSIII | JN473405.1 | Guerrero Negro hypersaline microbial mat | (49) |
| PBSIII | JN473548.1 | Guerrero Negro hypersaline microbial mat | (49) |
| PBSIII | JN473733.1 | Guerrero Negro hypersaline microbial mat | (49) |
| PBSIII | JN473997.1 | Guerrero Negro hypersaline microbial mat | (49) |
| PBSIII | JN474071.1 | Guerrero Negro hypersaline microbial mat | (49) |
| PBSIII | JN474074.1 | Guerrero Negro hypersaline microbial mat | (49) |
| PBSIII | JN475885.1 | Guerrero Negro hypersaline microbial mat | (49) |
| PBSIII | JN475939.1 | Guerrero Negro hypersaline microbial mat | (49) |
| PBSIII | JN477591.1 | Guerrero Negro hypersaline microbial mat | (49) |
| PBSIII | JN477617.1 | Guerrero Negro hypersaline microbial mat | (49) |
| PBSIII | JN477861.1 | Guerrero Negro hypersaline microbial mat | (49) |
| PBSIII | JN478379.1 | Guerrero Negro hypersaline microbial mat | (49) |
| PBSIII | JN478684.1 | Guerrero Negro hypersaline microbial mat | (49) |
| PBSIII | JN478894.1 | Guerrero Negro hypersaline microbial mat | (49) |
| PBSIII | JN479060.1 | Guerrero Negro hypersaline microbial mat | (49) |
| PBSIII | JN479311.1 | Guerrero Negro hypersaline microbial mat | (49) |
| PBSIII | JN479553.1 | Guerrero Negro hypersaline microbial mat | (49) |
| PBSIII | JN479807.1 | Guerrero Negro hypersaline microbial mat | (49) |
| PBSIII | JN480192.1 | Guerrero Negro hypersaline microbial mat | (49) |
| PBSIII | JN481557.1 | Guerrero Negro hypersaline microbial mat | (49) |
| PBSIII | JN481596.1 | Guerrero Negro hypersaline microbial mat | (49) |
| PBSIII | JN482008.1 | Guerrero Negro hypersaline microbial mat | (49) |
| PBSIII | JN482502.1 | Guerrero Negro hypersaline microbial mat | (49) |
| PBSIII | JN482537.1 | Guerrero Negro hypersaline microbial mat | (49) |
| PBSIII | JN482816.1 | Guerrero Negro hypersaline microbial mat | (49) |
| PBSIII | JN482928.1 | Guerrero Negro hypersaline microbial mat | (49) |
| PBSIII | JN483045.1 | Guerrero Negro hypersaline microbial mat | (49) |
| PBSIII | JN483516.1 | Guerrero Negro hypersaline microbial mat | (49) |
| PBSIII | JN483923.1 | Guerrero Negro hypersaline microbial mat | (49) |
| PBSIII | JN484133.1 | Guerrero Negro hypersaline microbial mat | (49) |
| PBSIII | JN484638.1 | Guerrero Negro hypersaline microbial mat | (49) |
| PBSIII | JN484694.1 | Guerrero Negro hypersaline microbial mat | (49) |
| PBSIII | JN484979.1 | Guerrero Negro hypersaline microbial mat | (49) |
| PBSIII | JN485092.1 | Guerrero Negro hypersaline microbial mat | (49) |
| PBSIII | JN485163.1 | Guerrero Negro hypersaline microbial mat | (49) |
| PBSIII | JN485223.1 | Guerrero Negro hypersaline microbial mat | (49) |
| PBSIII | JN485403.1 | Guerrero Negro hypersaline microbial mat | (49) |
| PBSIII | JN485518.1 | Guerrero Negro hypersaline microbial mat | (49) |
| PBSIII | JN485962.1 | Guerrero Negro hypersaline microbial mat | (49) |
| PBSIII | JN486583.1 | Guerrero Negro hypersaline microbial mat | (49) |
| PBSIII | JN487258.1 | Guerrero Negro hypersaline microbial mat | (49) |
| PBSIII | JN487402.1 | Guerrero Negro hypersaline microbial mat | (49) |
| PBSIII | JN487486.1 | Guerrero Negro hypersaline microbial mat | (49) |
| PBSIII | JN487499.1 | Guerrero Negro hypersaline microbial mat | (49) |
| PBSIII | JN487532.1 | Guerrero Negro hypersaline microbial mat | (49) |
| PBSIII | JN487645.1 | Guerrero Negro hypersaline microbial mat | (49) |
| PBSIII | JN488480.1 | Guerrero Negro hypersaline microbial mat | (49) |
| PBSIII | JN489111.1 | Guerrero Negro hypersaline microbial mat | (49) |
| PBSIII | JN489132.1 | Guerrero Negro hypersaline microbial mat | (49) |
| PBSIII | JN489207.1 | Guerrero Negro hypersaline microbial mat | (49) |
| PBSIII | JN489492.1 | Guerrero Negro hypersaline microbial mat | (49) |
| PBSIII | JN489755.1 | Guerrero Negro hypersaline microbial mat | (49) |
| PBSIII | JN489828.1 | Guerrero Negro hypersaline microbial mat | (49) |
| PBSIII | JN490576.1 | Guerrero Negro hypersaline microbial mat | (49) |
| PBSIII | JN490626.1 | Guerrero Negro hypersaline microbial mat | (49) |
| PBSIII | JN490938.1 | Guerrero Negro hypersaline microbial mat | (49) |
| PBSIII | JN491724.1 | Guerrero Negro hypersaline microbial mat | (49) |
| PBSIII | JN492455.1 | Guerrero Negro hypersaline microbial mat | (49) |
| PBSIII | JN493688.1 | Guerrero Negro hypersaline microbial mat | (49) |
| PBSIII | JN493903.1 | Guerrero Negro hypersaline microbial mat | (49) |
| PBSIII | JN494266.1 | Guerrero Negro hypersaline microbial mat | (49) |
| PBSIII | JN494565.1 | Guerrero Negro hypersaline microbial mat | (49) |
| PBSIII | JN494837.1 | Guerrero Negro hypersaline microbial mat | (49) |
| PBSIII | JN495216.1 | Guerrero Negro hypersaline microbial mat | (49) |
| PBSIII | JN495246.1 | Guerrero Negro hypersaline microbial mat | (49) |
| PBSIII | JN495351.1 | Guerrero Negro hypersaline microbial mat | (49) |
| PBSIII | JN495748.1 | Guerrero Negro hypersaline microbial mat | (49) |
| PBSIII | JN495925.1 | Guerrero Negro hypersaline microbial mat | (49) |
| PBSIII | JN496352.1 | Guerrero Negro hypersaline microbial mat | (49) |
| PBSIII | JN496561.1 | Guerrero Negro hypersaline microbial mat | (49) |
| PBSIII | JN497018.1 | Guerrero Negro hypersaline microbial mat | (49) |
| PBSIII | JN497339.1 | Guerrero Negro hypersaline microbial mat | (49) |
| PBSIII | JN497694.1 | Guerrero Negro hypersaline microbial mat | (49) |
| PBSIII | JN498076.1 | Guerrero Negro hypersaline microbial mat | (49) |
| PBSIII | JN498550.1 | Guerrero Negro hypersaline microbial mat | (49) |
| PBSIII | JN499226.1 | Guerrero Negro hypersaline microbial mat | (49) |
| PBSIII | JN499470.1 | Guerrero Negro hypersaline microbial mat | (49) |
| PBSIII | JN499535.1 | Guerrero Negro hypersaline microbial mat | (49) |
| PBSIII | JN501162.1 | Guerrero Negro hypersaline microbial mat | (49) |
| PBSIII | JN501358.1 | Guerrero Negro hypersaline microbial mat | (49) |
| PBSIII | JN501901.1 | Guerrero Negro hypersaline microbial mat | (49) |
| PBSIII | JN502407.1 | Guerrero Negro hypersaline microbial mat | (49) |
| PBSIII | JN502596.1 | Guerrero Negro hypersaline microbial mat | (49) |
| PBSIII | JN503186.1 | Guerrero Negro hypersaline microbial mat | (49) |
| PBSIII | JN503546.1 | Guerrero Negro hypersaline microbial mat | (49) |
| PBSIII | JN503922.1 | Guerrero Negro hypersaline microbial mat | (49) |
| PBSIII | JN504121.1 | Guerrero Negro hypersaline microbial mat | (49) |
| PBSIII | JN504246.1 | Guerrero Negro hypersaline microbial mat | (49) |
| PBSIII | JN504429.1 | Guerrero Negro hypersaline microbial mat | (49) |
| PBSIII | JN504597.1 | Guerrero Negro hypersaline microbial mat | (49) |
| PBSIII | JN505128.1 | Guerrero Negro hypersaline microbial mat | (49) |
| PBSIII | JN505231.1 | Guerrero Negro hypersaline microbial mat | (49) |
| PBSIII | JN505616.1 | Guerrero Negro hypersaline microbial mat | (49) |
| PBSIII | JN505722.1 | Guerrero Negro hypersaline microbial mat | (49) |
| PBSIII | JN505952.1 | Guerrero Negro hypersaline microbial mat | (49) |
| PBSIII | JN506689.1 | Guerrero Negro hypersaline microbial mat | (49) |
| PBSIII | JN506712.1 | Guerrero Negro hypersaline microbial mat | (49) |
| PBSIII | JN507118.1 | Guerrero Negro hypersaline microbial mat | (49) |
| PBSIII | JN507147.1 | Guerrero Negro hypersaline microbial mat | (49) |
| PBSIII | JN507609.1 | Guerrero Negro hypersaline microbial mat | (49) |
| PBSIII | JN507683.1 | Guerrero Negro hypersaline microbial mat | (49) |
| PBSIII | JN508129.1 | Guerrero Negro hypersaline microbial mat | (49) |
| PBSIII | JN509437.1 | Guerrero Negro hypersaline microbial mat | (49) |
| PBSIII | JN509611.1 | Guerrero Negro hypersaline microbial mat | (49) |
| PBSIII | JN510461.1 | Guerrero Negro hypersaline microbial mat | (49) |
| PBSIII | JN510798.1 | Guerrero Negro hypersaline microbial mat | (49) |
| PBSIII | JN511148.1 | Guerrero Negro hypersaline microbial mat | (49) |
| PBSIII | JN512199.1 | Guerrero Negro hypersaline microbial mat | (49) |
| PBSIII | JN513557.1 | Guerrero Negro hypersaline microbial mat | (49) |
| PBSIII | JN514210.1 | Guerrero Negro hypersaline microbial mat | (49) |
| PBSIII | JN514570.1 | Guerrero Negro hypersaline microbial mat | (49) |
| PBSIII | JN514726.1 | Guerrero Negro hypersaline microbial mat | (49) |
| PBSIII | JN514945.1 | Guerrero Negro hypersaline microbial mat | (49) |
| PBSIII | JN515070.1 | Guerrero Negro hypersaline microbial mat | (49) |
| PBSIII | JN515601.1 | Guerrero Negro hypersaline microbial mat | (49) |
| PBSIII | JN515934.1 | Guerrero Negro hypersaline microbial mat | (49) |
| PBSIII | JN516944.1 | Guerrero Negro hypersaline microbial mat | (49) |
| PBSIII | JN518822.1 | Guerrero Negro hypersaline microbial mat | (49) |
| PBSIII | JN519911.1 | Guerrero Negro hypersaline microbial mat | (49) |
| PBSIII | JN520304.1 | Guerrero Negro hypersaline microbial mat | (49) |
| PBSIII | JN520470.1 | Guerrero Negro hypersaline microbial mat | (49) |
| PBSIII | JN520812.1 | Guerrero Negro hypersaline microbial mat | (49) |
| PBSIII | JN522646.1 | Guerrero Negro hypersaline microbial mat | (49) |
| PBSIII | JN523553.1 | Guerrero Negro hypersaline microbial mat | (49) |
| PBSIII | JN524707.1 | Guerrero Negro hypersaline microbial mat | (49) |
| PBSIII | JN525083.1 | Guerrero Negro hypersaline microbial mat | (49) |
| PBSIII | JN525187.1 | Guerrero Negro hypersaline microbial mat | (49) |
| PBSIII | JN526009.1 | Guerrero Negro hypersaline microbial mat | (49) |
| PBSIII | JN526324.1 | Guerrero Negro hypersaline microbial mat | (49) |
| PBSIII | JN526787.1 | Guerrero Negro hypersaline microbial mat | (49) |
| PBSIII | JN526849.1 | Guerrero Negro hypersaline microbial mat | (49) |
| PBSIII | JN527904.1 | Guerrero Negro hypersaline microbial mat | (49) |
| PBSIII | JN528379.1 | Guerrero Negro hypersaline microbial mat | (49) |
| PBSIII | JN528459.1 | Guerrero Negro hypersaline microbial mat | (49) |
| PBSIII | JN528779.1 | Guerrero Negro hypersaline microbial mat | (49) |
| PBSIII | JN529008.1 | Guerrero Negro hypersaline microbial mat | (49) |
| PBSIII | JN529436.1 | Guerrero Negro hypersaline microbial mat | (49) |
| PBSIII | JN529785.1 | Guerrero Negro hypersaline microbial mat | (49) |
| PBSIII | JN530192.1 | Guerrero Negro hypersaline microbial mat | (49) |
| PBSIII | JN530214.1 | Guerrero Negro hypersaline microbial mat | (49) |
| PBSIII | JN530419.1 | Guerrero Negro hypersaline microbial mat | (49) |
| PBSIII | JN530495.1 | Guerrero Negro hypersaline microbial mat | (49) |
| PBSIII | JN530544.1 | Guerrero Negro hypersaline microbial mat | (49) |
| PBSIII | JN530549.1 | Guerrero Negro hypersaline microbial mat | (49) |
| PBSIII | JN530689.1 | Guerrero Negro hypersaline microbial mat | (49) |
| PBSIII | JN530781.1 | Guerrero Negro hypersaline microbial mat | (49) |
| PBSIII | JN530880.1 | Guerrero Negro hypersaline microbial mat | (49) |
| PBSIII | JN531175.1 | Guerrero Negro hypersaline microbial mat | (49) |
| PBSIII | JN531274.1 | Guerrero Negro hypersaline microbial mat | (49) |
| PBSIII | JN531305.1 | Guerrero Negro hypersaline microbial mat | (49) |
| PBSIII | JN531323.1 | Guerrero Negro hypersaline microbial mat | (49) |
| PBSIII | JN532077.1 | Guerrero Negro hypersaline microbial mat | (49) |
| PBSIII | JN532186.1 | Guerrero Negro hypersaline microbial mat | (49) |
| PBSIII | JN532365.1 | Guerrero Negro hypersaline microbial mat | (49) |
| PBSIII | JN532732.1 | Guerrero Negro hypersaline microbial mat | (49) |
| PBSIII | JN533029.1 | Guerrero Negro hypersaline microbial mat | (49) |
| PBSIII | JN533031.1 | Guerrero Negro hypersaline microbial mat | (49) |
| PBSIII | JN533509.1 | Guerrero Negro hypersaline microbial mat | (49) |
| PBSIII | JN533519.1 | Guerrero Negro hypersaline microbial mat | (49) |
| PBSIII | JN533729.1 | Guerrero Negro hypersaline microbial mat | (49) |
| PBSIII | JN534021.1 | Guerrero Negro hypersaline microbial mat | (49) |
| PBSIII | JN534121.1 | Guerrero Negro hypersaline microbial mat | (49) |
| PBSIII | JN535553.1 | Guerrero Negro hypersaline microbial mat | (49) |
| PBSIII | JN535609.1 | Guerrero Negro hypersaline microbial mat | (49) |
| PBSIII | JN535698.1 | Guerrero Negro hypersaline microbial mat | (49) |
| PBSIII | JN535836.1 | Guerrero Negro hypersaline microbial mat | (49) |
| PBSIII | JN536291.1 | Guerrero Negro hypersaline microbial mat | (49) |
| PBSIII | JN536400.1 | Guerrero Negro hypersaline microbial mat | (49) |
| PBSIII | JN536661.1 | Guerrero Negro hypersaline microbial mat | (49) |
| PBSIII | JN537553.1 | Guerrero Negro hypersaline microbial mat | (49) |
| PBSIII | JN538214.1 | Guerrero Negro hypersaline microbial mat | (49) |
| PBSIII | JN538609.1 | Guerrero Negro hypersaline microbial mat | (49) |
| PBSIII | EU542529.1 | sediment and soil slurry | (142) |
| PBSIII | JN229995.1 | subseafloor sediment at Porcupine seabight | (143) |
| PBSIII | DQ289929.2 | South Atlantic Bight Permeable Shelf Sediment | (144) |
| PBSIII | AB094954.1 | subseafloor sediments from the Sea of Okhotsk | (145) |
| PBSIII | AB177136.1 | Methane hydrate-bearing deep marine sediments on the Pacific Ocean Margin | (146) |
| PBSIII | AB177152.1 | Methane hydrate-bearing deep marine sediments on the Pacific Ocean Margin | (146) |
| PBSIII | AB177208.1 | Methane hydrate-bearing deep marine sediments on the Pacific Ocean Margin | (146) |
| PBSIII | EU245422.1 | Puerto Rico: Cabo Rojo, Candeleria lagoon | (84) |
| PBSIII | EU245437.1 | Puerto Rico: Cabo Rojo, Candeleria lagoon | (84) |
| PBSIII | EU245516.1 | Puerto Rico: Cabo Rojo, Candeleria lagoon | (84) |
| PBSIII | EU245566.1 | Puerto Rico: Cabo Rojo, Candeleria lagoon | (84) |
| PBSIII | EU245573.1 | Puerto Rico: Cabo Rojo, Candeleria lagoon | (84) |
| PBSIII | EU245637.1 | Puerto Rico: Cabo Rojo, Candeleria lagoon | (84) |
| PBSIII | EU245880.1 | Puerto Rico: Cabo Rojo, Candeleria lagoon | (84) |
| PBSIII | EU245919.1 | Puerto Rico: Cabo Rojo, Candeleria lagoon | (84) |
| PBSIII | EU245973.1 | Puerto Rico: Cabo Rojo, Candeleria lagoon | (84) |
| PBSIII | EU246239.1 | Puerto Rico: Cabo Rojo, Candeleria lagoon | (84) |
| PBSIII | AB656833.1 | rice paddy soil | (97) |
| PBSIII | DQ330720.1 | A solar saltworks located at Guerrero Negro hypersaline microbial mat | (147) |
| PBSIII | EU385726.1 | subseafloor sediment of the South China sea | (148) |
| PBSIII | EU385764.1 | subseafloor sediment of the South China sea | (148) |
| PBSIII | EU385924.1 | subseafloor sediment of the South China sea | (148) |
| PBSIII | EU385954.1 | subseafloor sediment of the South China sea | (148) |
| PBSIII | AB433146.1 | Deep subseafloor sediments at the Brazos-Trinity Basin, the Gulf of Mexico | (149) |
| PBSIII | AB305512.1 | Hydrothermal sediments at the Yonaguni Knoll IV hydrothermal field in the Southern Okinawa trough | (130) |
| PBSIII | AB583304.1 | Deep-sea sediments, a sediment core obtained from the Ogasawara Trench at a water depth of 9760 m | (150) |
| PBSIII | AB583312.1 | Deep-sea sediments, a sediment core obtained from the Ogasawara Trench at a water depth of 9760 m | (150) |
| PBSIII | AB583315.1 | Deep-sea sediments, a sediment core obtained from the Ogasawara Trench at a water depth of 9760 m | (150) |
| PBSIII | FJ484305.1 | wall biomat sample in El Zacaton at 82m depth | (61) |
| PBSIII | FJ484342.1 | wall biomat sample in El Zacaton at 82m depth | (61) |
| PBSIII | EU491142.1 | seafloor lavas from the Loi'hi Seamount | (72) |
| PBSIII | DQ996966.1 | South China Sea, deep-sea sediment | (151) |
| PBSIII | DQ996967.1 | South China Sea, deep-sea sediment | (151) |
| PBSIII | AM943581.1 | Carbonaceous sediments from a hypersaline lagoon | (152) |
| PBSIII | GQ348493.1 | Saanich Inlet, 200 m depth, Saanich Peninsula and the Malahat highlands of Vancouver Island | (153) |
| PBSIII | JQ968714.1 | Grasse River sediment | Unpublished |
| PBSIII | JQ925046.1 | cold seep sediment from oxygen mimimum zone in Pakistan Margin | Unpublished |
| PBSIII | FJ813575.1 | marine sediment | Unpublished |
| PBSIII | GQ267075.1 | Mothra sediments in Juan de Fuca | Unpublished |
| PBSIII | DQ004678.1 | Mud Volcano Sediments in the Gulf of Cadiz | Unpublished |
| PBSIII | JX001052.1 | subseafloor sediment at the Deformation front | Unpublished |
| PBSIII | JX001158.1 | subseafloor sediment at the Deformation front | Unpublished |
| PBSIII | JQ817758.1 | subseafloor sediment at the Kaoping Canyon | Unpublished |
| PBSIII | JQ817789.1 | subseafloor sediment at the Kaoping Canyon | Unpublished |
| PBSIII | JN123535.1 | subseafloor sediment at the Tainan Ridge, Taiwan | Unpublished |
| PBSIII | JN123574.1 | subseafloor sediment at the Tainan Ridge, Taiwan | Unpublished |
| PBSIII | JN123579.1 | subseafloor sediment at the Tainan Ridge, Taiwan | Unpublished |
| PBSIII | AB645311.1 | subseafloor sediments off Shimokita Peninsula | Unpublished |
| PBSIII | AB645312.1 | subseafloor sediments off Shimokita Peninsula | Unpublished |
| PBSIII | AB645313.1 | subseafloor sediments off Shimokita Peninsula | Unpublished |
| PBSIII | AB645315.1 | subseafloor sediments off Shimokita Peninsula | Unpublished |
| PBSIII | AB645316.1 | subseafloor sediments off Shimokita Peninsula | Unpublished |
| PBSIII | AB645317.1 | subseafloor sediments off Shimokita Peninsula | Unpublished |
| PBSIII | KC545703.1 | oxycline region of a coastal marine water column | Unpublished |
| PBSIII | HQ330613.1 | Lake Wivenhoe, Australia Sediment | Unpublished |
| PBSIII | JX472335.1 | anoxic lacustrine sediment, South Patagonia | Unpublished |
| PBSIII | JX472347.1 | anoxic lacustrine sediment, South Patagonia | Unpublished |
| PBSIII | FJ716430.1 | Frasassi cave system, anoxic terrestrial | Unpublished |
| PNO_1 | JQ580440.1 | sediments from Rodas Beach polluted with crude oil | (110) |
| PNO_1 | JQ580443.1 | sediments from Rodas Beach polluted with crude oil | (110) |

Table B. Total number of glycosyl hydrolases (GHs), polysaccharide lyases (PLs), and carbohydrate esterases (CEs) in the 2 most complete Latescibacteria SAGs compared to other lignocellulolytic and alginolytic organisms.

| Species Name | GH | CE | PL | GH/Mb* | PL/Mb* |
| --- | --- | --- | --- | --- | --- |
| “*Latescibacteria*” SAG S-B13 | 21 | 13 | 16 | 14.09 | 10.74 |
| “*Latescibacteria*” SAG S-E07 | 29 | 14 | 18 | 12.61 | 7.83 |
| *Saccharophagus degradans* 2-40 | 137 | 15 | 36 | 27.08 | 7.11 |
| *Opitutaceae* bacterium TAV5 | 219 | 24 | 35 | 29.55 | 4.72 |
| *Formosa agariphila* KMM 3901 | 96 | 8 | 15 | 22.70 | 3.55 |
| *Fibrobacter succinogenes* subsp. *succinogenes* S85 | 100 | 17 | 12 | 26.04 | 3.13 |
| *Clostridium cellulovorans* 743B | 117 | 21 | 15 | 22.24 | 2.85 |
| *Bacteroides thetaiotaomicron* VPI-5482 | 268 | 18 | 17 | 42.81 | 2.72 |
| *Brevundimonas subvibrioides* ATCC 15264 | 30 | 7 | 8 | 8.70 | 2.32 |
| *Clostridium phytofermentans* ISDg | 120 | 14 | 10 | 24.74 | 2.06 |
| *Ruminococcus albus* 7 | 97 | 18 | 7 | 21.65 | 1.56 |
| *Anaerocellum thermophilum* DSM 6725 | 60 | 5 | 4 | 20.55 | 1.37 |
| *Clostridium thermocellum* ATCC 27405 | 75 | 16 | 4 | 21.07 | 1.12 |
| *Gemmatimonadetes bacterium* KBS708 | 135 | 25 | 5 | 25.42 | 0.94 |
| *Yersinia enterocolitica* subsp. *enterocolitica* 8081 | 40 | 6 | 4 | 8.55 | 0.85 |
| *Caldicellulosiruptor obsidiansis* | 54 | 5 | 2 | 21.34 | 0.79 |
| *Escherichia coli* CFT073 | 42 | 9 | 2 | 8.40 | 0.40 |
| *Acidobacterium capsulatum* | 87 | 12 | 1 | 21.07 | 0.24 |
| *Thermoanaerobacterium thermosaccharolyticum* | 48 | 7 | 0 | 17.20 | 0.00 |
| *Thermoanaerobacterium xylanolyticum* LX-11 | 33 | 8 | 0 | 13.04 | 0.00 |

*GH/Mb and PL/Mb are the ratio of the total number of GHs and PLs per Mb of the corresponding genome.

Table C. Number of peptidases belonging to various Merops peptidase families identified in “*Latescibacteria*” genomes and their possible physiological roles.

| Merops Family | Genomes | | Annotation | Possible physiological function |
| --- | --- | --- | --- | --- |
|  | S-E07 | S-B13 |  |  |
| A08 | 1 | 1 | Signal peptidase II [EC: 3.4.23.36] | Protein activation |
| A24 | 1 | 0 | Type IV prepilin peptidase 1 [EC: 3.4.23.43] | Protein activation |
| A31 | 2 | 1 | Hydrogenase 3 maturation protease [EC: 3.4.23.51] | Protein activation |
| C14 | 2 | 0 | Apoptosis caspase | Protein activation |
| C25 | 1 | 0 | Gingipain | Matrix degradation |
| C39 | 0 | 2 | Bacteriocin processing* | Activation and transport of peptide AB |
| C45 | 1 | 0 | Isopenicillin-N N-acyltransferase [EC: 2.3.1.164]* | Protein modification |
| M06 | 2 | 0 | Metalloprotease | Possibly nutritional, non-specific. |
| M10 | 0 | 1 | Matrixin | Matrix degradation |
| M16 | 3 | 4 | Signal peptidase | Protein activation |
| M19 | 1 | 2 | Membrane dipeptidase [EC: 3.4.13.19] | Possibly nutritional |
| M20 | 2 | 2 | Metalloprotease | Hydrolysis of the late products of protein degradation so as to complete the conversion of proteins to free amino acids. Possibly nutritional, non-specific. |
| M22 | 2 | 1 | Hydrogenase maturation protease | Protein activation |
| M23 | 7 | 3 | Membrane-bound metallopeptidase | Bacterial cell wall lysis. Possibly defensive or feeding mechanism |
| M24 | 1 | 1 | Methionyl aminopeptidase [EC: 3.4.11.18] | Removal of the initiating methionine of many proteins |
| M28 | 0 | 5 | Predicted aminopeptidase, Iap family | Removal of amino acids from N-terminus. Possibly nutritional |
| M41 | 1 | 1 | Membrane protease FtsH catalytic subunit [EC: 3.4.24.-] | Degrading unneeded or damaged membrane proteins |
| M48 | 0 | 1 | Endopeptidase | Degradation of abnormal proteins |
| M50 | 2 | 1 | Intra-membrane protease | Protein activation or possibly nutritional |
| M56 | 2 | 3 | Potential penicillin-binding protein required for induction of beta-lactamase | Antirepressor regulating drug resistance |
| S01 | 2 | 5 | Trypsin-like serine proteases | Possibly nutritional, non-specific proteolysis |
| S08 | 4 | 1 | Subtilisin-like serine proteases | Possibly nutritional, non-specific proteolysis |
| S09 | 2 | 0 | Non-specific metalloprotease | Degradation of biologically active peptides. Possibly nutritional |
| S24 | 0 | 2 | RecA-mediated autopeptidases | SOS-response transcriptional repressors |
| S26 | 1 | 0 | Signal peptidase I [EC: 3.4.21.89] | Protein activation |
| S41 | 1 | 1 | C-terminal processing peptidase-3 | Degradation of incorrectly synthesized proteins |
| S49 | 0 | 1 | Signal peptide peptidase A. | Degrade the signal peptide cleaved by signal peptidases. Possibly nutritional |
| S54 | 1 | 1 | Rhomboid intra-membrane protease | Protein activation or possibly nutritional |
| T01 | 1 | 1 | ATP-dependent protease HslVU, peptidase subunit | Turnover of intracellular proteins |
| T03 | 1 | 0 | Gamma-glutamyltransferase. | Degradation of glutathione by cleavage of the gamma-glutamyl bond |
| U62 | 3 | 0 | Predicted Zn-dependent protease | Possibly nutritional, non-specific proteolysis |
| Total | 47 | 41 |  |  |

*: Biosynthetic genes for the related antibiotic were not identified in the genome. Possibly performing a different function.

Supplementary Figure Legends.

**Figure A**. Total number of “*Latescibacteria*” genes belonging to the different families of glycosyl hydrolases (GHs) and polysaccharide lyases (PLs) shown on the X-axis for SAGs S-E07 (◼) and S-B13 (☐).

**Figure B**. Schematic representation of polymers shown in Table 2.

Figure A

1. Figure B
2.
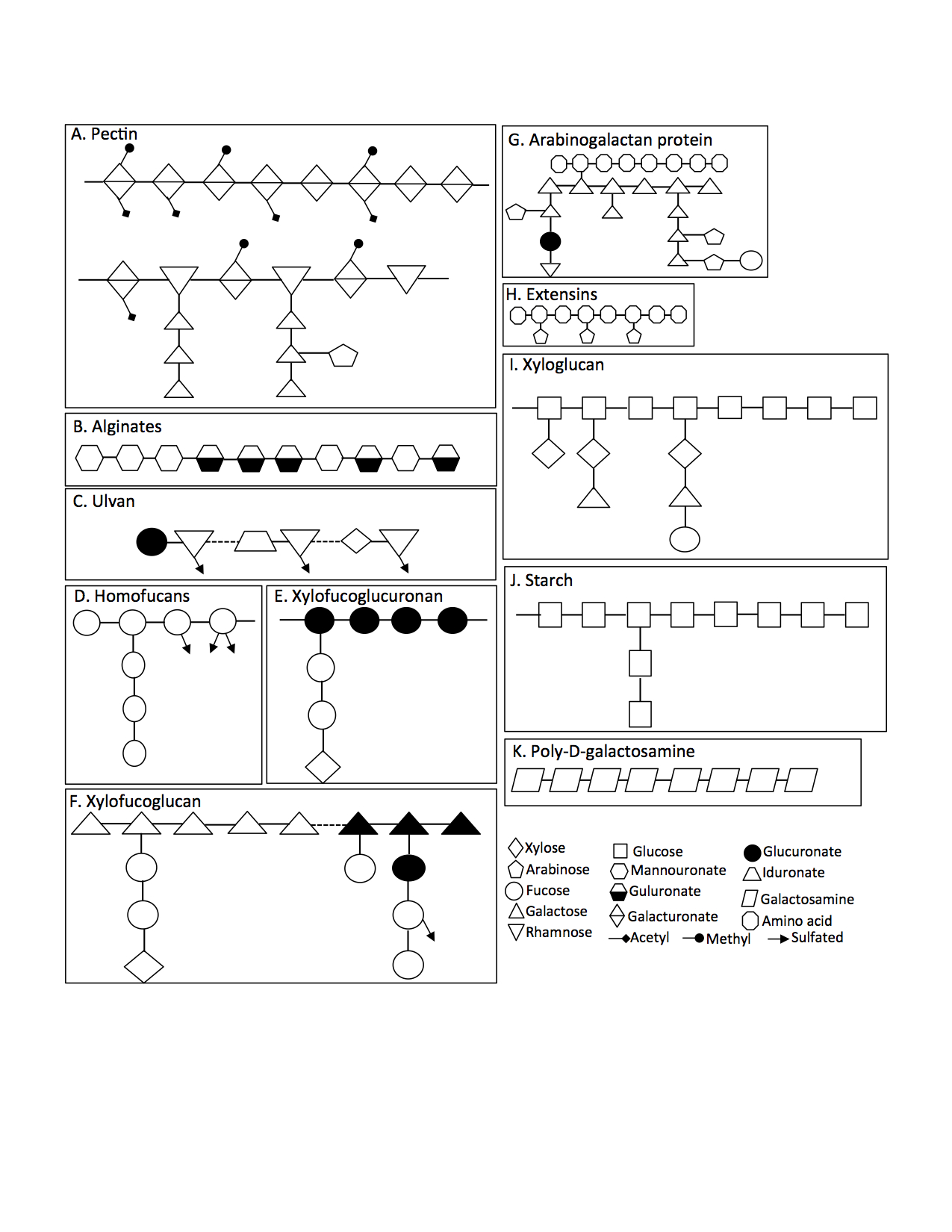

Supplement: S1 File — Table A. Genbank accession numbers, candidate order, and study site of all near-full-length 16S rRNA gene sequences affiliated with “Latescibacteria” that were used to construct phylogenetic trees shown in Fig 1. Table B. Total number of glycosyl hydrolases (GHs), polysaccharide lyases (PLs), and carbohydrate esterases (CEs) in the two most complete “Latescibacteria” SAGs compared to other lignocellulolytic and alginolytic organisms. Table C. Number of peptidases belonging to various Merops peptidase families identified in “Latescibacteria” genomes and their possible physiological roles. Figure A. Total number of “Latescibacteria” genes belonging to the different families of glycosyl hydrolases (GHs) and polysaccharide lyases (PLs) shown on the X-axis for SAGs S-E07 and S-B13. Figure B. Schematic representation of polymers shown in Table 2. (DOCX) [file pone.0127499.s001.docx]
